# Practical preparation of unsaturated very-long-chain fatty acids (VLCFAs) and very-long-chain alkene pollinator attractants

**DOI:** 10.1038/s41598-024-70598-x

**Published:** 2024-08-24

**Authors:** Björn Bohman, Aylin J. Bersch, Gavin R. Flematti, Philipp M. Schlüter

**Affiliations:** 1 School of Molecular Sciences, University of Western Australia, Perth, Australia; 2 Department of Plant Protection Biology, Swedish University of Agricultural Sciences, Lomma, Sweden; 3 Department of Plant Evolutionary Biology, Institute of Biology, University of Hohenheim, Stuttgart, Germany

**Keywords:** Fatty acids, Chemical ecology, Synthetic chemistry methodology

## Abstract

To prepare very-long-chain fatty acids and alkenes (VLCFAs and VLC alkenes) that are known pollinator attractants for sexually deceptive orchids, and biosynthetic precursors thereof, we applied a methodology allowing us to prepare monounsaturated VLCFAs with chain lengths up to 28 carbons and VLC alkenes up to 31 carbons. We implemented a coupling reaction between commercially available terminal alkynes and bromoalkanoic acids to prepare VLCFAs, allowing the products to be formed in two steps. For VLC alkenes, with many alkyltriphenylphosphonium bromides commercially available, we applied a Wittig reaction approach to prepare (*Z*)-configured monoenes in a single step. Using practical methods not requiring special reagents or equipment, we obtained 11 VLCFAs in > 90% isomeric purity, and 17 VLC alkenes in > 97% isomeric purity. Such general and accessible synthetic methods are essential for chemical ecology and biochemistry research to aid researchers in unambiguously identifying isolated semiochemicals and their precursors.

## Introduction

Very-long-chain fatty acids (VLCFAs) have been identified from a wide range of organisms. Their roles as precursors to bioactive compounds are best known from insects^1^ and plants^2^, where they are known precursors to many bioactive fatty-acid derivatives, such as primary alcohols, aldehydes and esters. The definition of VLCFAs varies, but is often described as a minimum of 18–23 carbon atoms^3^.

Among plants, orchids have developed sophisticated and often highly specialised strategies for reproduction as an adaptation to diverse habitats, and interactions with pollinators^4^. Sexual deception is one of the most intriguing pollination strategies that can be found in orchids around the globe, for example in the genera *Ophrys* in Europe*, Chiloglottis* in Australia or *Disa* in South Africa^4^. The orchid flowers mimic characteristics of the females of specific insect species serving as pollinators. Instead of offering reward for pollination, like nectar or pollen, sexually deceptive flowers typically display olfactory (chemical traits), optical (visual traits), and tactile (e.g. surface structure) features which imitate the female. Those traits lure male pollinators to the deceptive flowers and lead to pseudo-copulation during which pollen packets (pollinia) can be transferred^4^.

Chemical traits have repeatedly been shown to be of outstanding importance in such sexually deceptive plant-pollinator interactions^4^. For example, the role of very-long-chain (VLC) alkenes in pollinator attraction has been investigated extensively in *Ophrys* orchids in Europe, and in *Pterostylis* orchids in Australia, with both showing that floral odour plays a particularly important role in attracting specific pollinators^5,6^. VLC alkenes have been shown to contribute to the complex blend of floral scents of these orchids, closely resembling the pheromones of female insects through precise combinations and ratios^7^.

Alkenes with chain length C_21_-C_31_, which in insects are generally derived biosynthetically by β-oxidation of VLCFAs, are common as cuticular hydrocarbons in many insects^1^, and sexual pheromones for a range of orchid-pollinating bees^8^. In plants, alkanes and alkenes are thought to be derived from saturated and unsaturated VLCFAs, respectively^9,10^. Similar to VLCFAs, there are many examples of VLC alkenes identified as natural products, but pure (*Z*)-alkenes are generally not commercially available, and synthetic standards seldom used to unambiguously confirm the regioisomeric configuration of natural products.

**Scheme 1:**
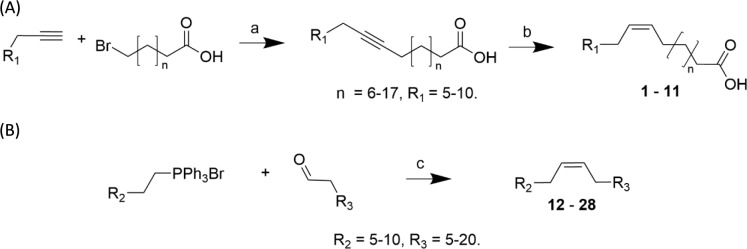
Synthesis of VLCFAs (**1**–**11**) (**A**), and VLC alkenes (**12**–**28**) (**B**). a) *n*-BuLi, HMPA, b) H_2_, Lindlar cat., EtOAc, c) LiHMDS, THF/HMPA 4:1. Un-optimised yields for step a: 44–95%, step b: 84–95%, step c: 13–92%.

Despite many reported studies on the presence of unsaturated VLCFAs in nature, there are few examples that outline practical, effective methods to prepare these in high isomeric purity to provide standards for unambiguous identification and bioassays. In addition, conventional methods normally used to prepare fatty acids of short to medium length, such as Grignard-type cross couplings are generally not applicable to VLCFAs^11^. In our work on elucidating the detailed biosynthesis of *Ophrys* pollinator attractants, we required a range of C_20_-C_28_-fatty acids, with (*Z*)-configured double bonds in Δ7, Δ9 and Δ12-positions. We applied a methodology allowing us to prepare monosaturated VLCFAs with chain lengths up to 28 carbons, using a practical method not requiring special reagents or equipment, with products obtained at least 90% pure (Scheme 1A).

VLC alkenes can be obtained by a biomimetic decarboxylation of VLCFAs^12^, but we also wanted access to an affordable, fast, direct route to these compounds. Larger amounts in high purity are necessary for conducting field bioassays within our orchid pollination research program, including structure–activity studies similar to previous studies with actetophenones^13^, drakolides^14^ and hydroxymethylpyrazines^15^ in Australia. Consequently, we optimised a Wittig-based methodology allowing us to prepare monosaturated compounds with chain lengths up to 31 carbons, with excellent isomeric purity (> 97%, Scheme 1B).

## Materials and methods

According to standard procedures, solvents were dried over molecular sieves and distilled when necessary. All reactions were conducted under a positive pressure of dry nitrogen on a scale of 1–3 mmol. Anhydrous reactions were conducted in oven-dried glassware and reagents that are sensitive to air or moisture were transferred using nitrogen-purged disposable syringes. Where applicable, compounds were purified by Medium Pressure Liquid Chromatography (MPLC, Separo, Sweden) on silica gel (40–63 mesh) using the solvent system specified. All reagents, including bromoalkanoic acids and alkyltriphenylphosphonium bromides, were purchased from Merck (Australia), ABCR (Germany) or AmBeed (USA). It must be noted that hexamethylphosphoramide (HMPA) is carcinogenic, and care must be taken to handle this solvent appropriately in a fume hood; all residues need to be treated with hydrochloric acid before disposal^16^. In this work we did not explore alternative solvents, such as *N*,*N*′-dimethylpropyleneurea (DMPU)^17^ and 1,3-dimethyl-2-imidazolidinone (DMI)^18^, however we note that DMPU is also mutagenic in *Drosophila* and a suspected carcinogen^19^, while DMI forms precipitates in mixtures with THF at the temperatures required for our VLC alkene synthesis protocol^20^. THF without co-solvent did not afford acceptable isomeric purity of our products in preliminary studies (data not shown).

General method for VLCFA synthesis (1–3 mmol scale, modified from^21^): *n*-BuLi (1.6 M in hexanes, 3 equiv.) was added to 1-tridecyne (3 equiv.) in HMPA (3 mL/mmol bromoalkanoic acid) at 0 °C. After 15 min, bromoalkanoic acid (1 equiv.) in HMPA (2.5 mL/mmol) was added dropwise at the same temperature. The reaction mixture was stirred at room temperature overnight. The red-brown solution was quenched with ice-cold aqueous HCl (1 M), followed by acidification with HCl (6 M), then extracted three times with diethyl ether, washed twice with aqueous HCl (1 M), twice with water and twice with brine, dried over magnesium sulphate and concentrated in vacuo to a crude product that was purified by MPLC (gradient from hexane to ethyl acetate). This intermediate was hydrogenated at room temperature with Lindlar catalyst (100 mg/mmol) and H_2_ (1 Atm) in ethyl acetate (100 mL/mmol).

General method for VLC alkene synthesis (1–3 mmol scale, modified from^11^): Alkyltriphenylphosphoniumbromide (1.8 equiv.) was dissolved in THF/HMPA (4:1, 3 mL/mmol aldehyde) and cooled on an ice-bath. LiHMDS (1.0 M in THF, 1.8 equiv.) was added and after 10 min the mixture was cooled on dry ice/acetone and dodecanal (1 equiv.) was added and the reaction mixture was stirred at − 78 °C for 1 h. Saturated ammonium chloride was added and the product was extracted three times with ethyl acetate, washed with water and brine, dried over magnesium sulphate, concentrated in vacuo and purified with MPLC (hexanes).

## Results

### Very-long-chain fatty acid (VLCFA) synthesis

After several reported Grignard coupling approaches^11^ failed in our hands, we explored the somewhat overlooked coupling reaction between terminal alkynes and bromoalkanoic acids^21^. Since most terminal alkynes and bromoalkanoic acids are commercially available, the products can be formed in two steps, with only one purification step. The products were generally obtained in fair to excellent yields (Table 1) and found to be at least 90% isomerically pure as determined by ^13^C-NMR and ^1^H-NMR with particular focus on the signals for the allylic carbons, which are clearly distinguishable between (*E*) and (*Z*)-isomers by ^13^C NMR^22,23^.

**Table 1:** Synthesis details for VLCFAs. NMR data for numbered compounds are available in SI.

Alkyne	Bromoalkanoic acid	VLCFA	Yield (%)
1-octyne	12-bromododecanoic acid	(13*Z*)-eicosenoic acid (**1**)	˃ 95
1-octyne	14-bromotetrdecanoic acid	(15*Z*)-docosenoic acid (**2**)	86
1-octyne	16-bromohexadecanoic acid	(17*Z*)-tetracosenoic acid (**3**)	80
1-octyne	18-bromooctadecanoic acid	(19*Z*)-hexacosenoic acid (**4**)	78
1-octyne	20-bromoeicosenoic acid	(21*Z*)-octacosenoic acid (**5**)	65
1-decyne	16-bromohexadecanoic acid	(17*Z*)-hexacosenoic acid (**6**)	71
1-decyne	18-bromooctadecenoic acid	(19*Z*)-octacosenoic acid (**7**)	90
1-tridecyne	9-bromononanoic acid	(10*Z*)-docosenoic acid (**8**)	77
1-tridecyne	11-bromoundecanoic acid	(12*Z*)-tetracosenoic acid (**9**)	44
1-tridecyne	13-bromotridecanoic acid	(14*Z*)-hexacosenoic acid (**10**)	53
1-tridecyne	15-bromopentadecanoic acid	(16*Z*)-octacosenoic acid (**11**)	50

We prepared 11 examples ranging in yields from 44 to 95% using reagents as received from the suppliers and solvents dried over molecular sieves only. ^1^H and ^13^C NMR data for all synthesised compounds are available as supplementary data.

### Very-long-chain (VLC) alkene synthesis

While VLC alkenes can be obtained by decarboxylation of VLCFAs^12^, with the increased commercial availability of many alkyltriphenylphosphonium bromides, a Wittig-based approach is an attractive alternative to prepare (*Z*)-configured monoenes in a single step at high purity. In our hands, we prepared 17 different examples in un-optimised yields ranging from 13 to 92% (Table 2). In addition, excellent (*Z*/*E*) selectivity with no trace of (*E*)-isomers detected by ^13^C NMR was obtained by using HMPA as a co-solvent at low temperatures.

**Table 2:** Synthesis details for VLC alkenes. NMR data for numbered compounds are available in SI.

Aldehyde	Alkylphosphonium bromide	VLC alkene	Yield (%)
tetradecanal	heptyltriphenylphosphonium bromide	(7*Z*)-heneicosene (**12**)	41
hexadecanal	heptyltriphenylphosphonium bromide	(7*Z*)-tricosene (**13**)	17
octadecanal	heptyltriphenylphosphonium bromide	(7*Z*)-pentacosene (**14**)	86
heneicosanal	heptyltriphenylphosphonium bromide	(7*Z*)-heptacosene (**15**)	34
docosanal	heptyltriphenylphosphonium bromide	(7*Z*)-nonacosene (**16**)	32*
dodecanal	nonyltriphenylphosphonium bromide	(9Z)-heneicosene (**17**)	92
tetradecanal	nonyltriphenylphosphonium bromide	(9*Z*)-tricosene (**18**)	23
hexadecanal	nonyltriphenylphosphonium bromide	(9*Z*)-pentacosene (**19**)	32
octadecanal	nonyltriphenylphosphonium bromide	(9*Z*)-heptacosene (**20**)	90
heneicosanal	nonyltriphenylphosphonium bromide	(9*Z*)-nonacosene (**21**)	13
heptanal	dodecyltriphenylphosphonium bromide	(12*Z*)-nonadecene (**22**)	32
nonanal	dodecyltriphenylphosphonium bromide	(12*Z*)-heneicosene (**23**)	32
undecanal	dodecyltriphenylphosphonium bromide	(12*Z*)-tricosene (**24**)	22
tridecanal	dodecyltriphenylphosphonium bromide	(12*Z*)-pentacosene (**25**)	23
pentadecanal	dodecyltriphenylphosphonium bromide	(12*Z*)-heptacosene (**26**)	34
heptadecanal	dodecyltriphenylphosphonium bromide	(12*Z*)-nonacosene (**27**)	32
nonadecanal	dodecyltriphenylphosphonium bromide	(12*Z*)-hentriacosene (**28**)	33

*Dimer from Wittig reagent formed as side product, not separated by MPLC.

## Discussion

Fatty acids and their lipid derivatives play important roles in many interactions between plants and other organisms, such as pathogens and pollinators^24^. In order to produce chemical cues for pollinator attraction in sexually deceptive orchids, fatty acids are needed as precursors; for instance for alkanes and alkenes, but also aldehydes and more specialised metabolites that likely derive from the fatty acid pathway, such as chiloglottones^9^. While synthetic alkenes in high purity are required to study the chemical ecology of plant-pollinator interactions in sexually deceptive orchids such as *Ophrys* by enabling behavioural experiments and bioassays with insects, their biosynthetic precursors, particularly VLCFAs, are necessary to enable studies on the biochemistry and evolution of pollinator-attractive hydrocarbons. For instance, chain-length differences in alkenes have been observed between different *Ophrys* species with different pollinators^25^. Without the availability of the relevant unsaturated VLCFAs, it is impossible to experimentally test hypotheses on the biosynthetic processes producing alkenes of different chain length (e.g. by testing the substrate specificity of different elongase enzymes^25^).

There are several methods available to prepare VLCFAs such as coupling of alk(en)yl bromides and ω-bromocarboxylic acids via Grignard reactions in the presence of Cu(I)-salts or Li_2_CuCl_4_^26,27^, or Wurtz type couplings using Ni-salts in the presence of manganese^28^. Very recently, an acyl chloride Negishi coupling was reported to yield grams of VLCFAs^29^. For our purposes, these methods were found suboptimal, either due to labour-intensive steps, sensitive methodology or non-standard reagents, or unsatisfactory purity of the final products. For example, although the preparation and reactions of long-chained Grignard reagents have been reported^11^, they are known to be difficult (B. Bohman, pers. obs.) and in our hands we struggled to obtain acceptable, if any, yields with this methodology. Wurtz-couplings, instead have been shown to give homo-couplings and mixtures of products^28^. The corresponding VLC alkene oxidation products can be formed from the prepared VLCFAs, alternatively by Wittig-type reactions between phosphonium salts of alkylbromides and aldehydes. A key general limitation to Wittig reactions is the unavoidable formation of (*E*)-alkenes alongside the target (*Z*)-alkenes, in addition to homo-coupling products from the phosphonium salts, unless the reaction conditions and solvents are carefully chosen^11^.

By combining two lesser-known methods from the literature^11,21^, we managed to prepare 11 VLCFAs in only two steps and one purification step with column chromatography. In addition, we also prepared 17 alkenes in a single step, making use of a Wittig reaction methodology, all using commercially available reactants and reagents. It is noteworthy that all VLC alkenes could be synthesised in > 97% isomeric purity. The only shortcoming in terms of product purity is the possibility for homo-couplings between certain Wittig-reagents, even with our modified methods, yielding product mixtures that are difficult to separate. Among our 17 alkenes, this was however only a problem for the preparation of a single compound, (7*Z*)-nonacosene (Table 2). We did not attempt to optimise yields in this study, and each reaction was only completed once, to obtain our required products.

In conclusion, the two methods presented here are simple, time-effective approaches to prepare VLCFAs and VLC alkenes, which can be implemented without any special equipment or sensitive reagents. Such practical, general and accessible synthetic methods are essential for chemical ecology research to aid researchers in unambiguously identifying isolated semiochemicals and their precursors.

### Supplementary Information

Supplementary Information.

## Data Availability

The original contributions presented in the study are included in the article/supplementary material, further inquiries can be directed to the corresponding author.
