# Supplementary material for: Practical preparation of unsaturated very-long-chain fatty acids (VLCFAs) and very-long-chain alkene pollinator attractants
Source: Sci Rep. 2024 Aug 24;14:19694. doi: 10.1038/s41598-024-70598-x (PMC11344852; doi:10.1038/s41598-024-70598-x)
Supplement: Supplementary file 1 — Supplementary Information. [file 41598_2024_70598_MOESM1_ESM.docx]

Supplementary Information

**Practical preparation of unsaturated very-long-chain fatty acids (VLCFAs) and very-long-chain alkene pollinator attractants**

Björn Bohman, Aylin J Bersch, Gavin R Flematti and Philipp M Schlüter

**Representative example of VLCFA synthesis: (*Z*)-14-hexacosenoic acid (10)**

*n*-BuLi (3.0 mL, 1.6 M in hexanes) was added to 1-tridecyne (0.85 g, 4,7 mmol) in HMPA (5 mL) at 0 °C. After 15 min, 13-bromotridecanoic acid (460 mg, 1.57 mmol) in HMPA (4 mL) was added dropwise at the same temperature. The reaction mixture was stirred at room temperature over night. The red-brown solution was quenched with ice-cold HCl (1 M), followed by acidification with HCl (6 M), then extracted three times with diethyl ether, washed twice with HCl (1 M), twice with water and twice with brine, dried over magnesium sulphate and concentrated *in vacuo* to a crude yellow powder that after MPLC (gradient from hexane to ethyl acetate) gave 387 mg (63%) of a white powder. This intermediate was hydrogenated at room temperature with Lindlar catalyst (100 mg) and H_2_ (1 Atm) in ethyl acetate (100 mL). The final product was obtained as a white powder (328 mg, 85%).

**Representative example of VLC alkene synthesis: (*Z*)-9-heneicosene (17)**

Nonyltriphenylphosphoniumbromide (2.5 g) was dissolved in THF/HMPA (4:1, 10 mL) and cooled on an ice-bath. LiHMDS (1.0 M in THF, 5.3 mL) was added and after 10 min the mixture was cooled on dry ice/acetone and dodecanal (552 mg, 3.0 mmol) was added and the reaction mixture was stirred at -78°C for 1 hour. Saturated ammonium chloride was added and the product was extracted three times with ethyl acetate, washed with water and brine, dried over magnesium sulphate, concentrated *in vacuo* and purified with MPLC (hexanes) to give a colourless oil (813 mg, 92%).

**NMR spectroscopy**

^1^H and ^13^C NMR spectra were recorded at 298 K, at 400 and 100 MHz, respectively, on a Bruker Avance 400 spectrometer. Chemical shifts are reported in ppm. NMR experiments were run in CDCl_3_ and are referenced to the resonance from residual CHCl_3_ at 7.26 ppm for ^1^H and to the central peak in the signal from CDCl_3_ at 77.16 ppm for ^13^C. The appearance and multiplicities of ^1^H resonances are expressed by the following abbreviations: br (broad), t (triplet), m (multiplet), and combinations thereof. ^1^H and ^13^C NMR signals where appropriate are described by chemical shift (multiplicity, |J| (Hz), integration).

**Compound 1:**

^1^H: 5.34 (br t, 4.7 Hz, 2H), 2.34 (t, 7.6 Hz, 2H), 2.02 (m, 4H), 1.63 (m, 2H), 1.23-1.38 (m, 24H), 0.88 (t, 7.1 Hz, 3H).

^13^C: 179.58, 129.87, 129.85, 33.95, 31.75, 29.73, 29.71, 29.56, 29.54, 29.50, 29.37, 29.27, 29.20, 29.03, 28.95, 27.18, 27.17, 24.66, 22.62, 14.06.

**Compound 2:**

^1^H: 5.35 (br t, 4.7 Hz, 2H), 2.35 (t, 7.4 Hz, 2H), 2.02 (m, 4H), 1.63 (m, 2H), 1.23-1.38 (m, 26H), 0.88 (t, 7.0 Hz, 3H).

^13^C: 179.68, 2 x 129.89, 33.98, 31.80, 29.77, 29.74, 29.64, 2 x 29.63, 29.58, 29.55, 29.43, 29.31, 29.24, 29.06, 28.98, 27.21, 27.20, 24.68, 22.65, 14.09.

**Compound 3:**

^1^H: 5.35 (br t, 4.7 Hz, 2H), 2.35 (t, 7.6 Hz, 2H), 2.02 (m, 4H), 1.63 (m, 2H), 1.23-1.38 (m, 28H), 0.88 (t, 7.1 Hz, 3H).

^13^C: 179.22, 129.90, 129.89, 33.90, 31.59, 29.77, 29.74, 4 x 29.65-29.68, 29.59, 29.56, 29.43, 29.31, 29.24, 29.18, 29.06, 28.99, 2 x 27.21, 24.69, 22.65, 14.11.

**Compound 4:**

^1^H: 5.35 (br t, 4.8 Hz, 2H), 2.35 (t, 7.4 Hz, 2H), 2.02 (m, 4H), 1.63 (m, 2H), 1.23-1.37 (m, 30H), 0.89 (t, 7.0 Hz, 3H).

^13^C: 179.49, 129.90, 129.89, 33.94, 31.78, 29.77, 29.74, 7 x 29.65-29.69, 29.59, 29.56, 29.44, 29.31, 29.24, 29.06, 28.99, 27.22, 27.21, 24.68, 22.66, 14.10.

**Compound 5:**

^1^H: 5.35 (br t, 4.8 Hz, 2H), 2.35 (t, 7.6 Hz, 2H), 2.01 (m, 4H), 1.63 (m, 2H), 1.23-1.36 (m, 32H), 0.88 (t, 7.1 Hz, 3H).

^13^C: 178.68, 129.91, 129.89, 33.80, 31.78, 29.77, 29.74, 9 x 29.65-29.69, 29.59, 29.56, 29.43, 29.31, 29.24, 29.06, 28.99, 27.21, 27.20, 24.69, 22.65, 14.10.

**Compound 6:**

^1^H: 5.35 (br t, 4.8 Hz, 2H), 2.35 (t, 7.4 Hz, 2H), 2.01 (m, 4H), 1.63 (m, 2H), 1.23-1.38 (m, 30H), 0.88 (t, 7.1 Hz, 3H).

^13^C: 179.39, 2 x 129.89, 33.92, 31.90, 2 x 29.77, 7 x 29.65-29.68, 29.59, 29.56, 29.52, 2 x 29.31, 29.24, 29.06, 2 x 27.20, 24.68, 22.68, 14.10.

**Compound 7:**

^1^H: 5.35 (br t, 4.8 Hz, 2H), 2.35 (t, 7.6 Hz, 2H), 2.01 (m, 4H), 1.63 (m, 2H), 1.23-1.37 (m, 32H), 0.88 (t, 7.0 Hz, 3H).

^13^C: 179.31, 2 x 129.90, 33.90, 31.91, 2 x 29.77, 8 x 29.64-29.69, 29.59, 29.56, 29.52, 29.43, 2 x 29.32, 29.24, 29.06, 2 x 27.21, 24.68, 22.68, 14.10.

**Compound 8:**

^1^H: 5.34 (br t, 5.0 Hz, 2H), 2.34 (t, 7.6 Hz, 2H), 2.01 (m, 4H), 1.63 (m, 2H), 1.23-1.37 (m, 26H), 0.88 (t, 7.1 Hz, 3H).

^13^C: 179.50, 129.97, 129.78, 33.95, 31.92, 29.76, 29.72, 2 x 29.68, 2 x 29.65, 29.56, 2 x 29.31, 29.22, 29.21, 29.05, 27.21, 27.18, 24.67, 22.68, 14.11.

**Compound 9:**

^1^H: 5.35 (br t, 4.7 Hz, 2H), 2.35 (t, 7.4 Hz, 2H), 2.01 (m, 4H), 1.63 (m, 2H), 1.23-1.37 (m, 26H), 0.88 (t, 7.0 Hz, 3H).

^13^C: 179.30, 129.92, 129.86, 33.69, 31.92, 2 x 29.76, 29.68, 2 x 29.65, 29.56, 29.52, 29.42, 29.35, 29.31, 29.29, 2 x 29.23, 29.06, 2 x 27.20, 24.70, 22.69, 14.11.

**Compound 10:**

^1^H: 5.35 (br t, 4.7 Hz, 2H), 2.35 (t, 7.6 Hz, 2H), 2.01 (m, 4H), 1.63 (m, 2H), 1.23-1.37 (m, 28H), 0.88 (t, 7.1 Hz, 3H).

^13^C: 179.17, 129.90, 129.89, 33.89, 31.92, 2 x 29.77, 8 x 29.68-29.56, 29.43, 29.36, 2 x 29.31, 29.24, 29.06, 2 x 27.21, 24.69, 22.69, 14.11.

**Compound 11:**

^1^H: 5.35 (br t, 4.8 Hz, 2H), 2.35 (t, 7.6 Hz, 2H), 2.01 (m, 4H), 1.63 (m, 2H), 1.22-1.38 (m, 30H), 0.88 (t, 7.1 Hz, 3H).

^13^C: 179.29, 2 x 129.90, 33.91, 31.92, 2 x 29.77, 10 x 29.68-29.56, 29.44, 29.35, 2 x 29.31, 29.24, 29.06, 2 x 27.20, 24.68, 22.69, 14.11.

**Compound 12:**

^1^H: 5.35 (br t, 4.6 Hz, 2H), 2.01 (m, 4H), 1.22-1.38 (m, 30H), 0.88 (br t, 7.0 Hz, 6H).

^13^C: 129.90, 129.89, 31.92, 31.78, 29.77, 29.74, 5 x 29.65-29.69, 29.55, 29.36, 29.31, 28.98, 27.21, 27.20, 22.68, 22.65, 14.10, 14.09.

**Compound 13:**

^1^H: 5.35 (br t, 4.8 Hz, 2H), 2.01 (m, 4H), 1.22-1.38 (m, 32H), 0.88 (br t, 7.1 Hz, 6H).

^13^C: 129.91, 129.89, 31.92, 31.78, 29.77, 29.74, 7 x 29.65-29.69, 29.56, 29.36, 29.31, 28.99, 2 x 27.22, 22.69, 22.66, 14.11, 14.09.

**Compound 14:**

^1^H: 5.35 (br t, 4.7 Hz, 2H), 2.01 (m, 4H), 1.23-1.36 (m, 34H), 0.88 (br t, 7.2 Hz, 6H).

^13^C: 129.91, 129.89, 31.93, 31.79, 29.77, 29.74, 9 x 29.65-29.69, 29.56, 29.36, 29.31, 28.99, 27.22, 27.21, 22.69, 22.66, 14.11, 14.10.

**Compound 15:**

^1^H: 5.35 (br t, 4.7 Hz, 2H), 2.01 (m, 4H), 1.24-1.36 (m, 36H), 0.89 (t, 7.1 Hz, 3H), 0.88 (t, 7.1 Hz, 3H).

^13^C: 129.90, 129.89, 31.92, 31.78, 29.77, 29.74, 11 x 29.65-29.69, 29.55, 29.36, 29.31, 28.99, 2 x 27.21, 22.69, 22.65, 14.11, 14.09.

**Compound 16:**

^1^H: 5.35 (br t, 4.8 Hz, 2H), 2.02 (m, 4H), 1.24-1.36 (m, 38H), 0.89 (t, 7.1 Hz, 3H), 0.88 (t, 7.1 Hz, 3H).

^13^C: 2 x 129.89, 31.93, 31.79, 29.77, 29.74, 13 x 29.65-29.70, 29.56, 29.36, 29.31, 28.99, 2 x 27.21, 22.69, 22.65, 14.11, 14.09.

**Compound 17:**

^1^H: 5.35 (br t, 4.8 Hz, 2H), 2.02 (m, 4H), 1.24-1.36 (m, 30H), 0.88 (br t, 7.1 Hz, 6H).

^13^C: 2 x 129.90, 31.93, 31.91, 2 x 29.78, 29.69, 2 x 29.66, 29.57, 29.53, 29.36, 3 x 29.32, 2 x 27.21, 2 x 22.69, 2 x 14.11.

**Compound 18:**

^1^H: 5.35 (br t, 4.9 Hz, 2H), 2.01 (m, 4H), 1.24-1.36 (m, 32H), 0.88 (br t, 7.1 Hz, 6H).

^13^C: 2 x 129.90, 31.92, 31.91, 2 x 29.77, 3 x 29.69, 2 x 29.65, 29.56, 29.52, 29.36, 3 x 29.32, 2 x 27.20, 2 x 22.68, 2 x 14.10.

**Compound 19:**

^1^H: 5.35 (br t, 4.7 Hz, 2H), 2.02 (m, 4H), 1.24-1.36 (m, 34H), 0.88 (br t, 7.2 Hz, 6H).

^13^C: 2 x 129.90, 31.92, 31.91, 2 x 29.77, 5 x 29.69, 2 x 29.66, 29.56, 29.52, 29.36, 3 x 29.32, 2 x 27.21, 2 x 22.68, 2 x 14.10.

**Compound 20:**

^1^H: 5.35 (br t, 4.8 Hz, 2H), 2.02 (m, 4H), 1.24-1.35 (m, 36H), 0.88 (br t, 7.1 Hz, 6H).

^13^C: 2 x 129.90, 31.93, 31.91, 2 x 29.78, 7 x 29.69, 2 x 29.66, 29.57, 29.53, 29.36, 3 x 29.32, 2 x 27.21, 2 x 22.69, 2 x 14.11.

**Compound 21:**

^1^H: 5.35 (br t, 4.7 Hz, 2H), 2.02 (m, 4H), 1.24-1.35 (m, 38H), 0.88 (br t, 7.0 Hz, 6H).

^13^C: 2 x 129.90, 31.92, 31.91, 2 x 29.77, 9 x 29.69, 2 x 29.65, 29.56, 29.52, 29.36, 3 x 29.32, 2 x 27.20, 2 x 22.68, 2 x 14.11.

**Compound 22:**

^1^H: 5.35 (br t, 4.7 Hz, 2H), 2.02 (m, 4H), 1.24-1.37 (m, 28H), 0.88 (br t, 7.1 Hz, 6H).

^13^C: 2 x 129.90, 31.92, 31.91, 29.78, 29.69, 2 x 29.65, 29.56, 29.52, 29.36, 2 x 29.32, 2 x 27.22, 2 x 22.69, 2 x 14.11.

**Compound 23:**

^1^H: 5.35 (br t, 4.8 Hz, 2H), 2.02 (m, 4H), 1.23-1.37 (m, 30H), 0.89 (t, 7.0 Hz, 3H), 0.88 (t, 7.0 Hz, 3H).

^13^C: 2 x 129.90, 2 x 31.92, 29.78, 29.75, 5 x 29.65-29.69, 29.56, 29.52, 29.35, 29.31, 2 x 27.22, 22.69, 22.66,14.11, 14.09.

**Compound 24:**

^1^H: 5.35 (br t, 4.6 Hz, 2H), 2.01 (m, 4H), 1.23-1.37 (m, 32H), 0.88 (br t, 7.0 Hz, 6H).

^13^C: 2 x 129.90, 2 x 31.92, 2 x 29.78, 29.69, 4 x 29.66, 2 x 29.56, 2 x 29.36, 2 x 29.32, 2 x 27.21, 2 x 22.69, 2 x 14.11.

**Compound 25:**

^1^H: 5.35 (br t, 4.7 Hz, 2H), 2.01 (m, 4H), 1.23-1.37 (m, 34H), 0.88 (br t, 7.1 Hz, 6H).

^13^C: 2 x 129.90, 2 x 31.93, 2 x 29.78, 2 x 29.69, 5 x 29.66, 2 x 29.56, 2 x 29.36, 2 x 29.32, 2 x 27.21, 2 x 22.69, 2 x 14.11.

**Compound 26:**

^1^H: 5.35 (br t, 4.9 Hz, 2H), 2.02 (m, 4H), 1.23-1.36 (m, 36H), 0.89 (t, 6.9 Hz, 3H), 0.88 (t, 6.9 Hz, 3H).

^13^C: 2 x 129.90, 2 x 31.93, 2 x 29.78, 9 x 29.66-29.69, 2 x 29.56, 2 x 29.36, 2 x 29.32, 2 x 27.21, 2 x 22.69, 2 x 14.11.

**Compound 27:**

^1^H: 5.35 (br t, 4.7 Hz, 2H), 2.01 (m, 4H), 1.24-1.36 (m, 38H), 0.89 (t, 6.9 Hz, 3H), 0.88 (t, 7.1 Hz, 3H).

^13^C: 2 x 129.90, 2 x 31.92, 2 x 29.77, 11 x 29.66-29.69, 2 x 29.56, 2 x 29.35, 2 x 29.31, 2 x 27.20, 2 x 22.69, 2 x 14.11.

**Compound 28:**

^1^H: 5.35 (br t, 4.8 Hz, 2H), 2.02 (m, 4H), 1.24-1.36 (m, 40H), 0.88 (br t, 7.0 Hz, 6H).

^13^C: 2 x 129.90, 2 x 31.93, 2 x 29.77, 13 x 29.65-29.70, 2 x 29.56, 2 x 29.36, 2 x 29.32, 2 x 27.20, 2 x 22.69, 2 x 14.11.

**NMR-spectra**

**Examples of ^1^H-NMR spectra with expansions of key signals**

1. (*Z*)-14-Hexacosenoic acid (**10**)

**
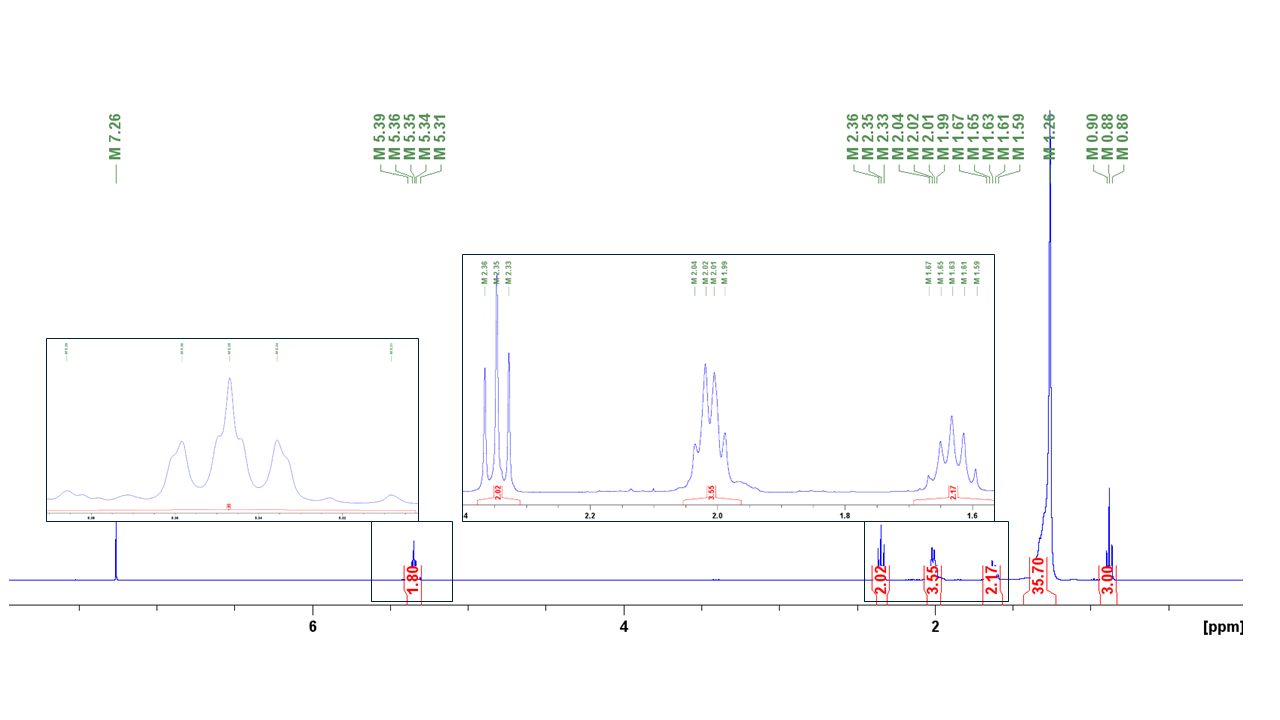
**

1. (*Z*)-9-Heneicosene (**17**)

**
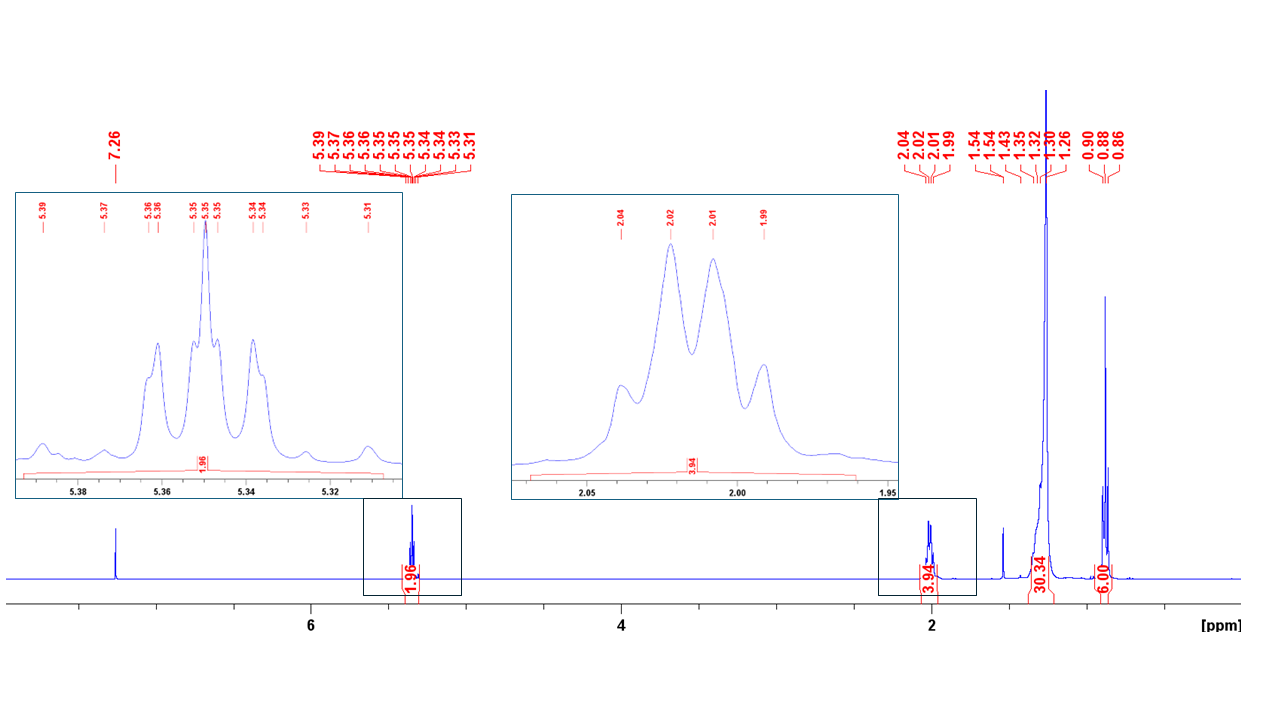
**

**^1^H and ^13^C-NMR spectra of compounds 1-28**

**Compound 1**

**
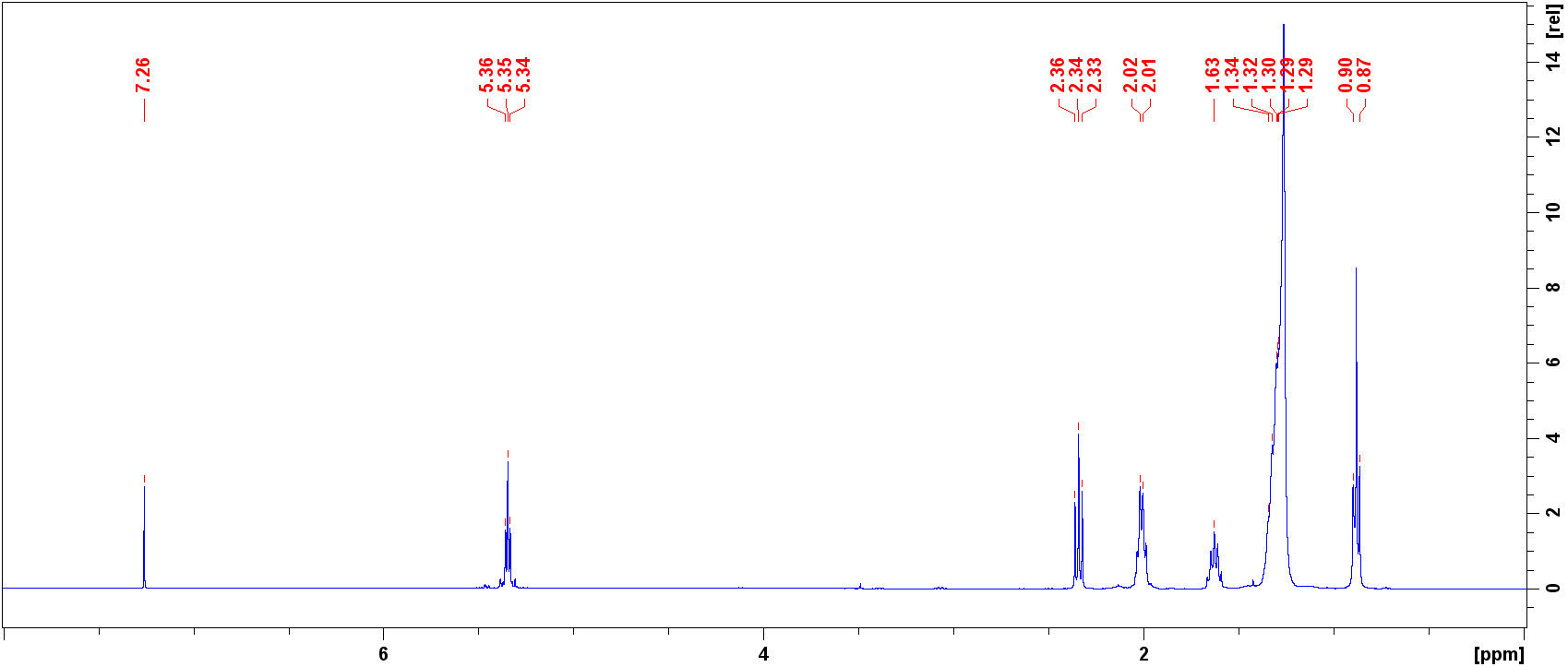
**


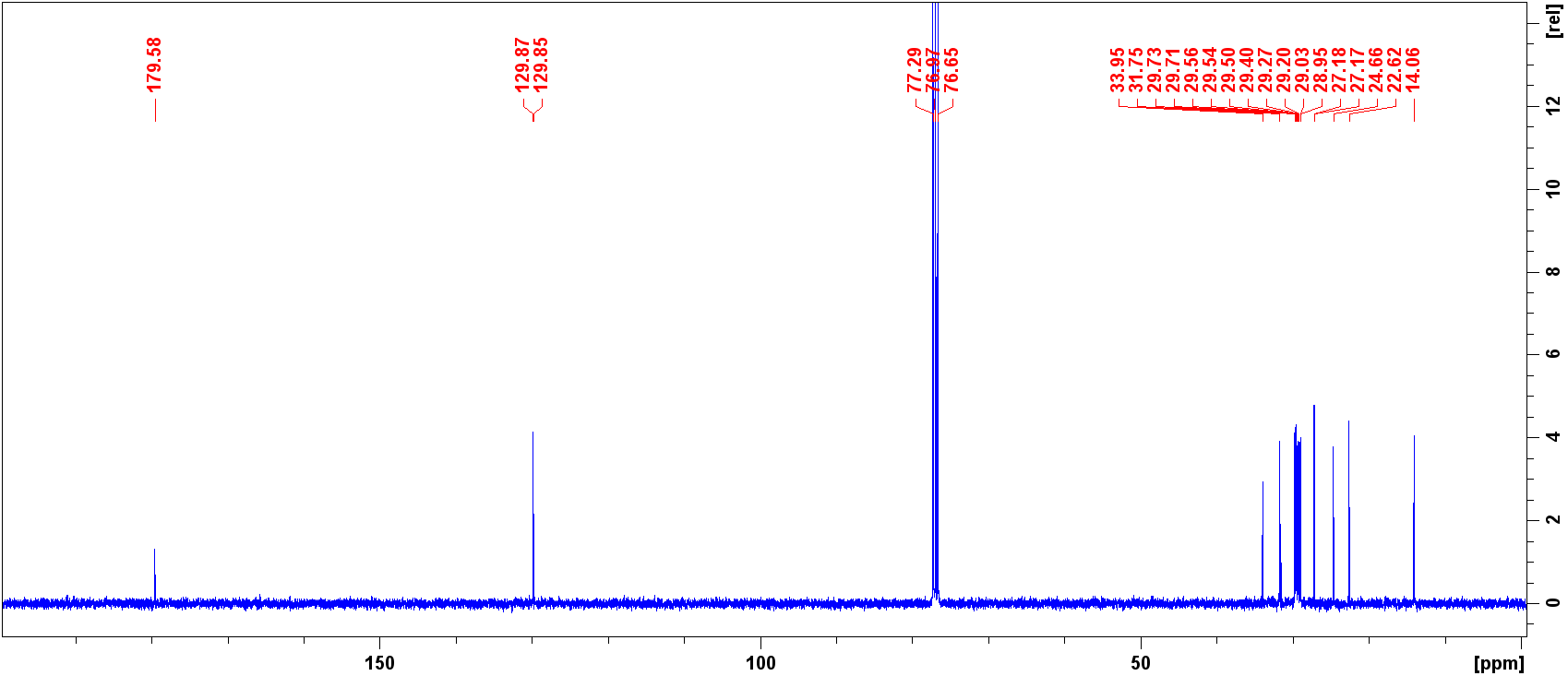


**Compound 2**

**
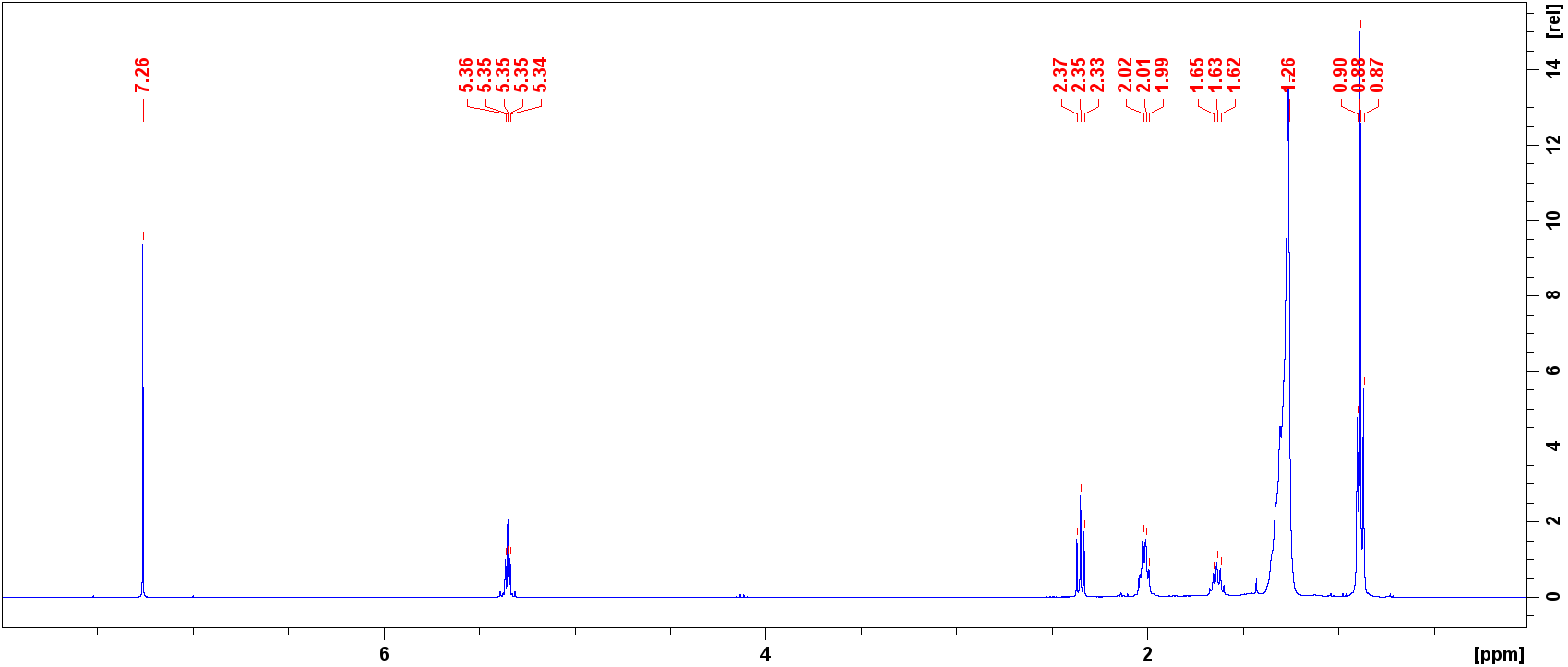
**


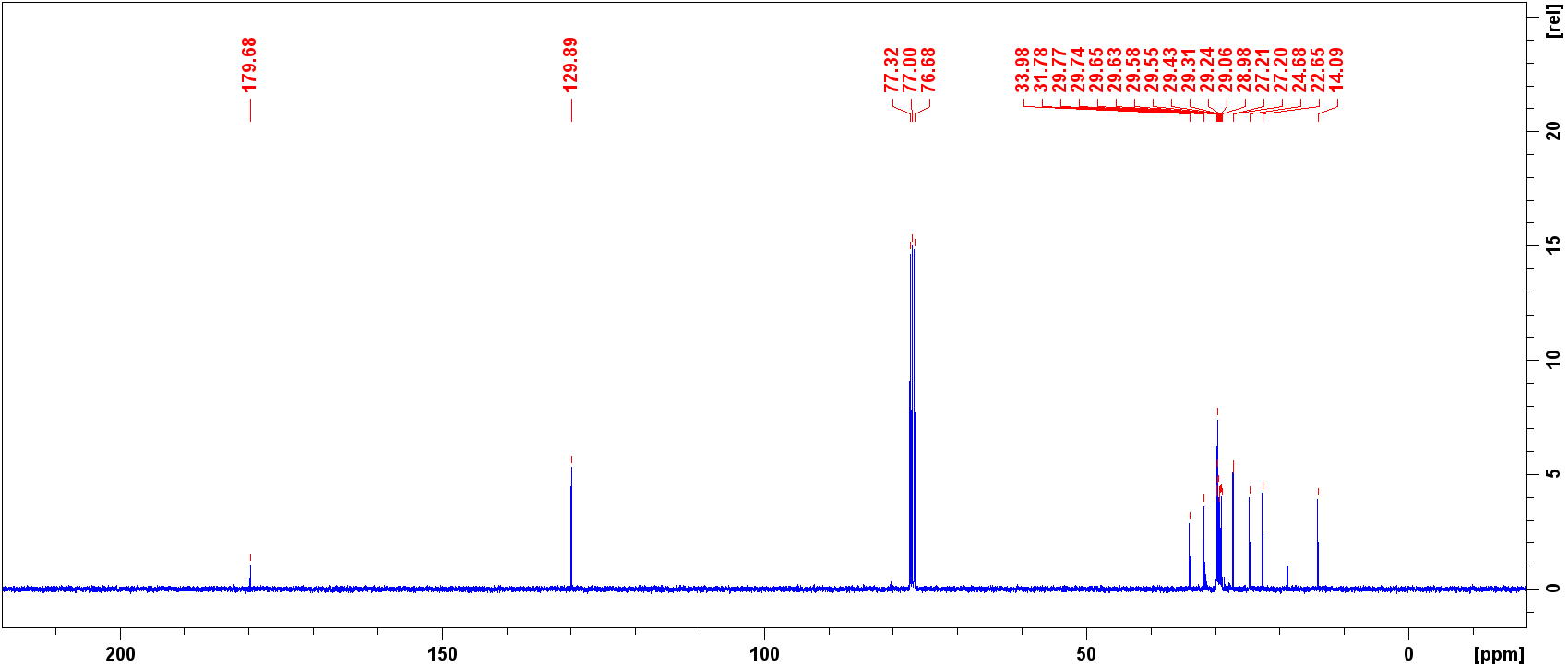


**Compound 3**

**
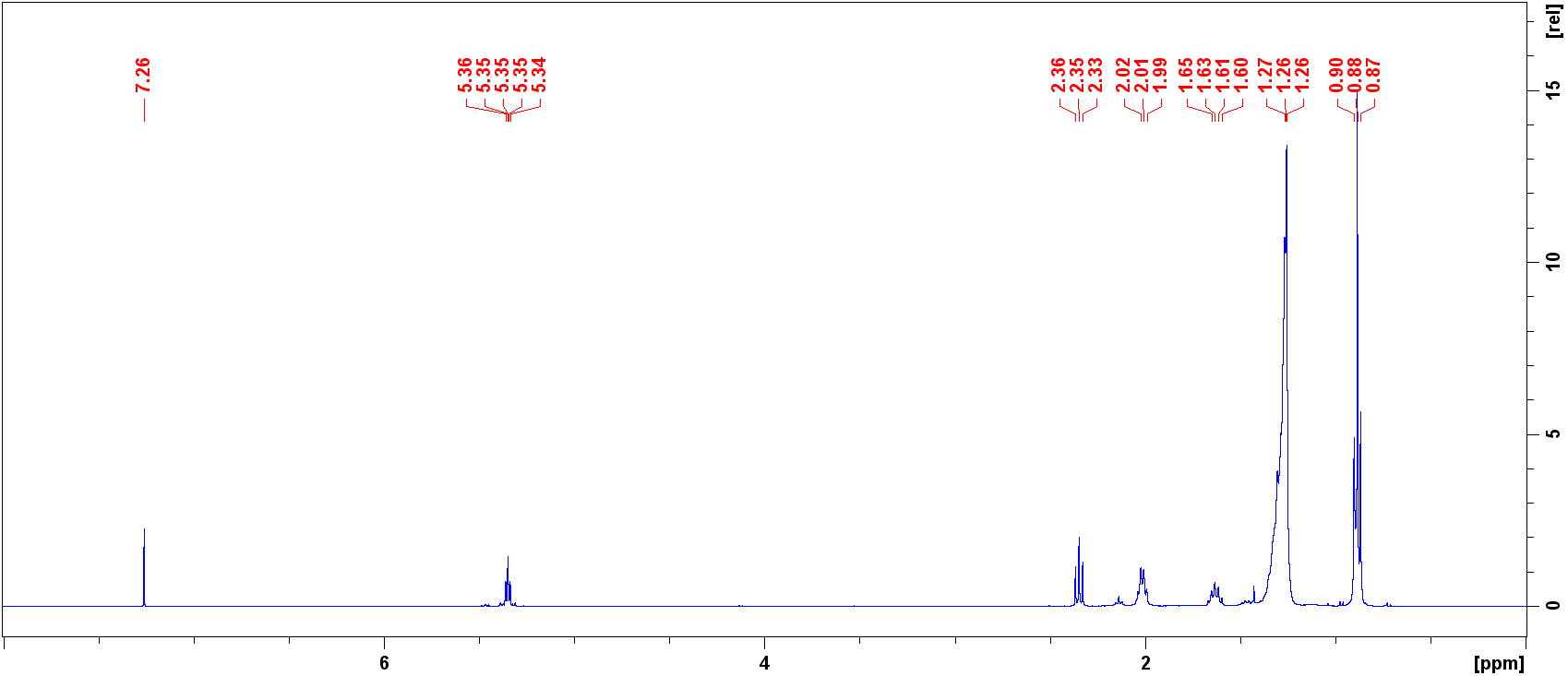
**


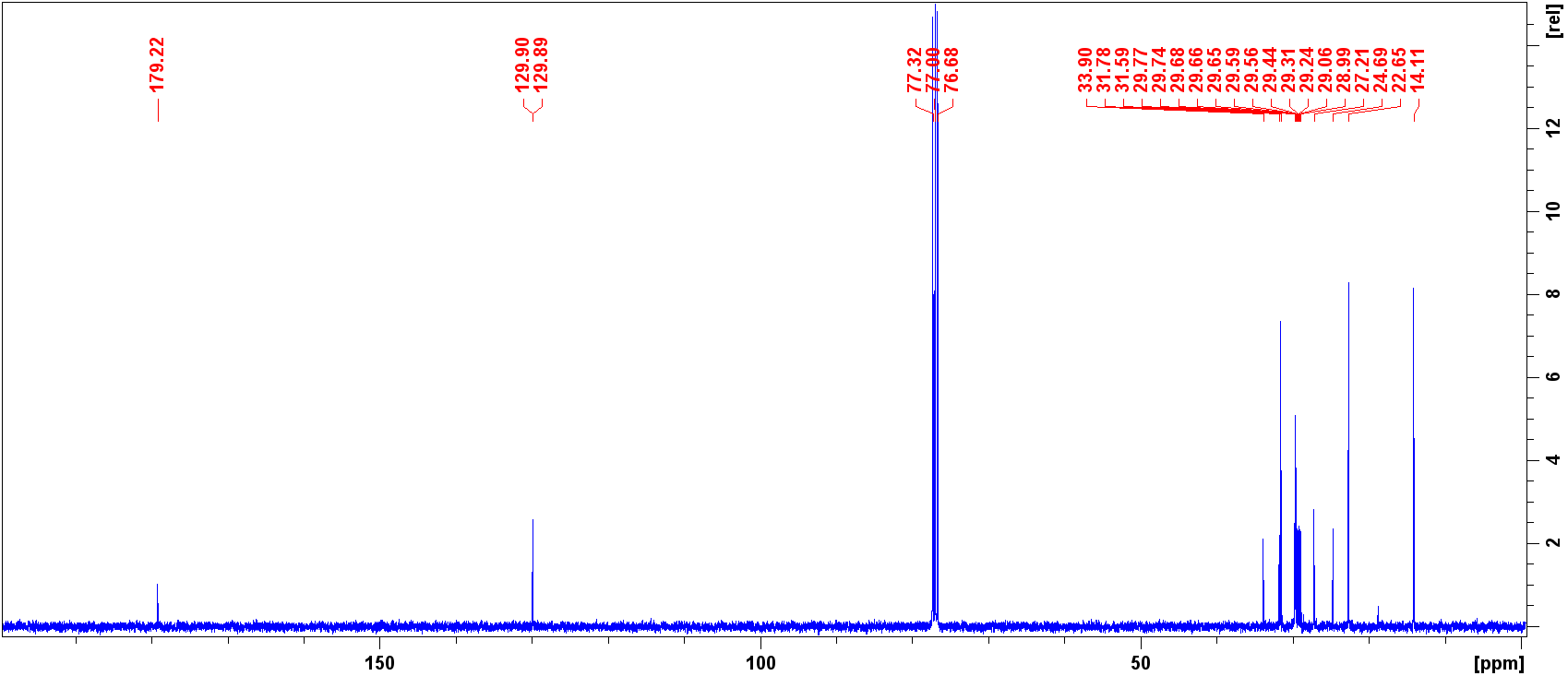


**Compound 4**


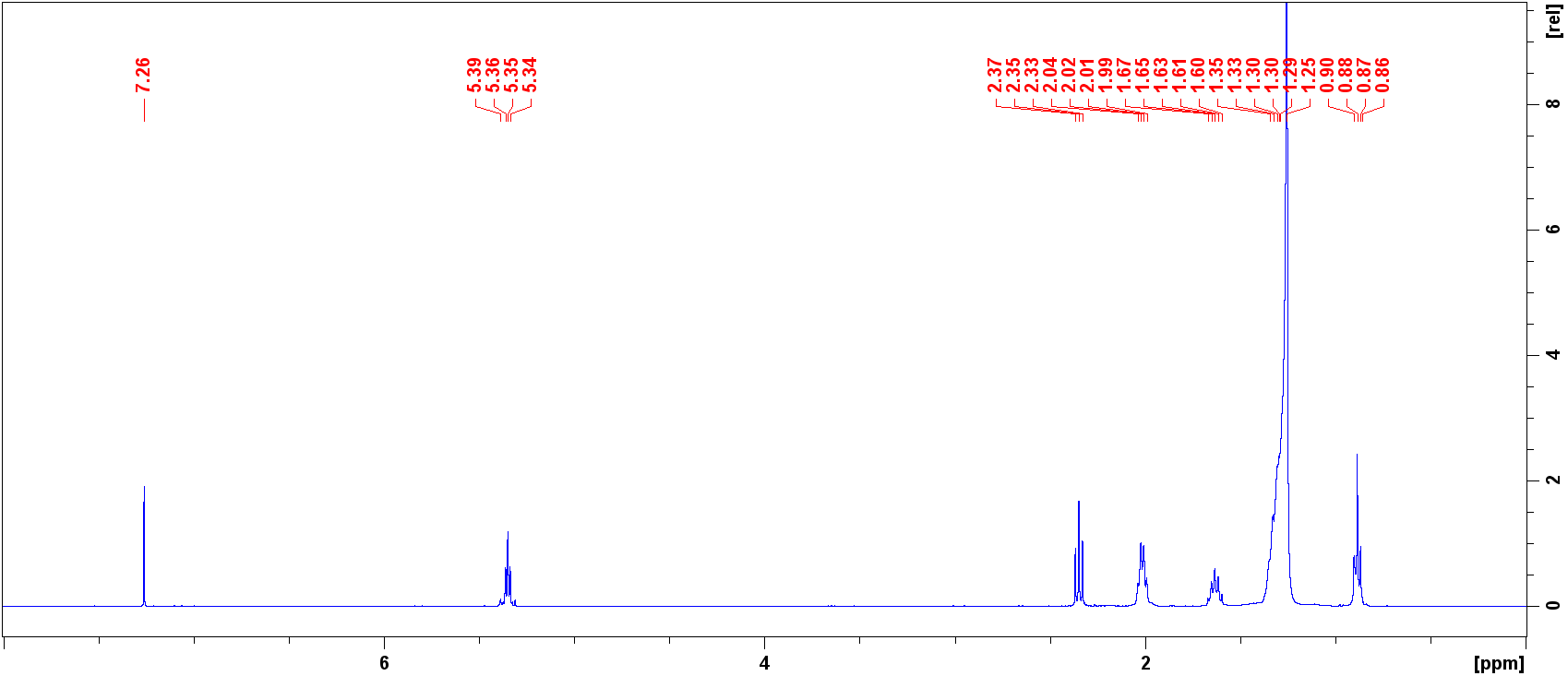


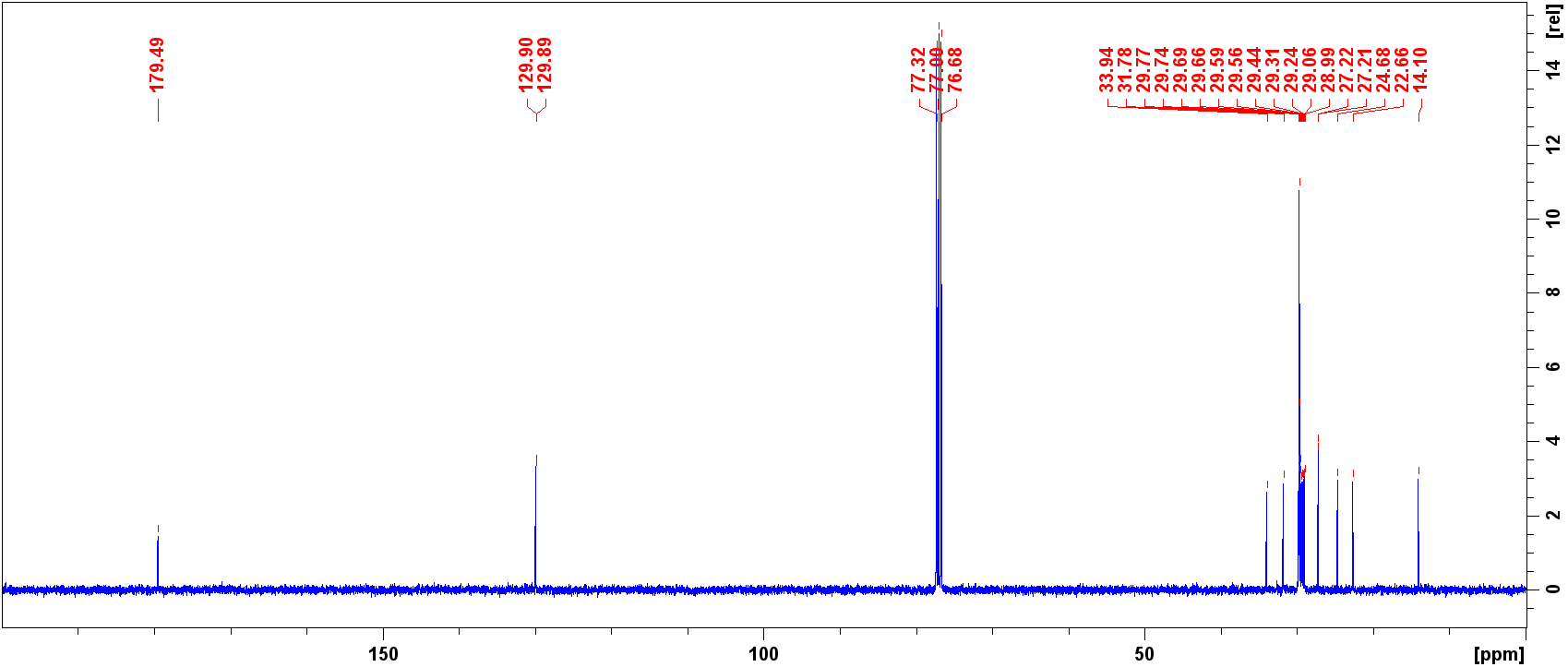


**Compound 5**

**
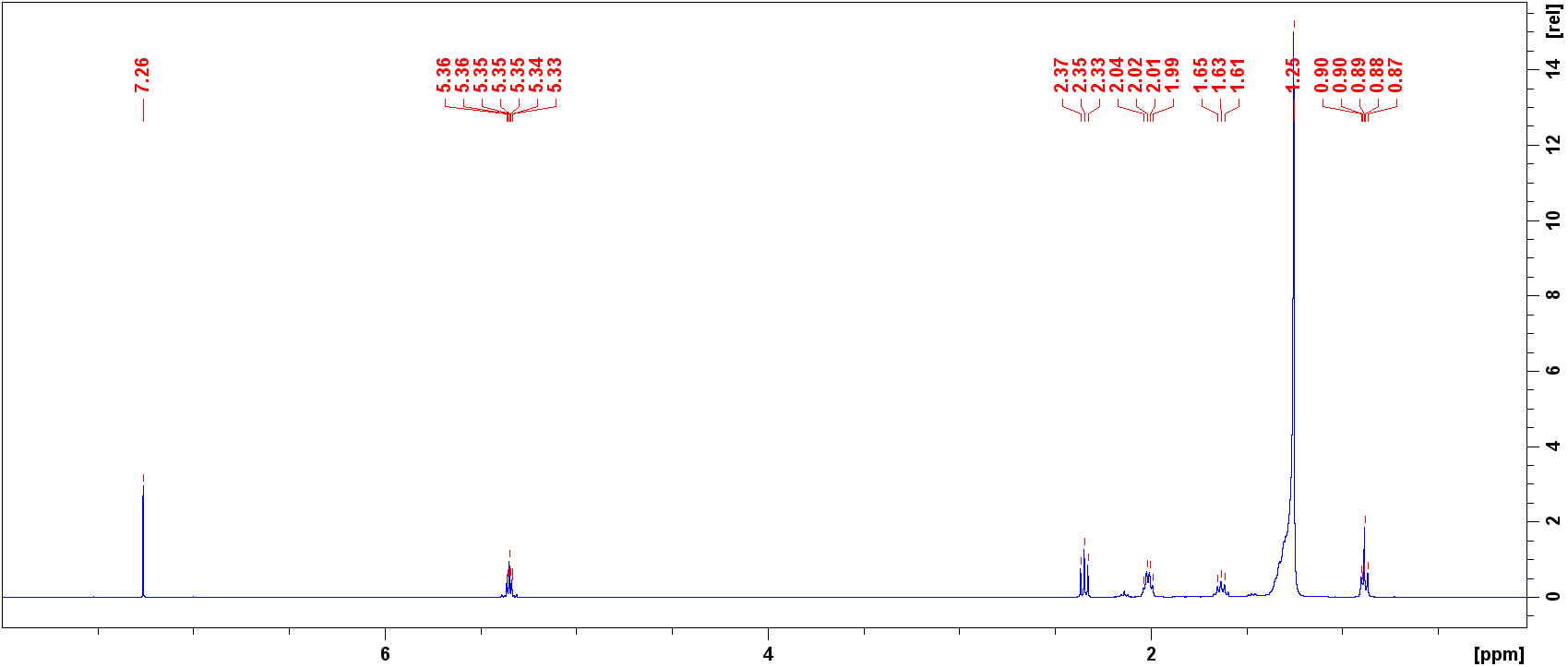
**


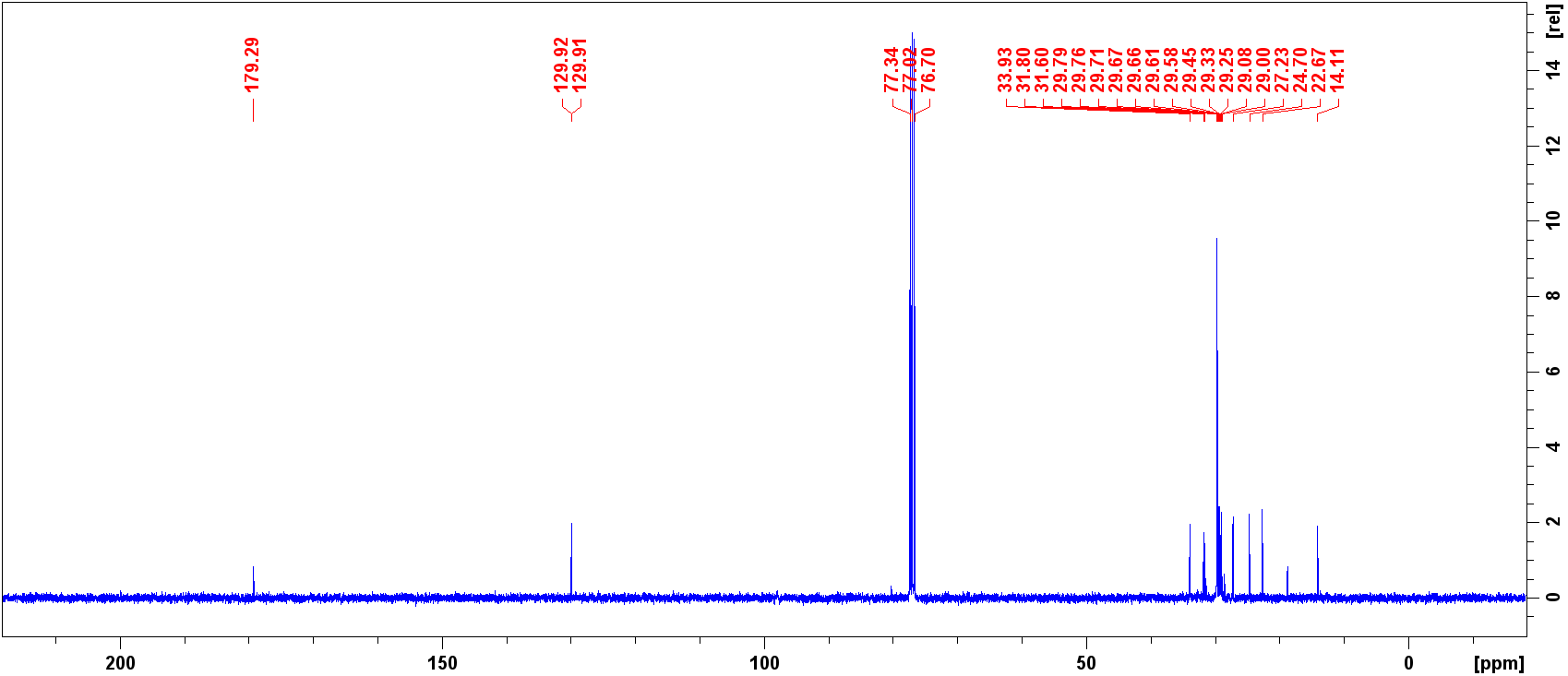


**Compound 6**

**
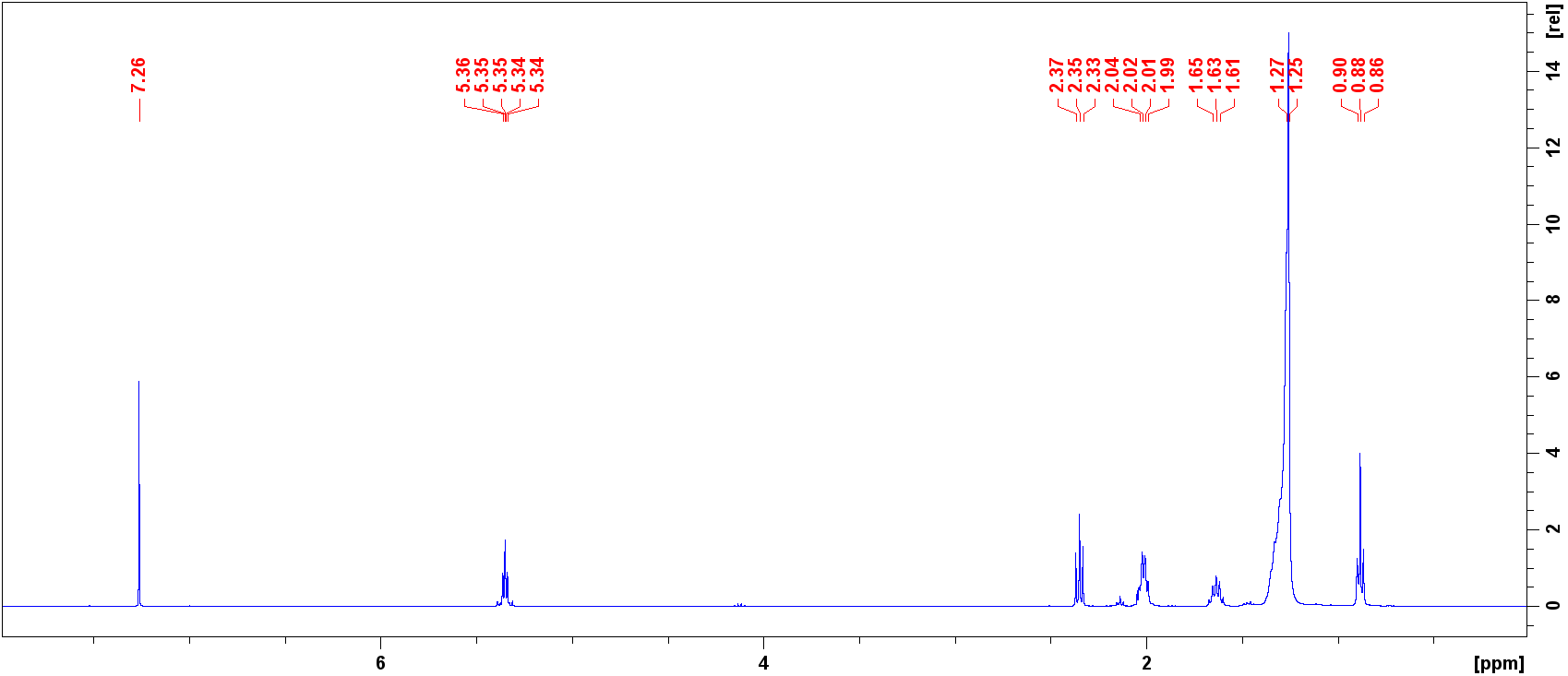
**

**
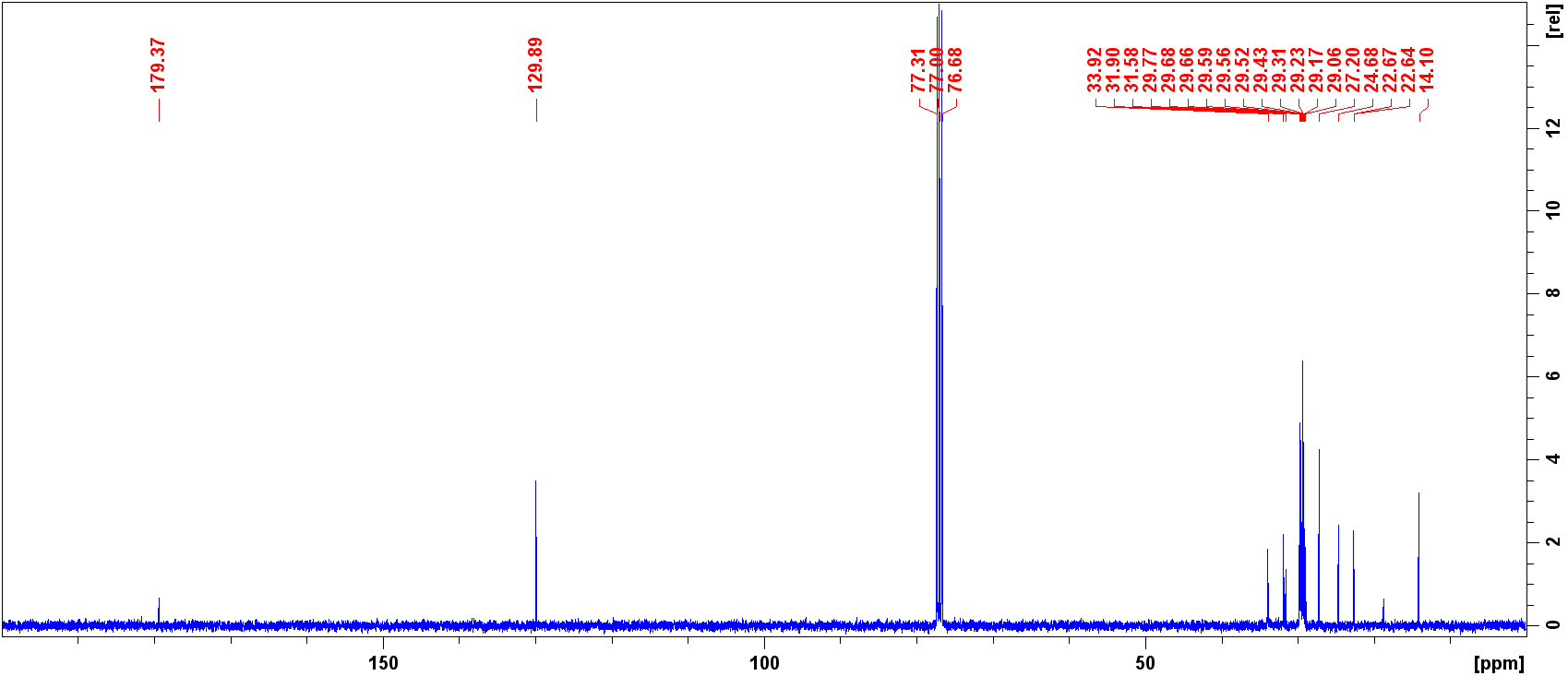
**

**Compound 7**

**
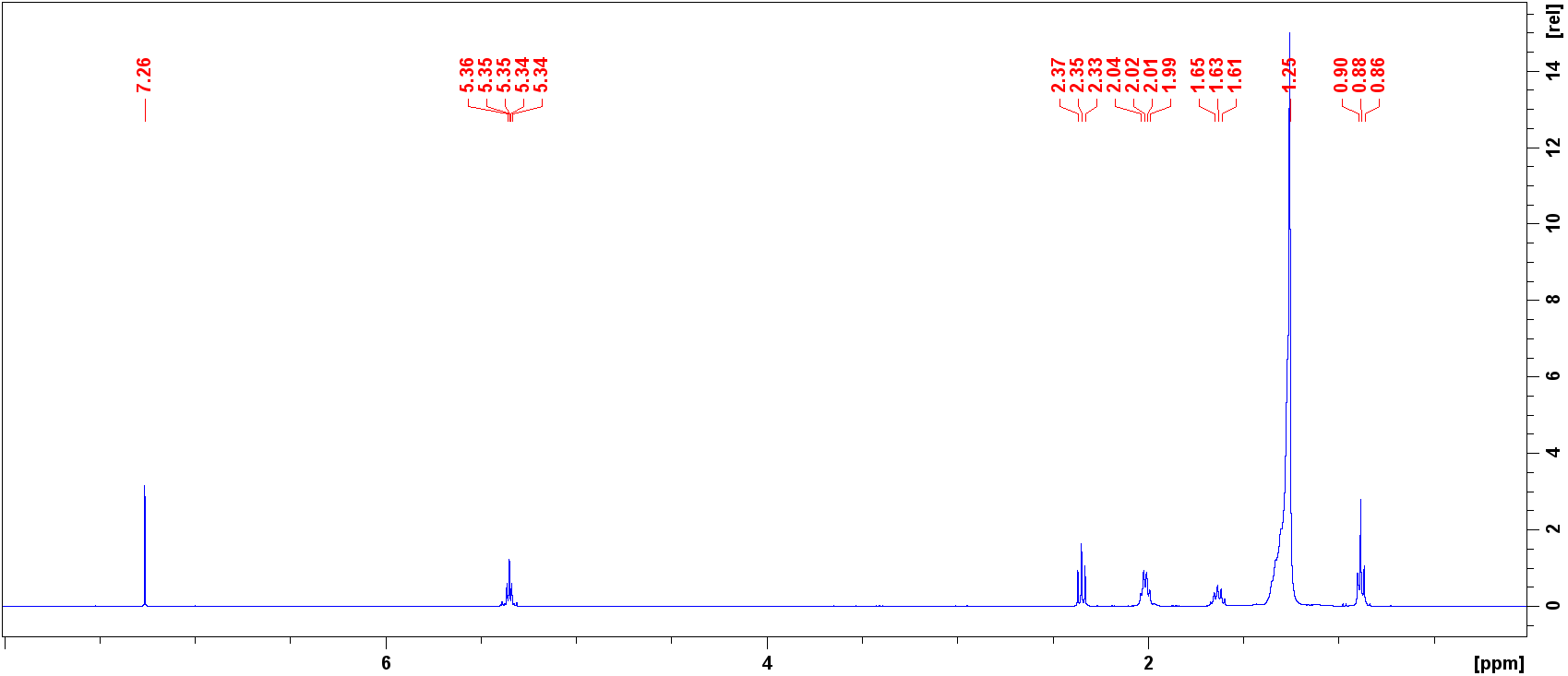
**

**
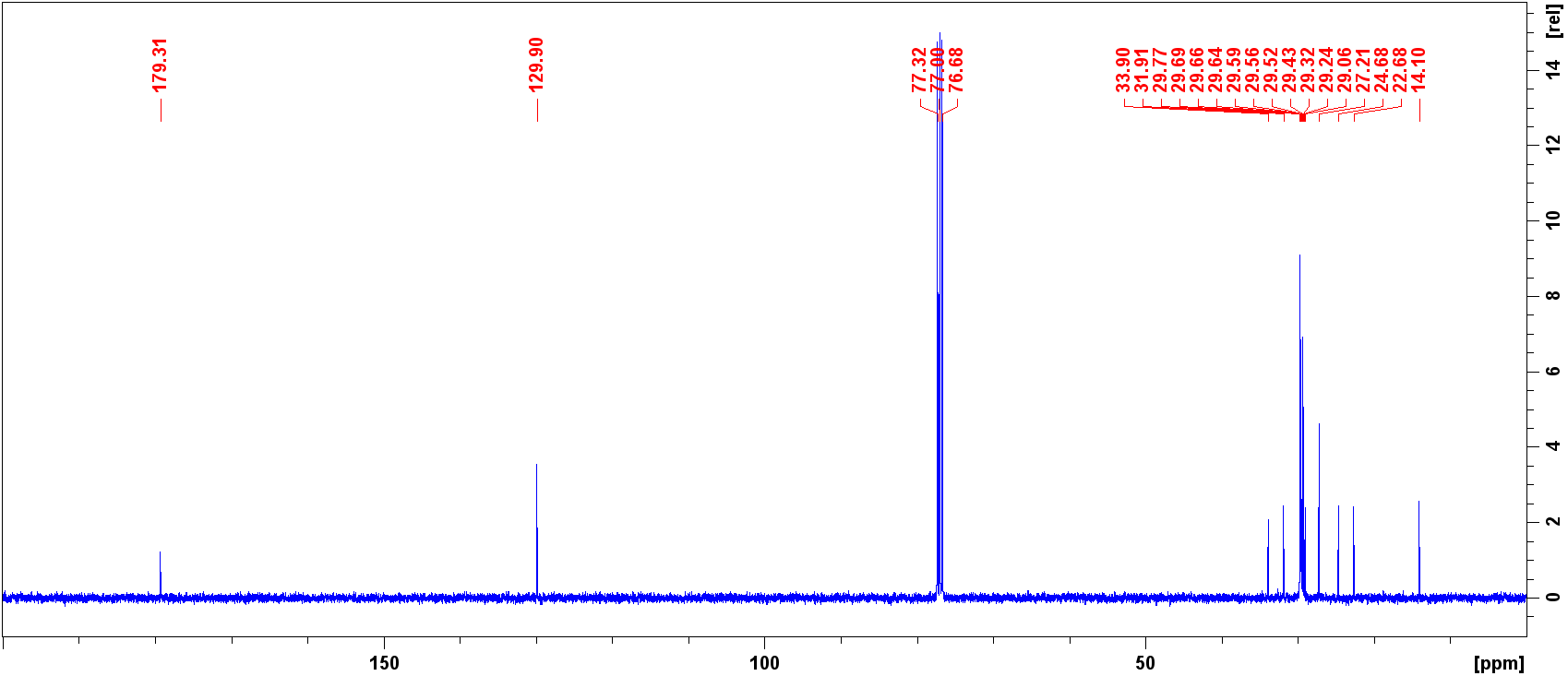
**

**Compound 8**

**
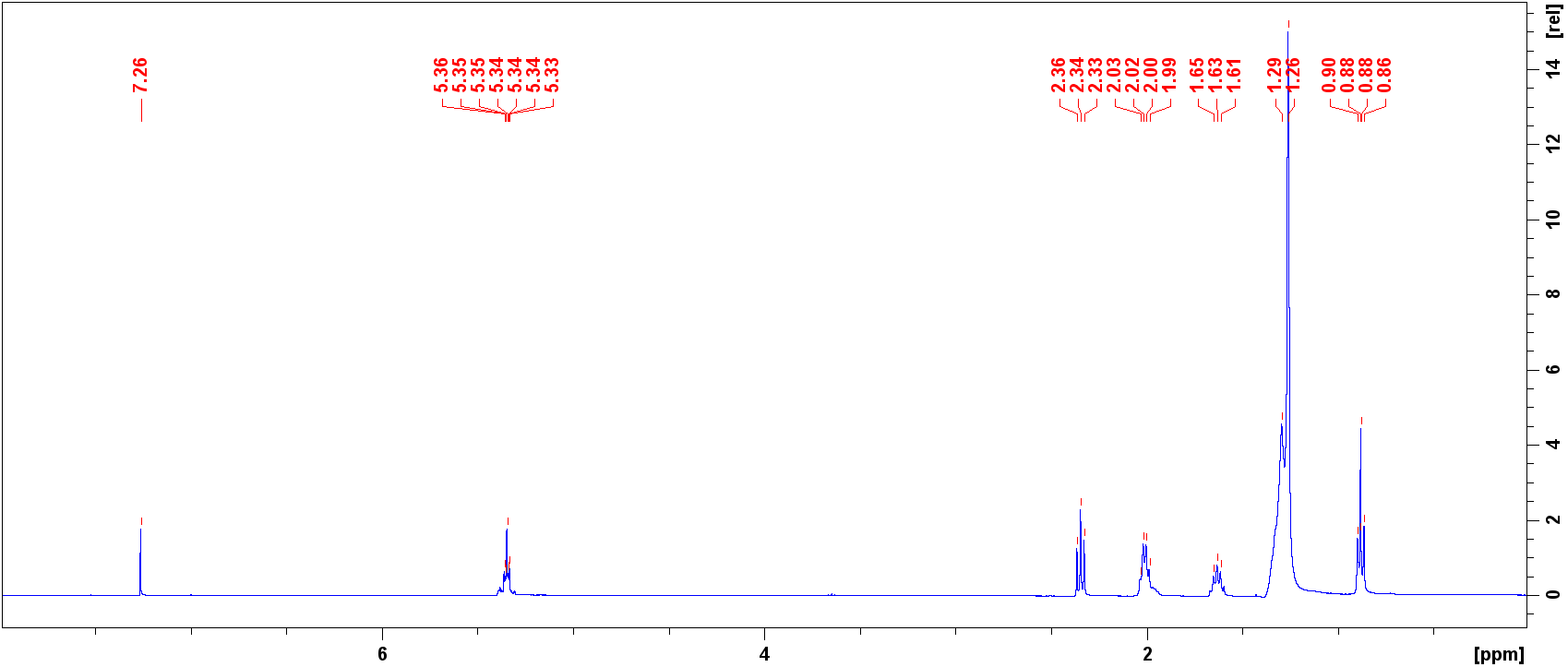
**

**
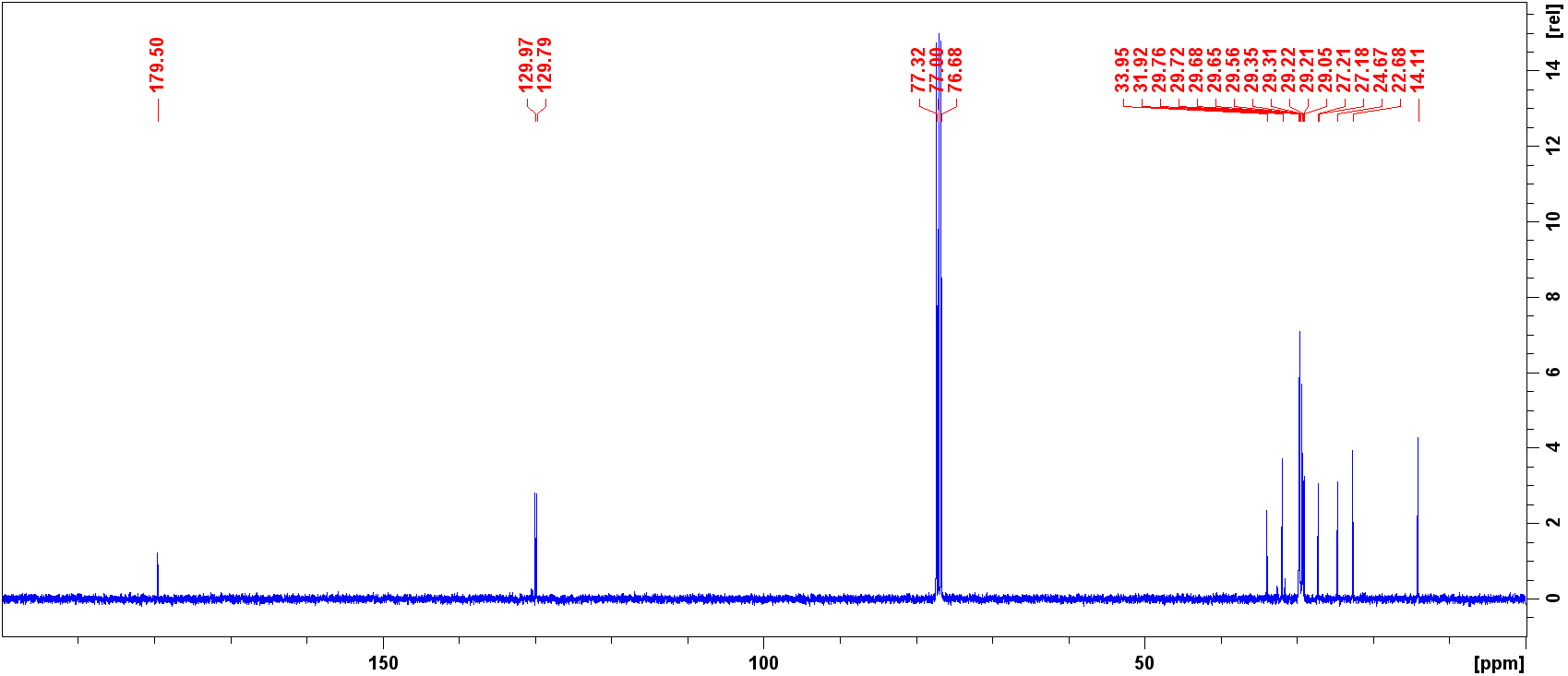
**

**Compound 9**

**
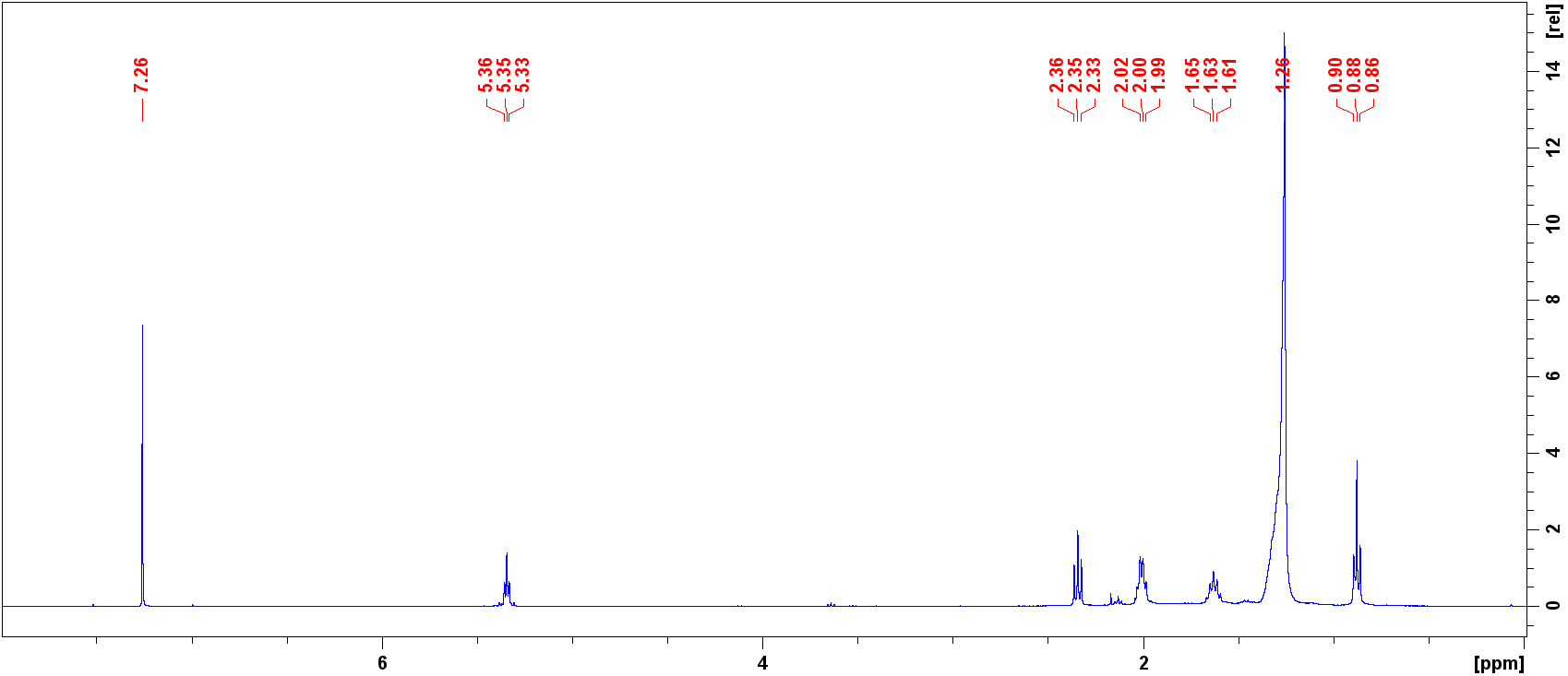
**

**
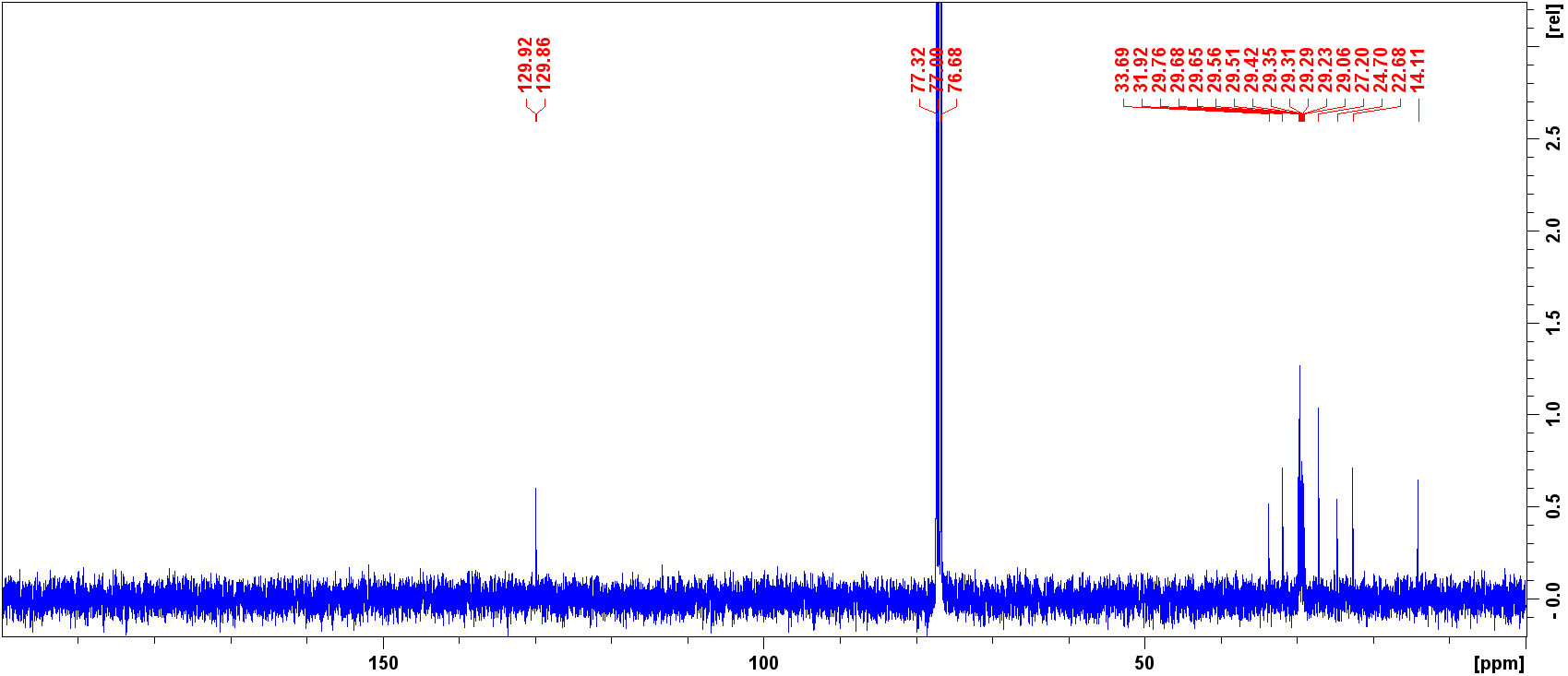
**

**Compound 10**

**
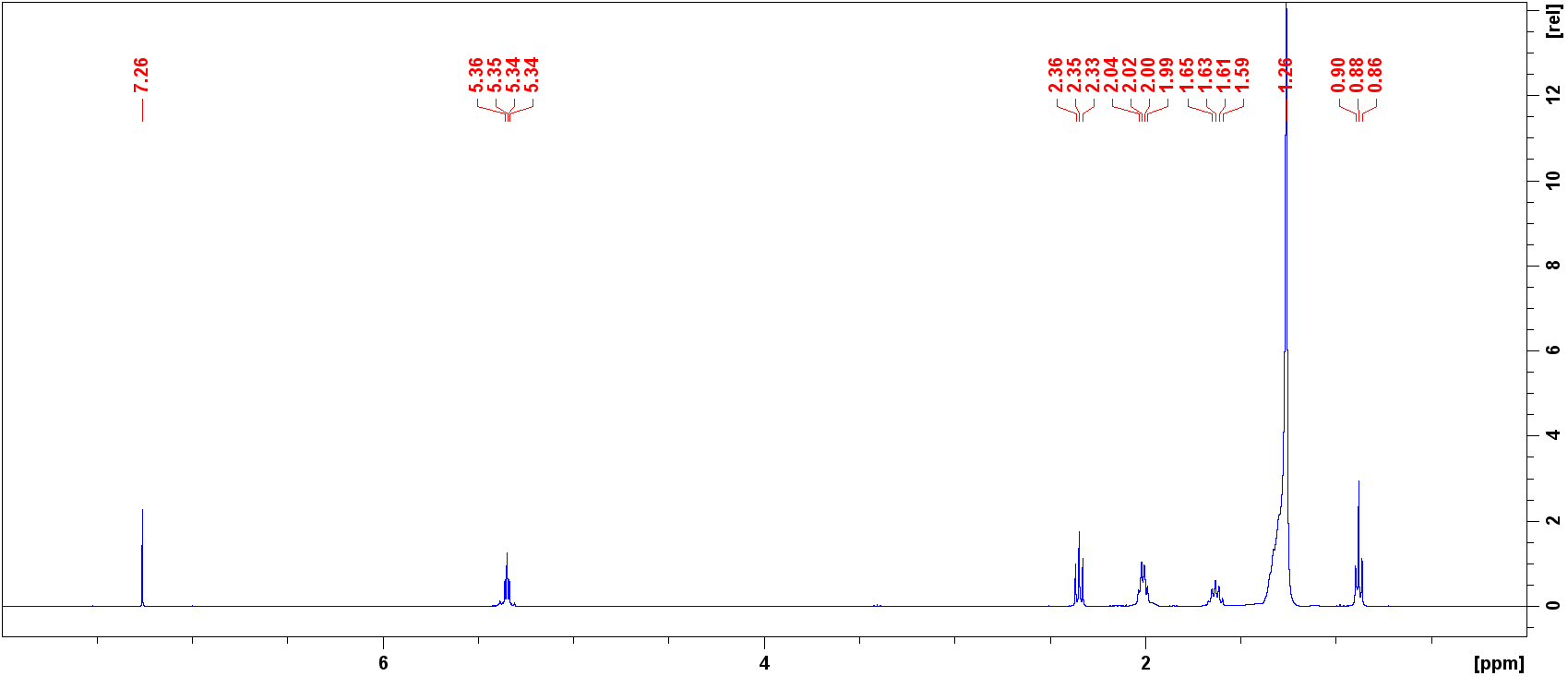
**

**
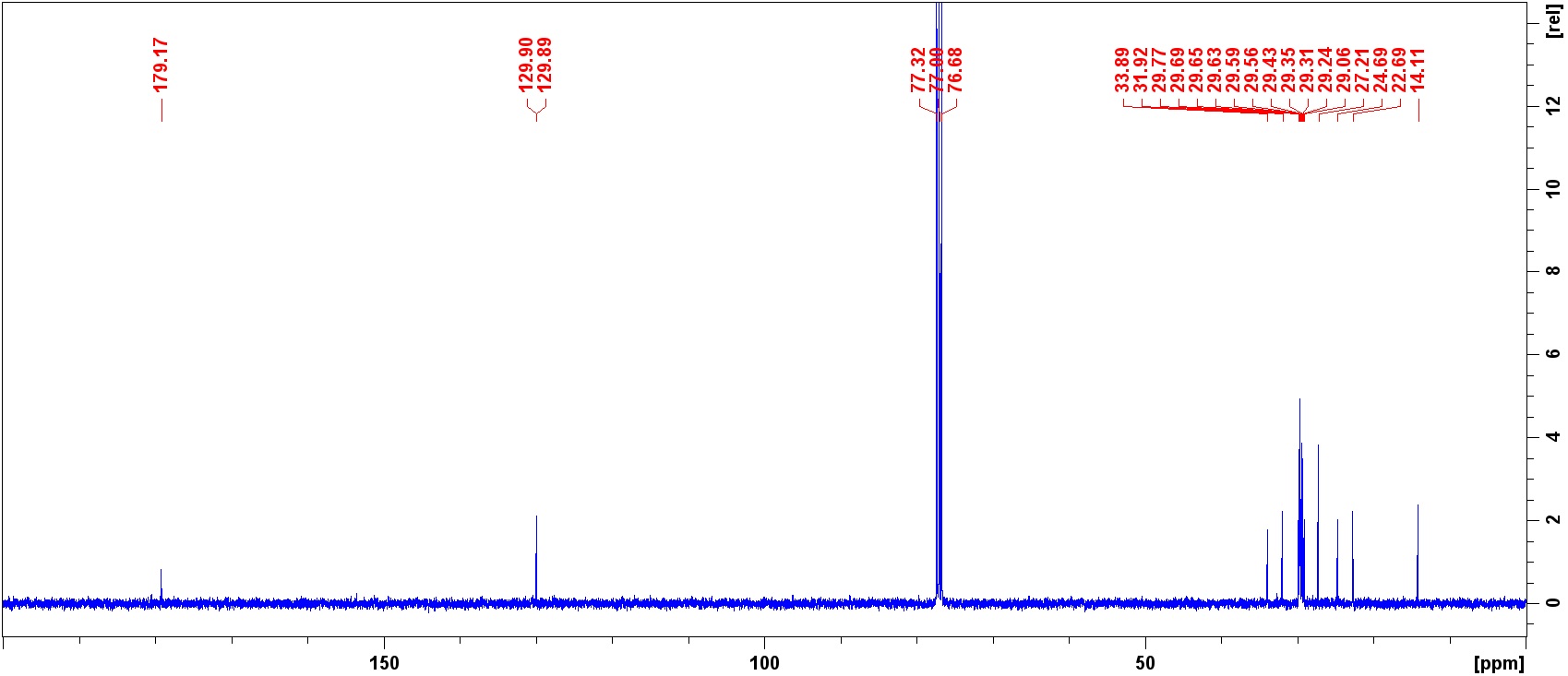
**

**Compound 11**

**
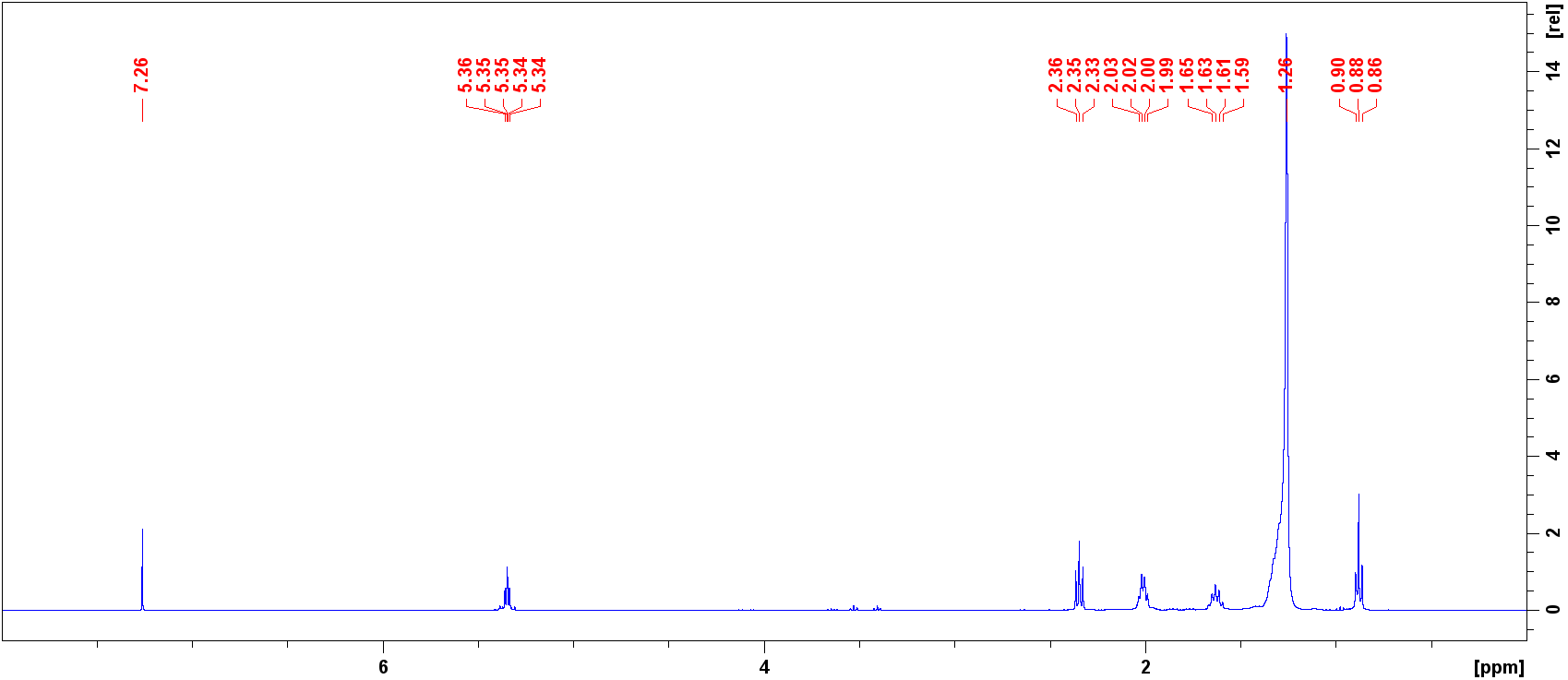
**

**
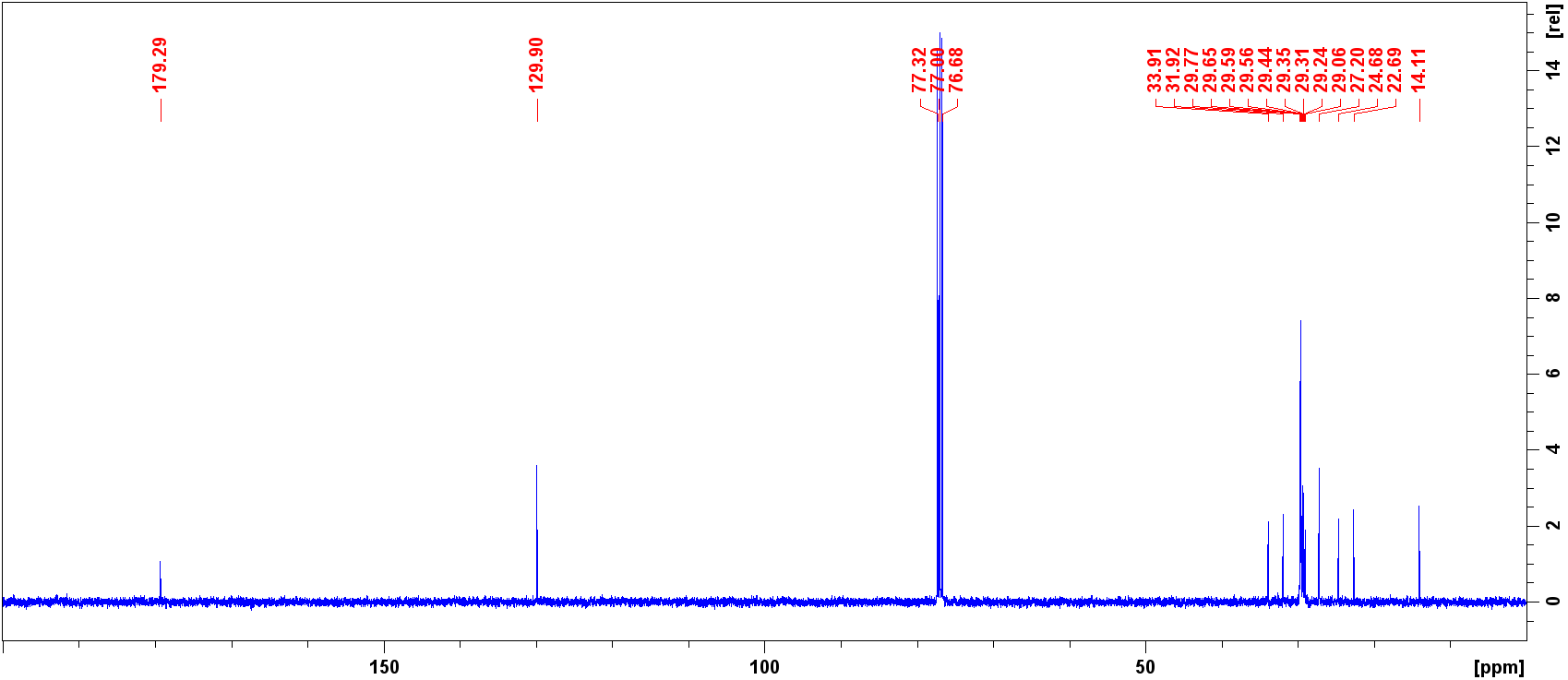
**

**Compound 12**

**
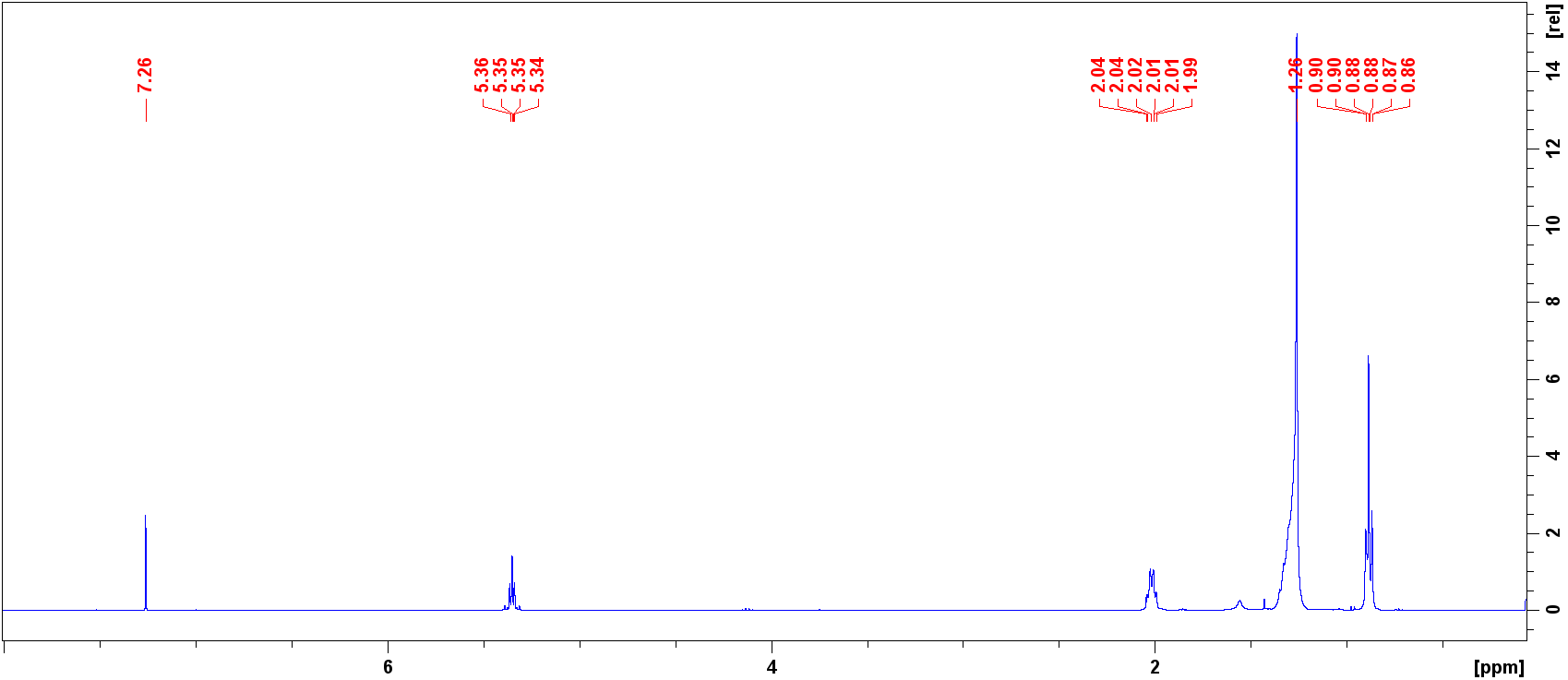
**

**
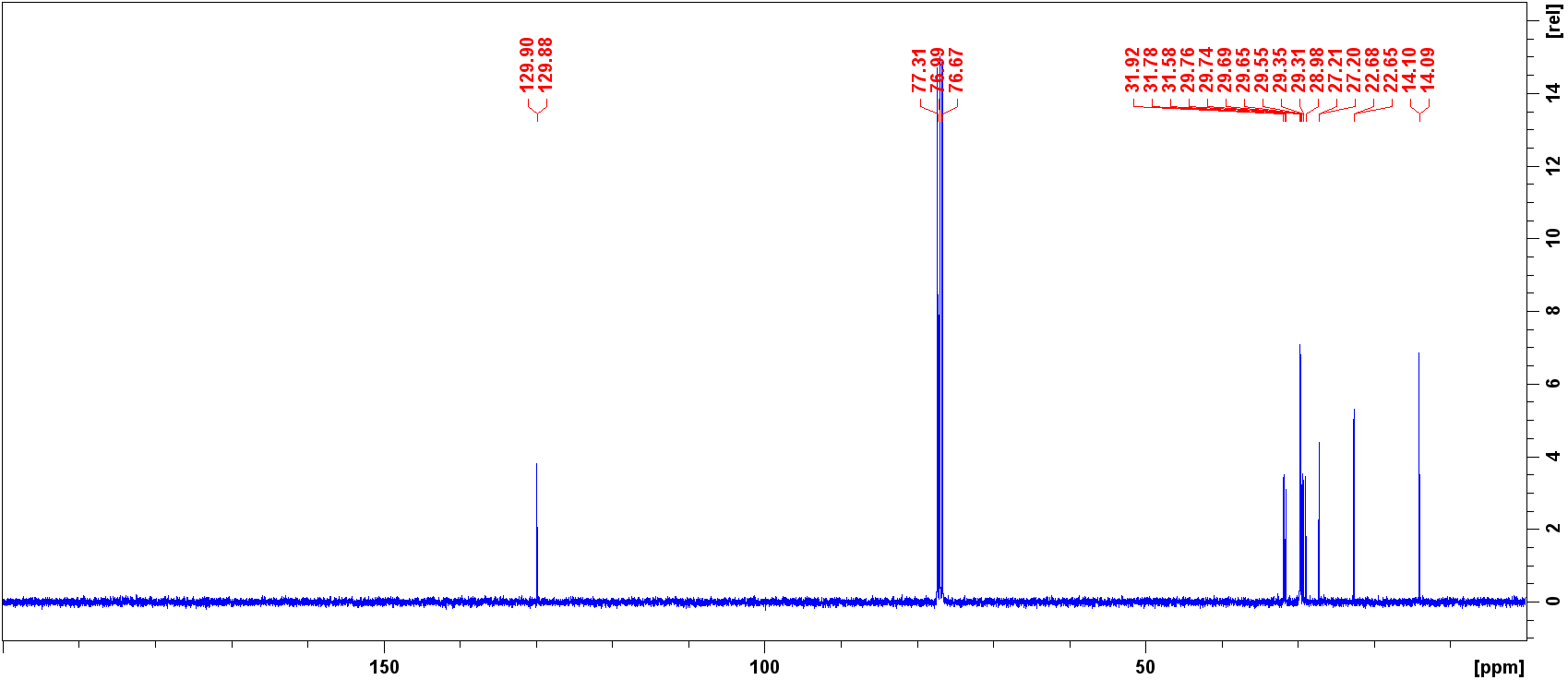
**

**Compound 13**

**
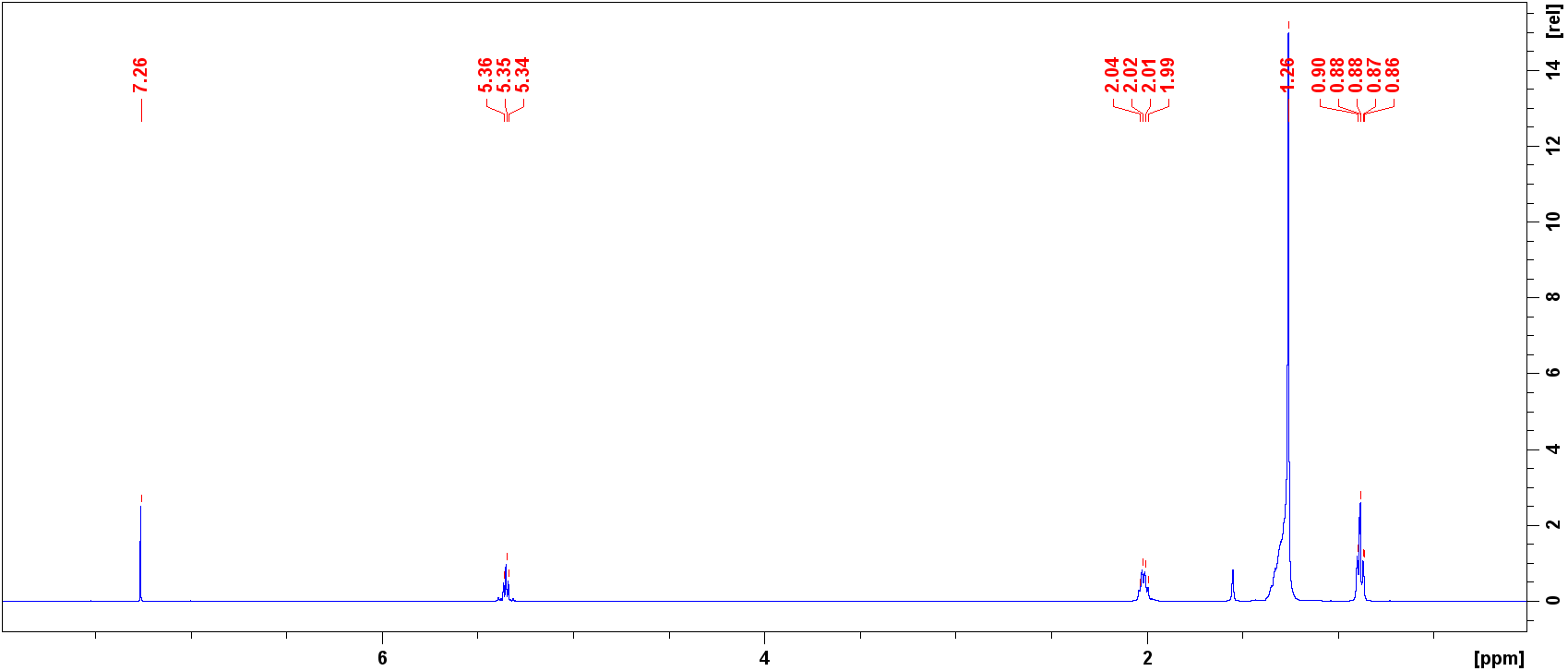
**

**
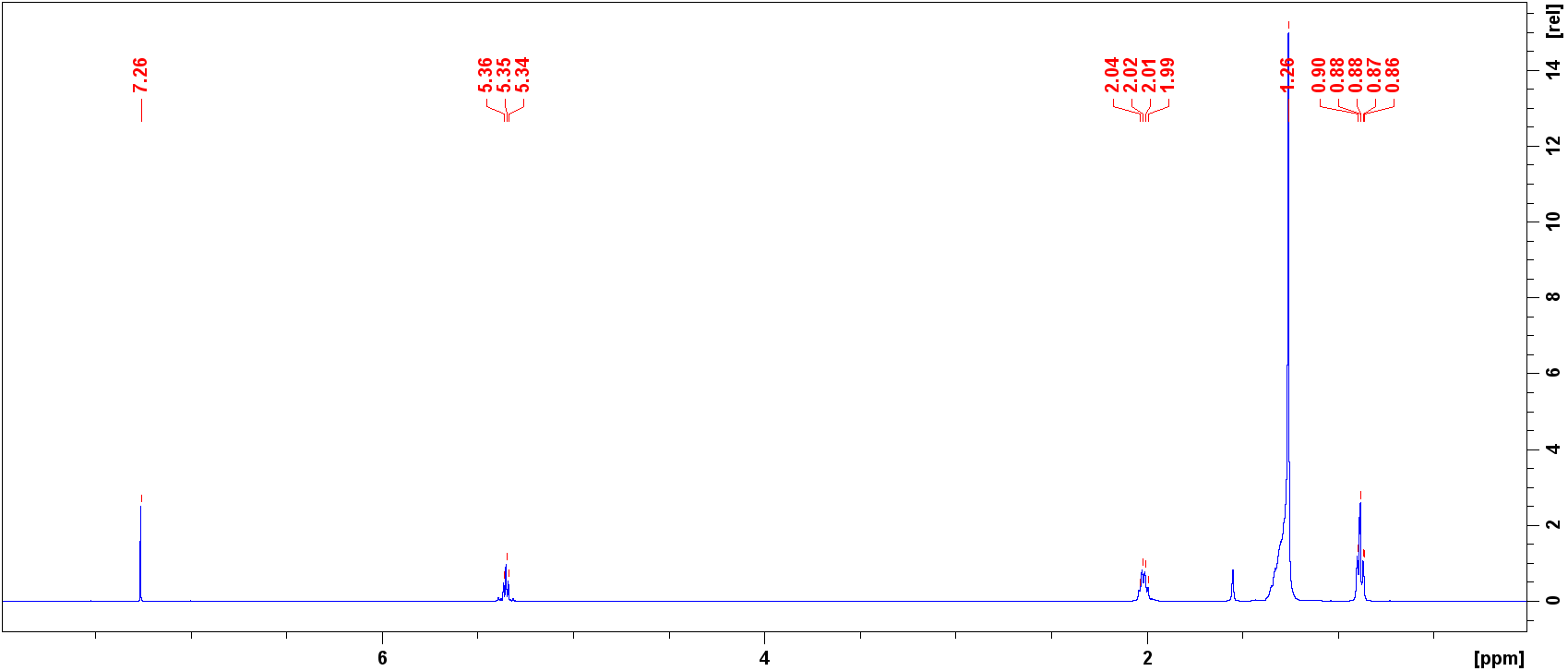
**

**Compound 14**

**
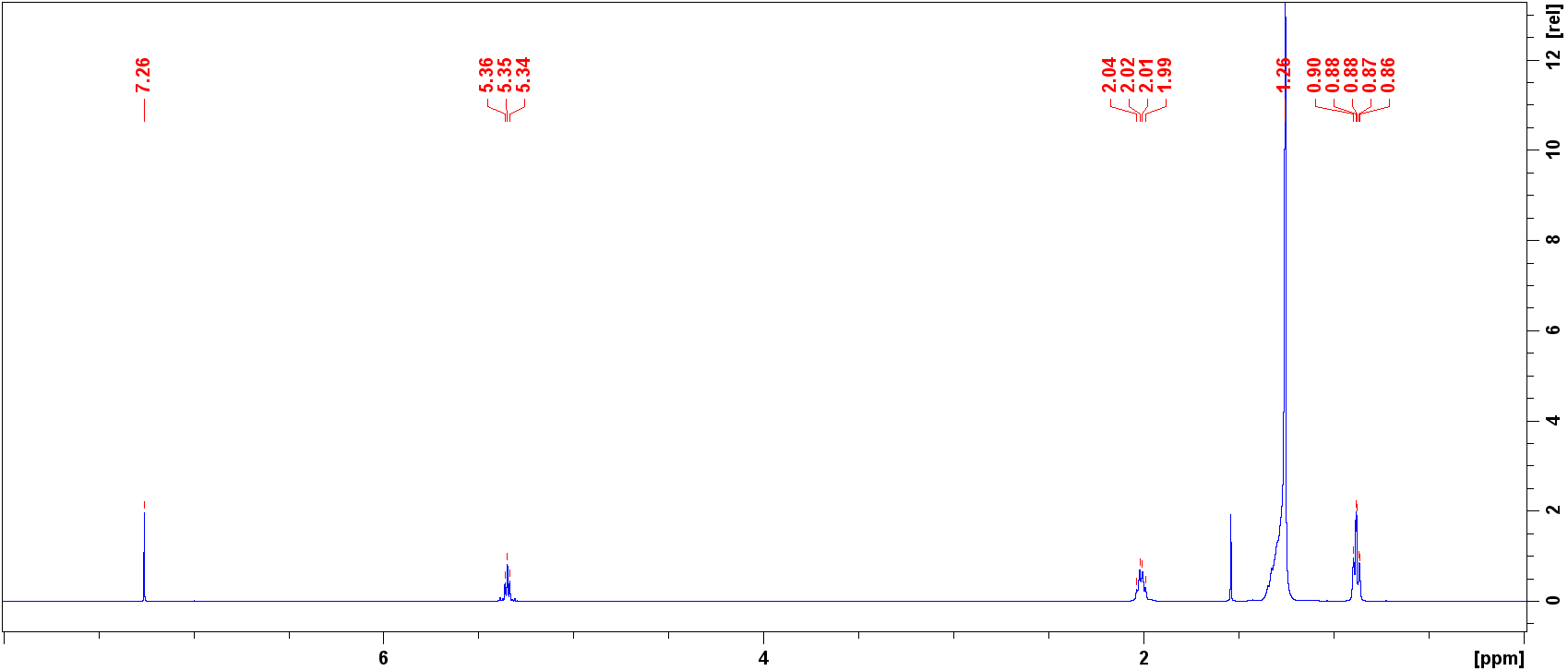
**

**
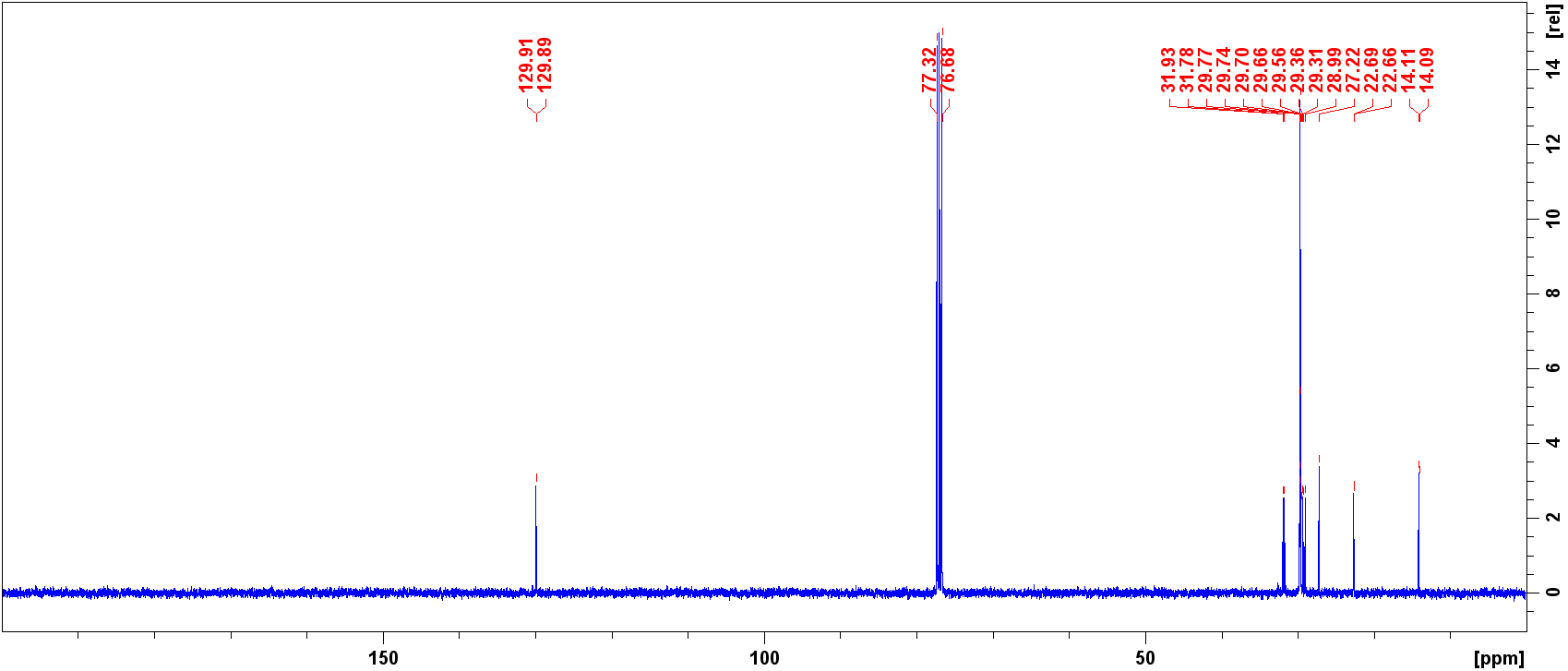
**

**Compound 15**

**
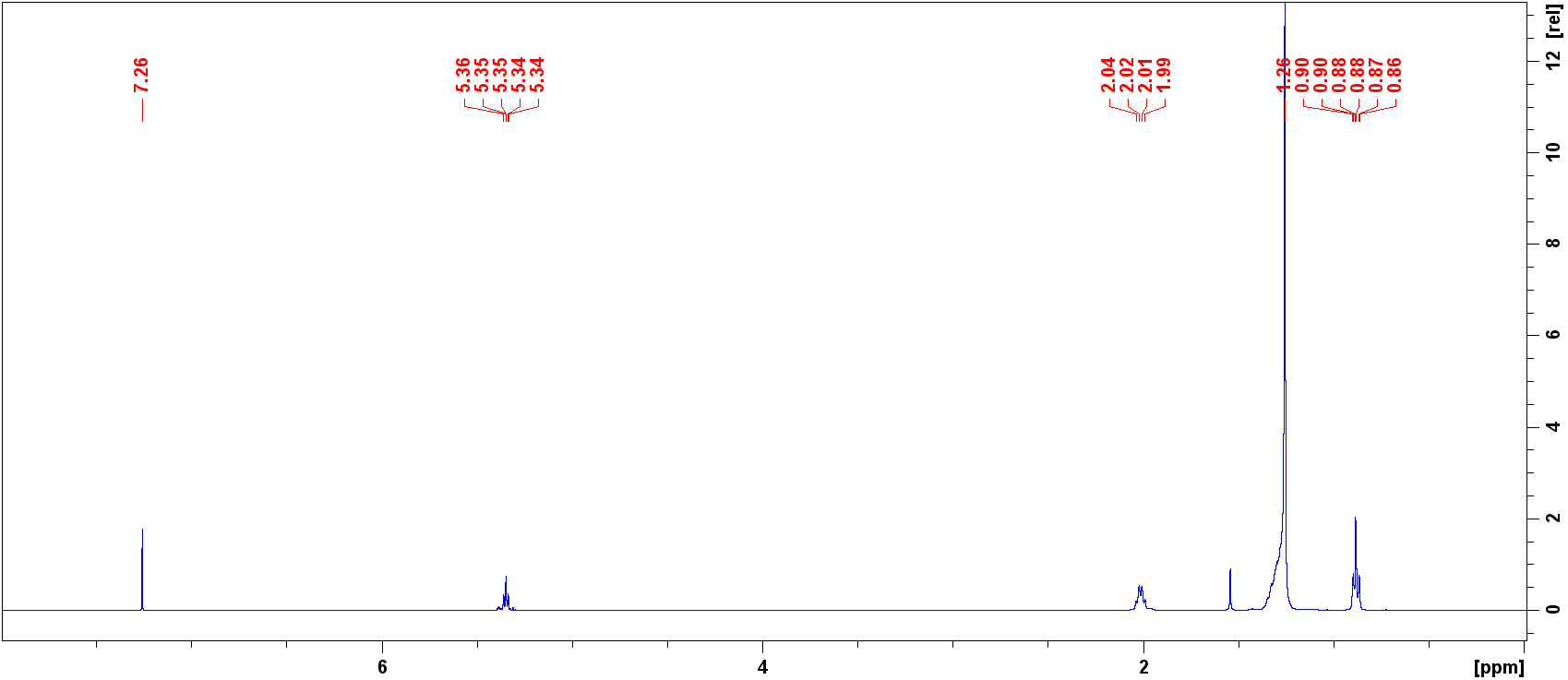
**

**
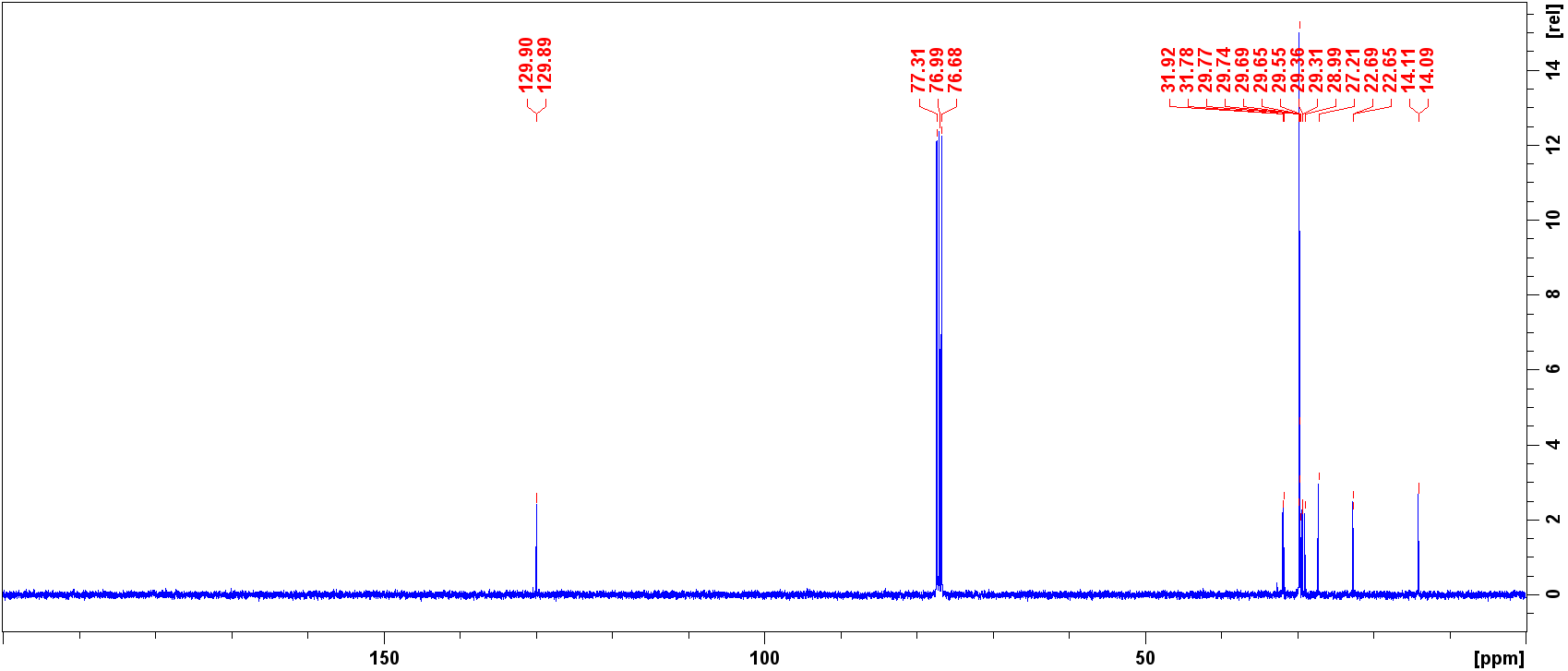
**

**Compound 16**

**
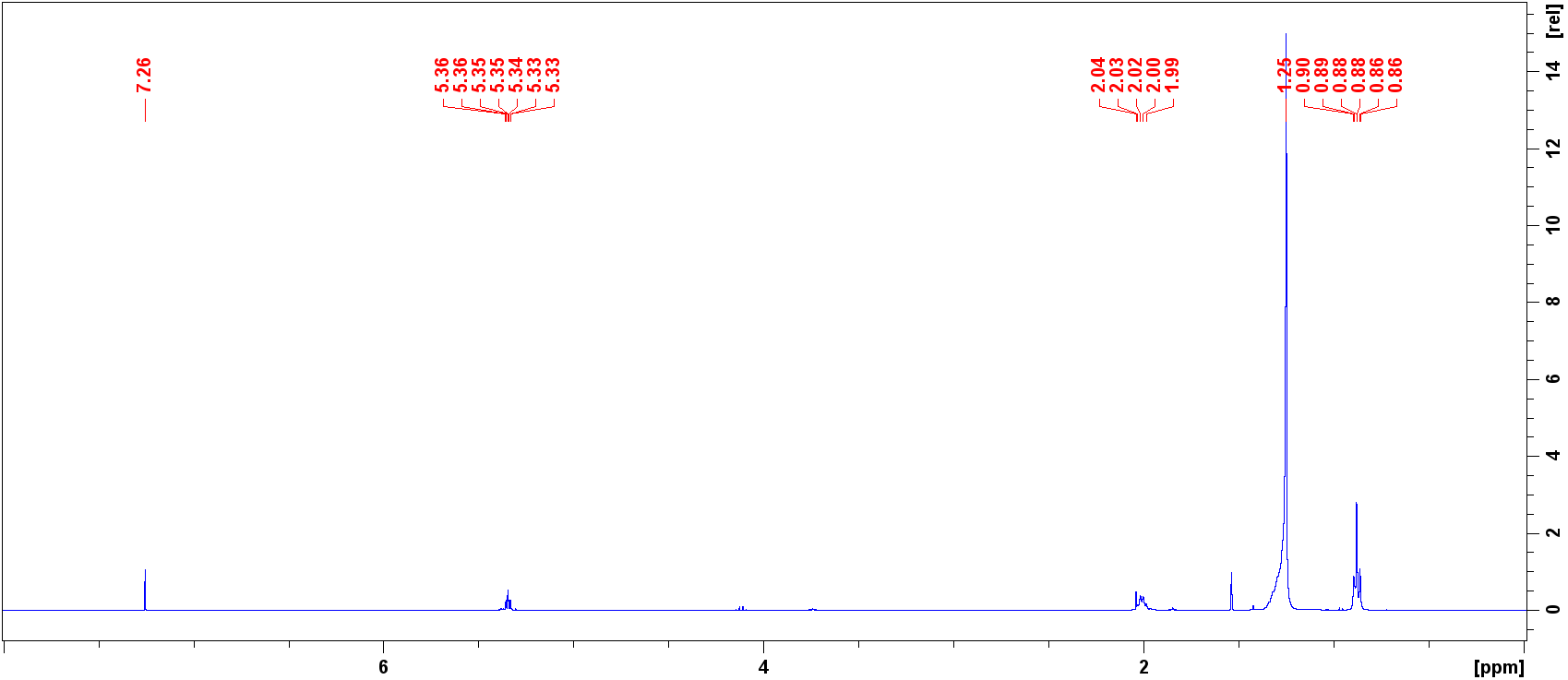
**

**
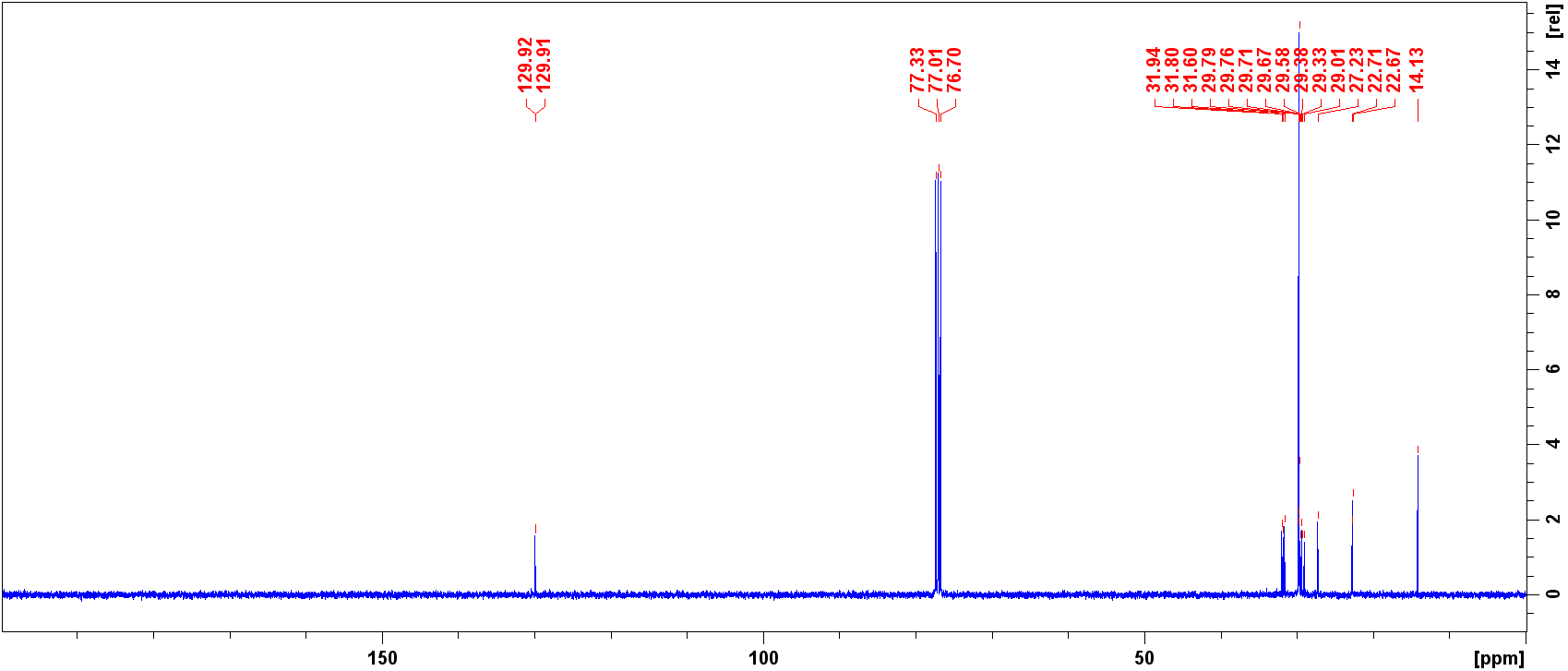
**

**Compound 17**

**
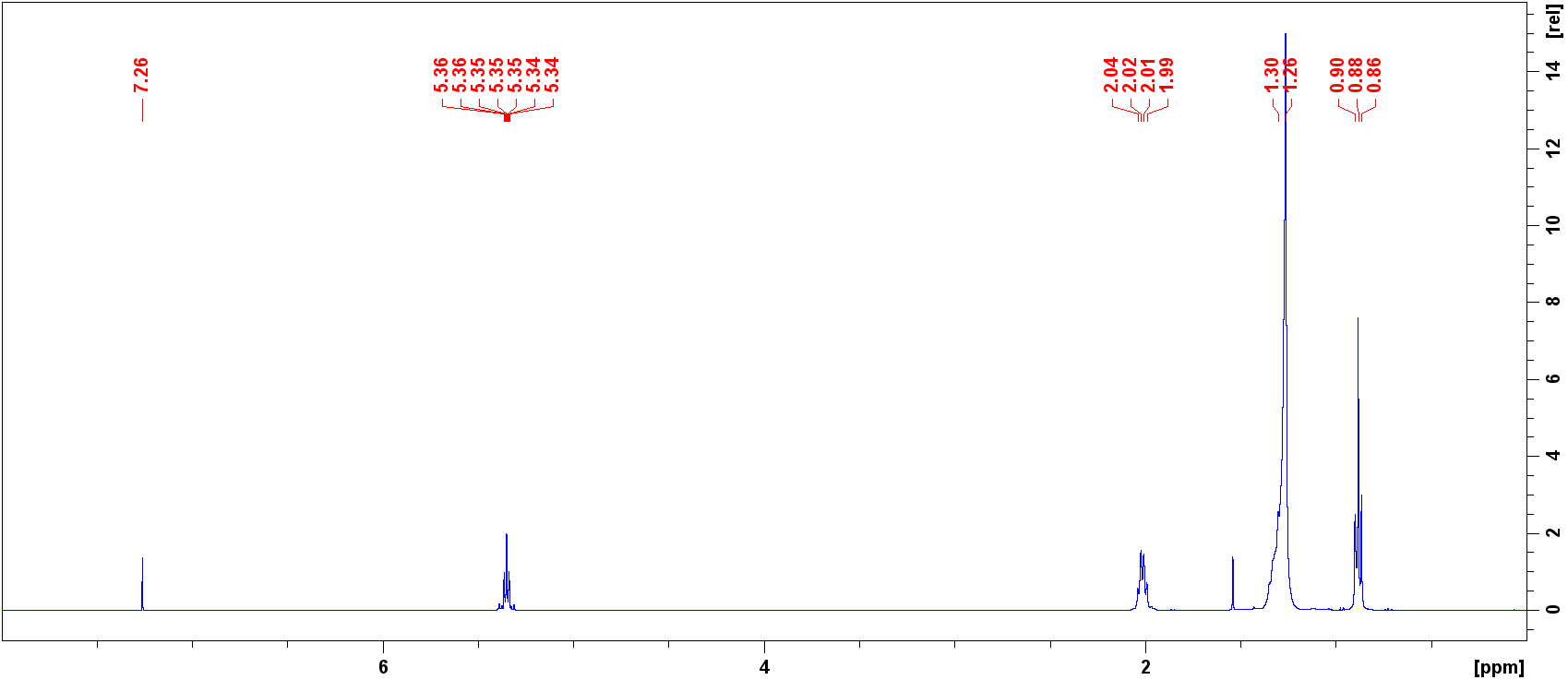
**

**
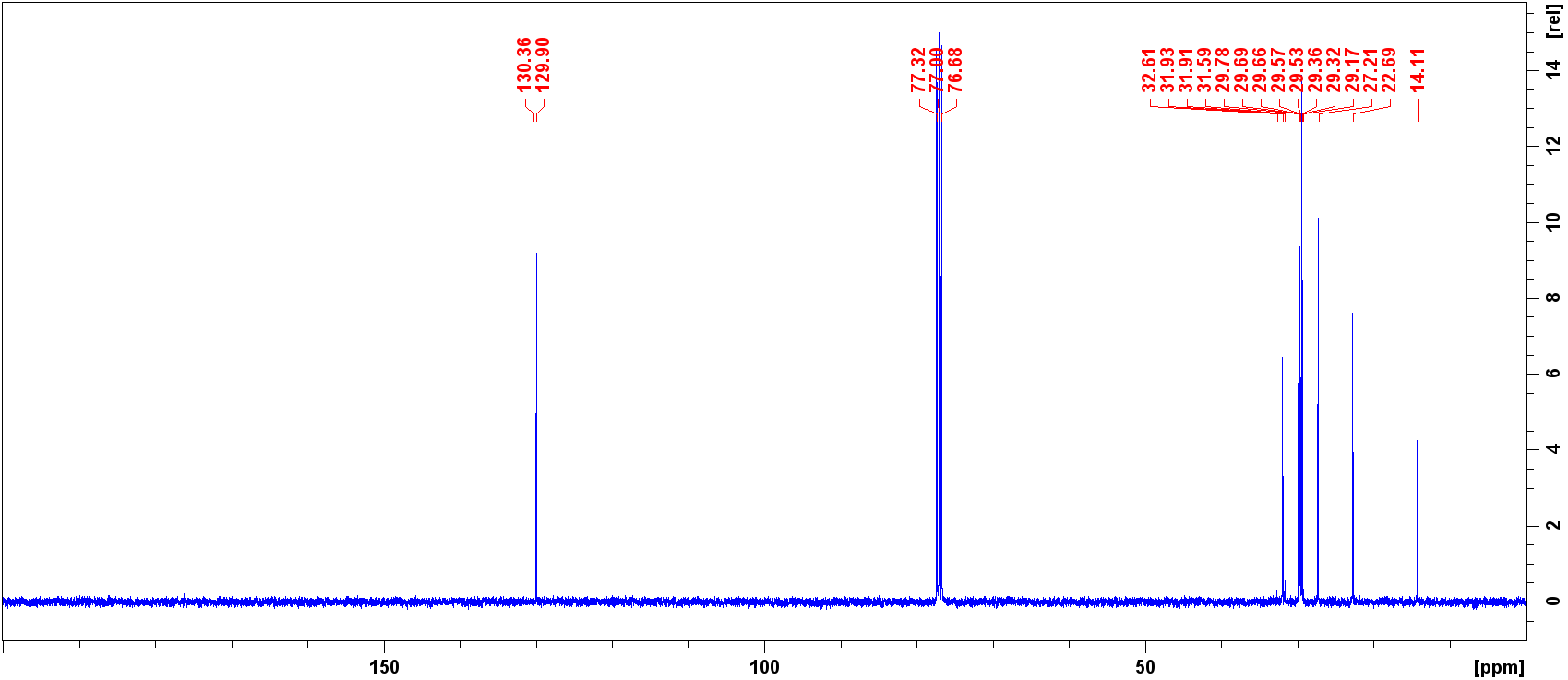
**

**Compound 18**

**
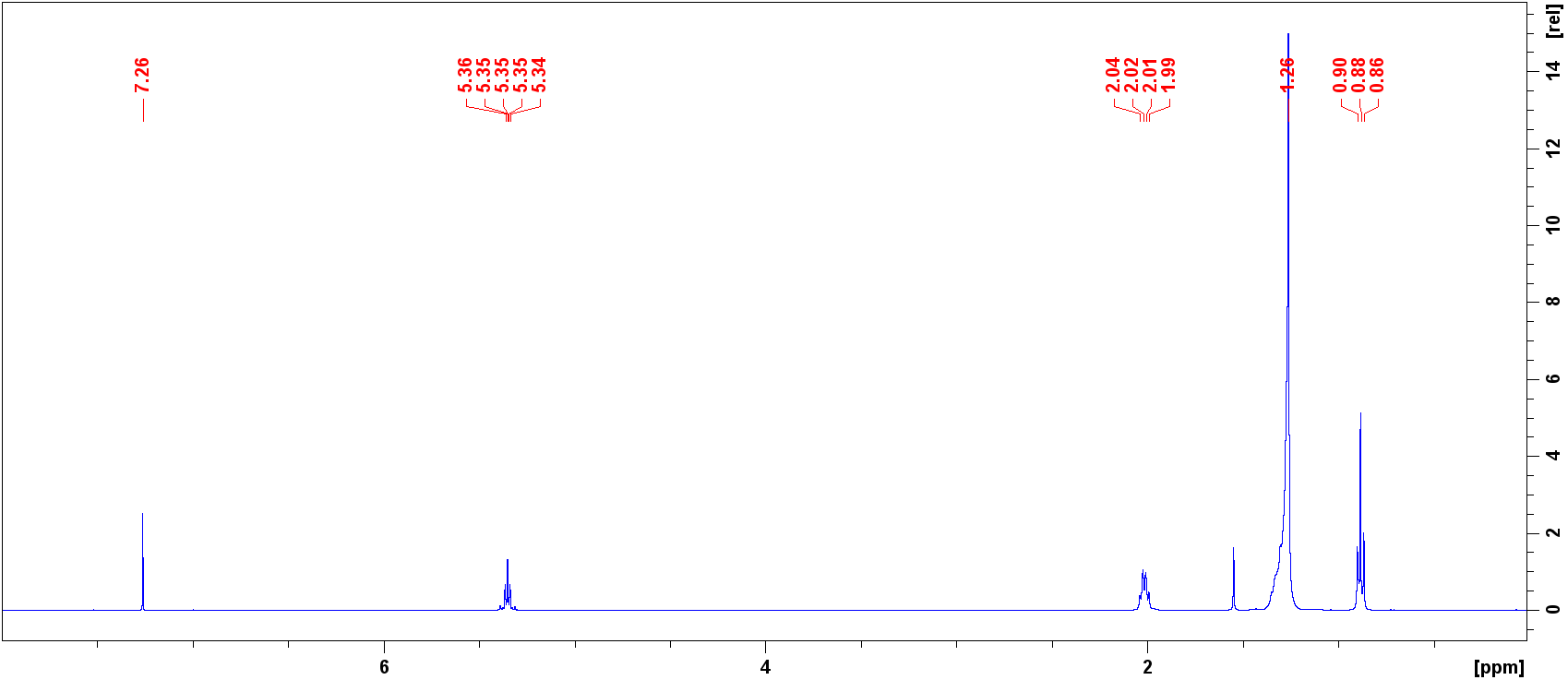
**

**
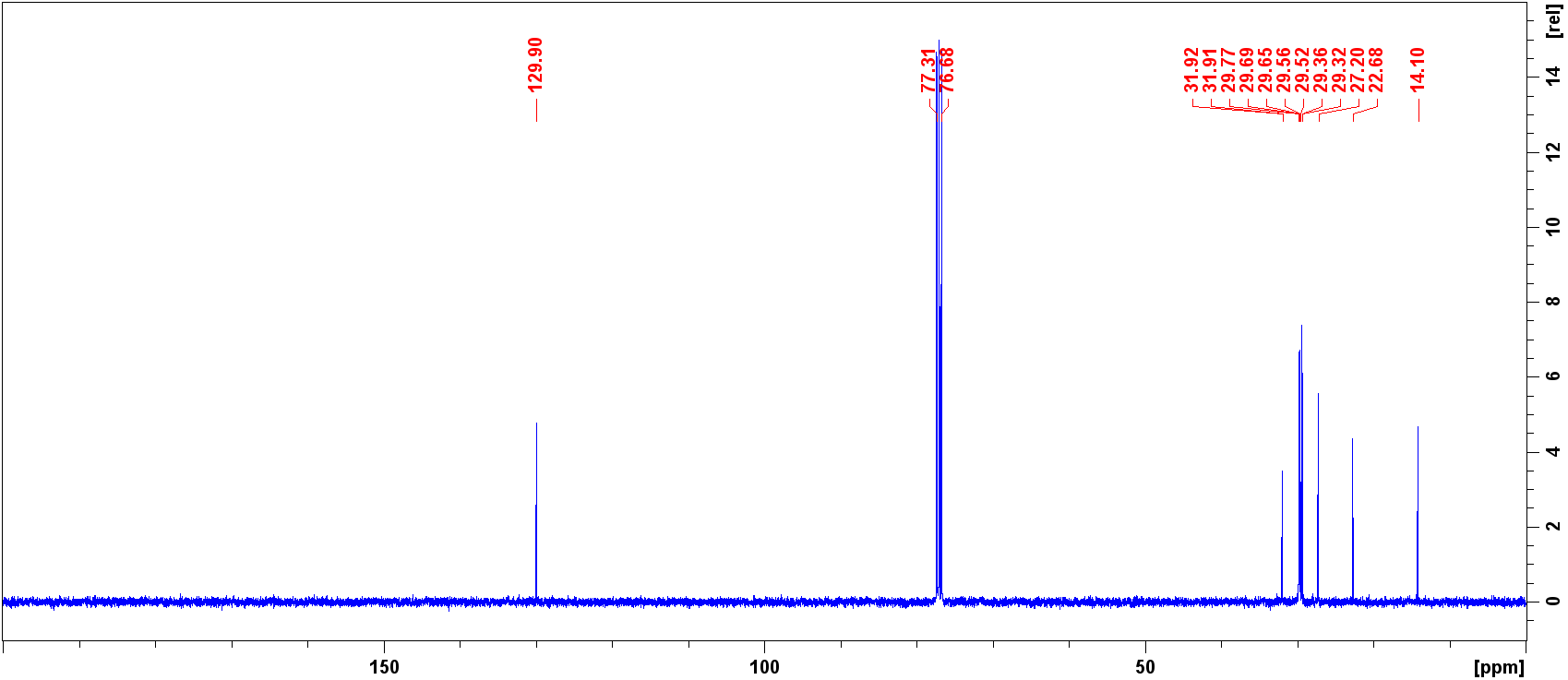
**

**Compound 19**

**
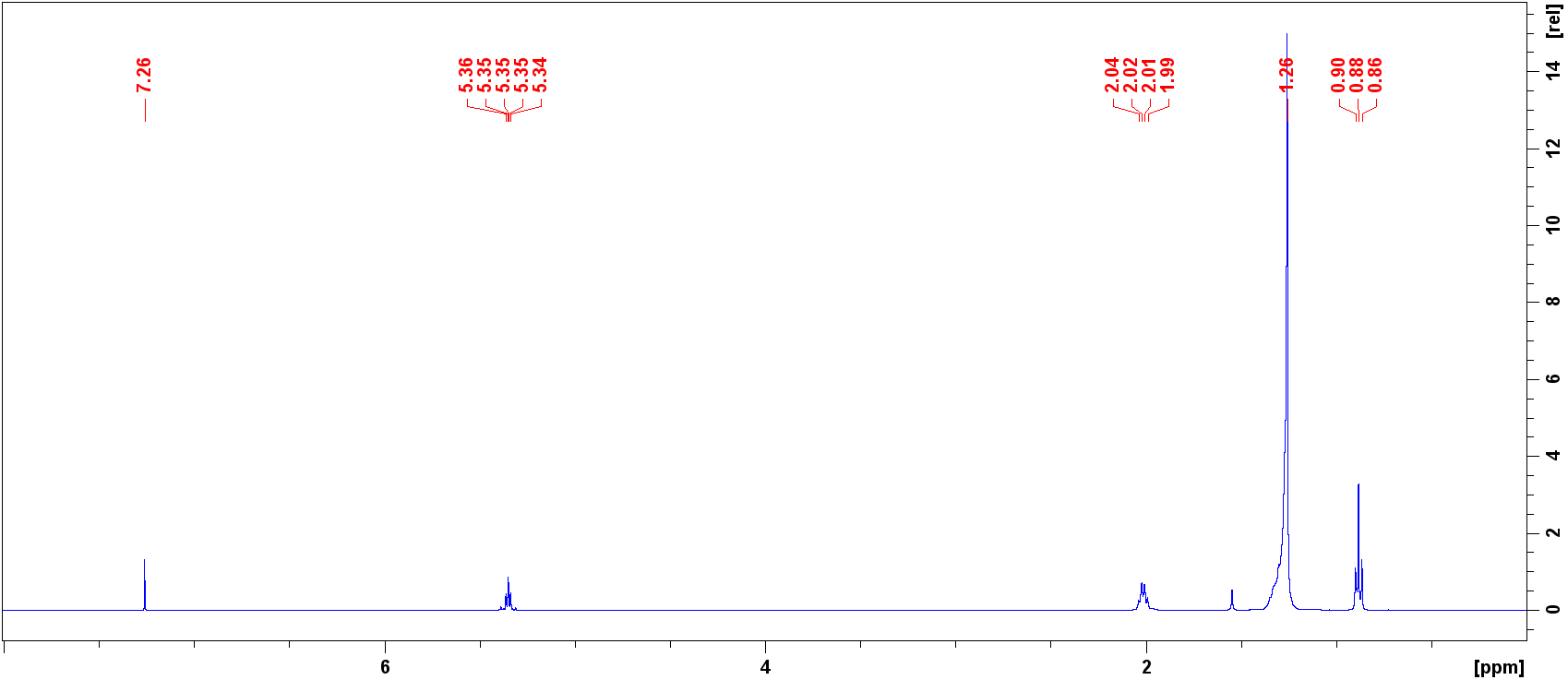
**

**
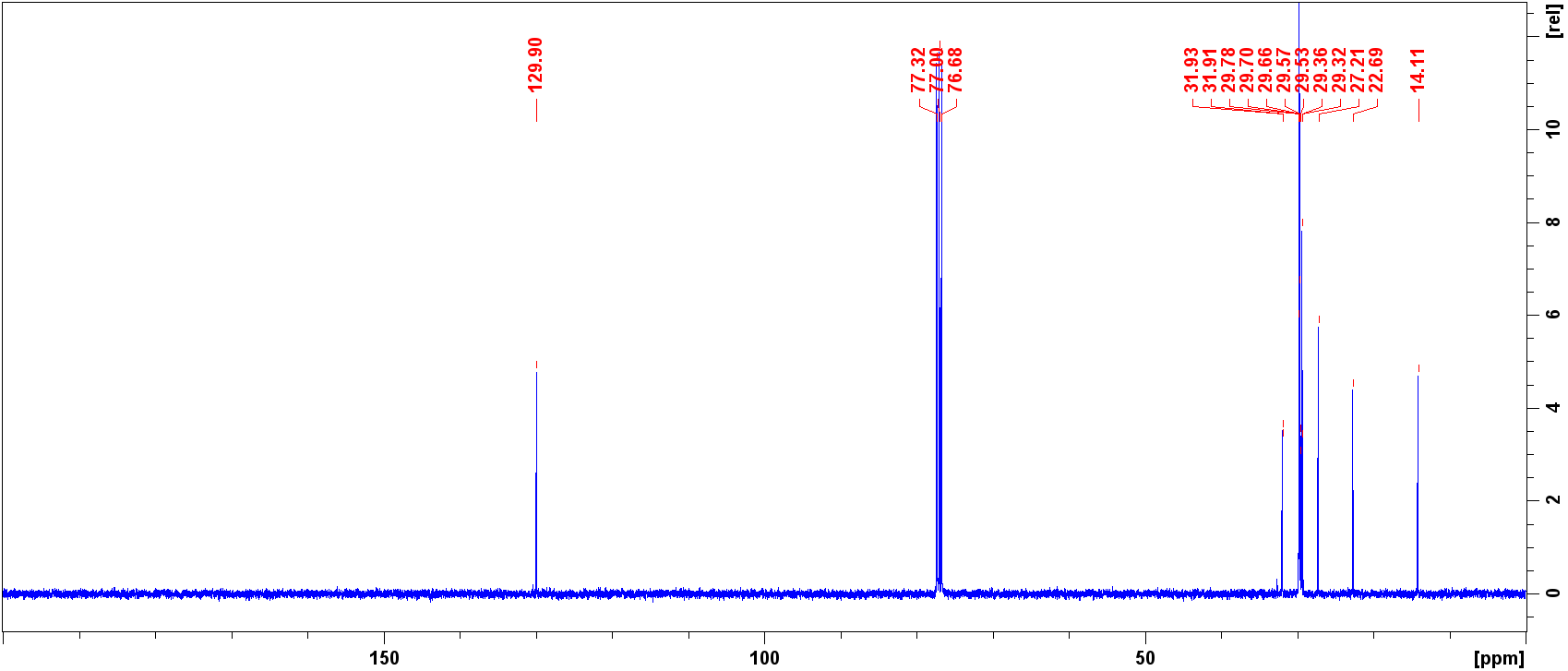
**

**Compound 20**

**
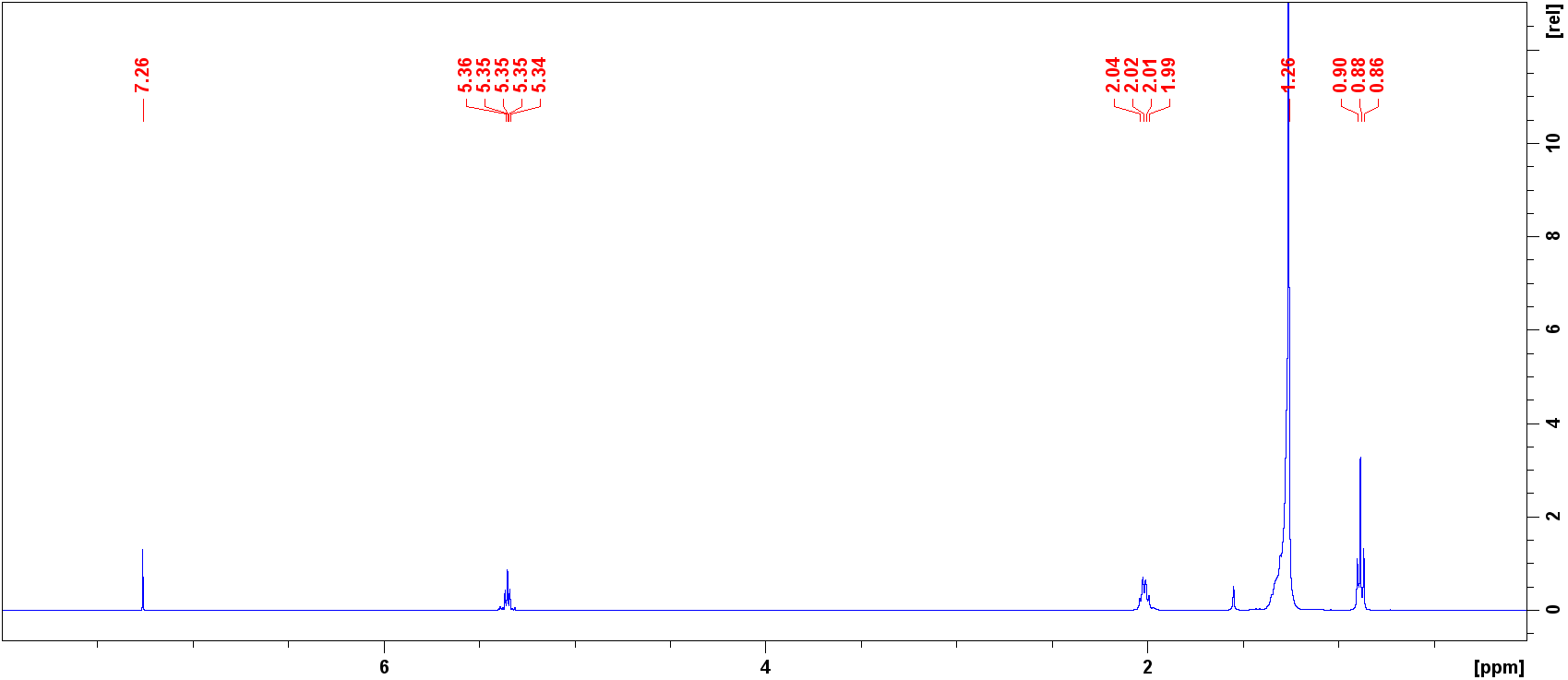
**

**
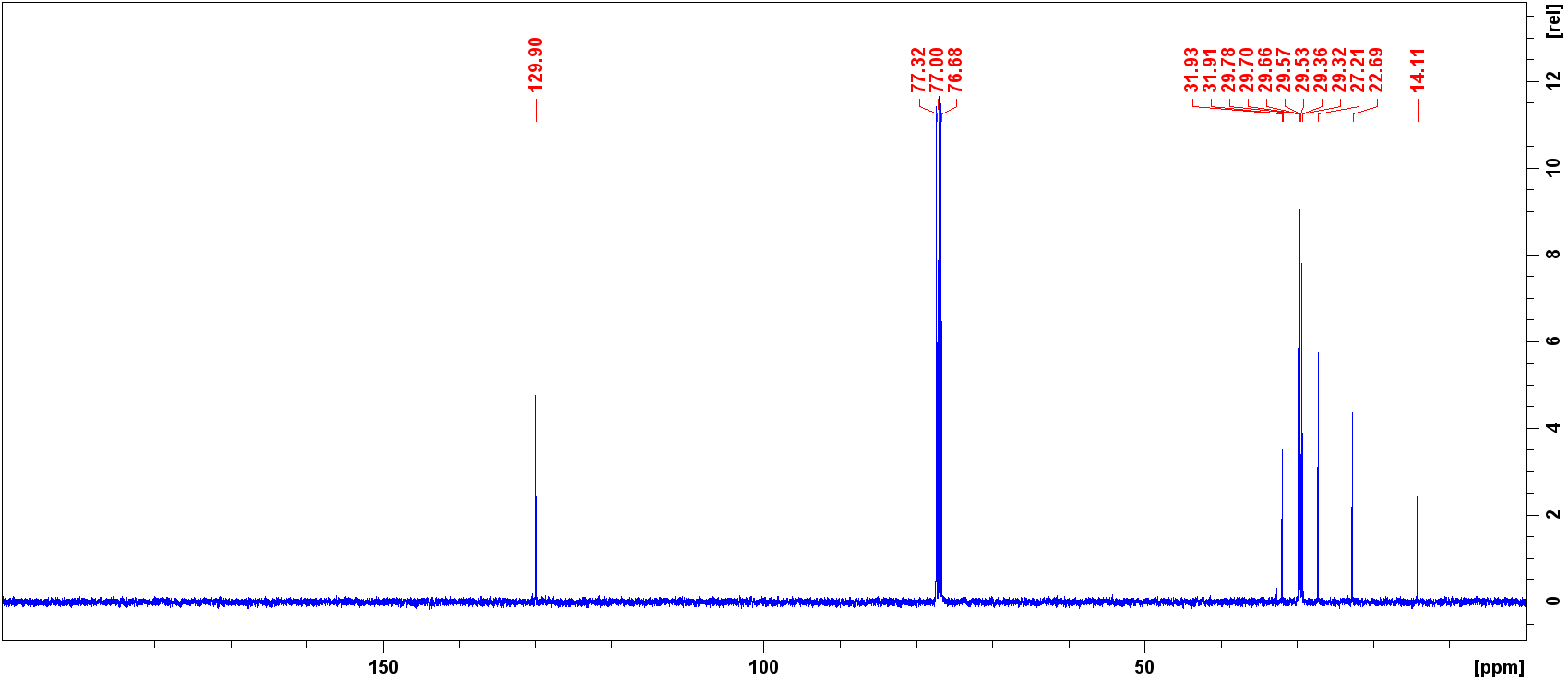
**

**Compound 21**


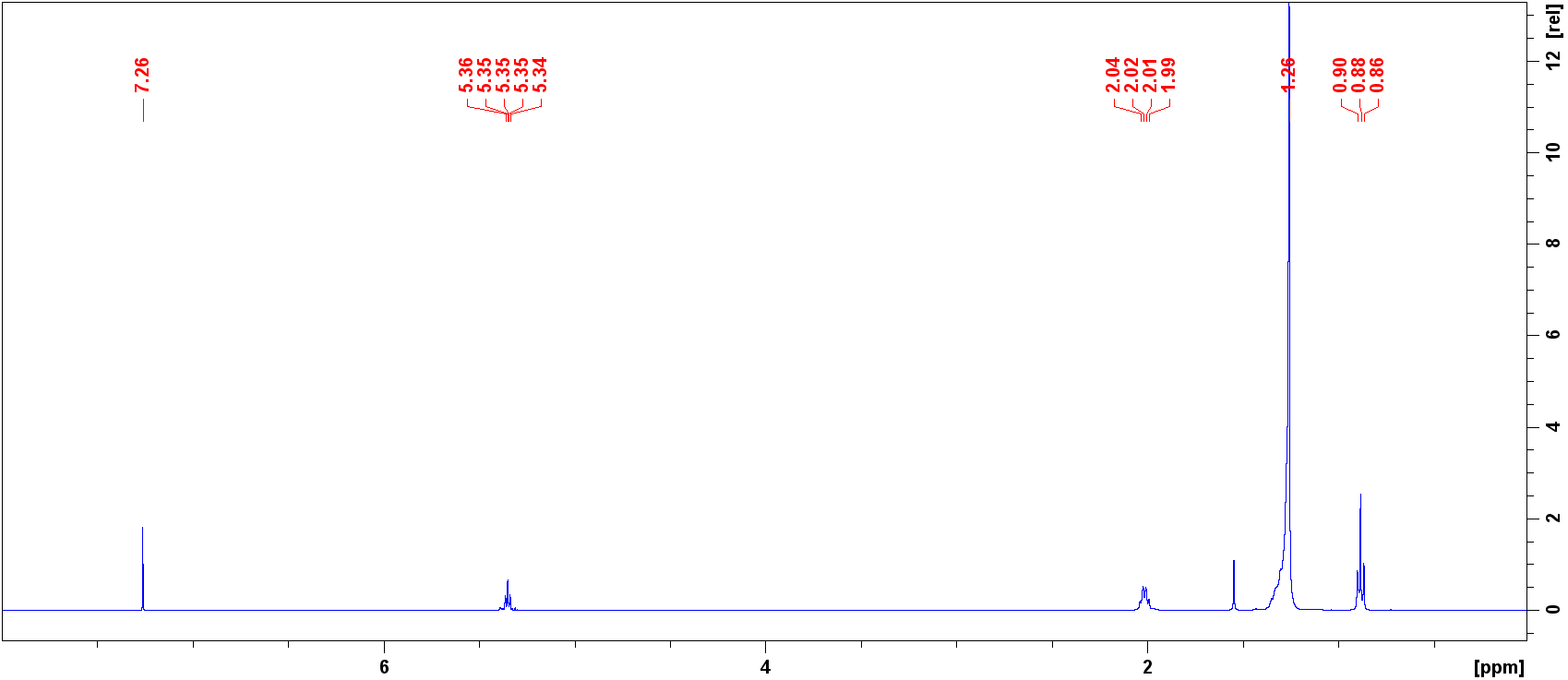


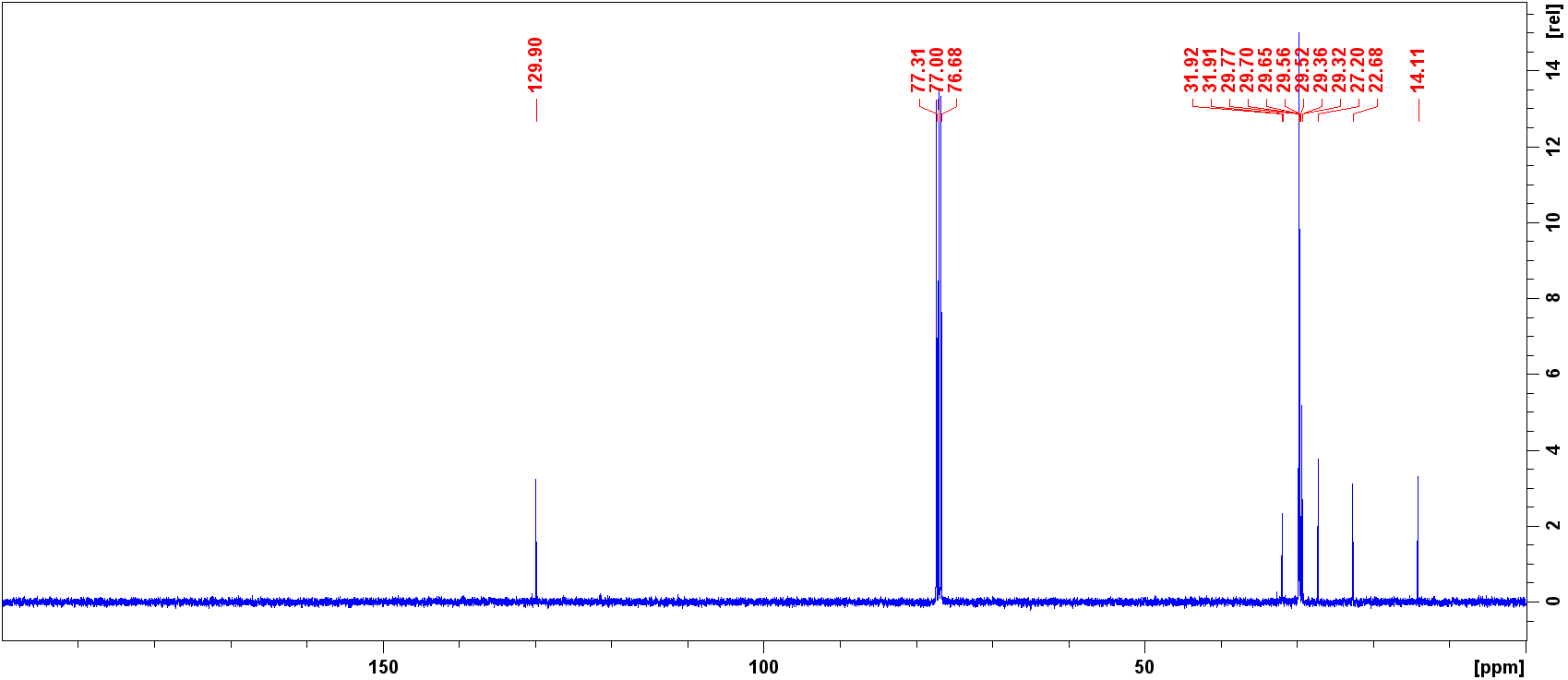


**Compound 22**


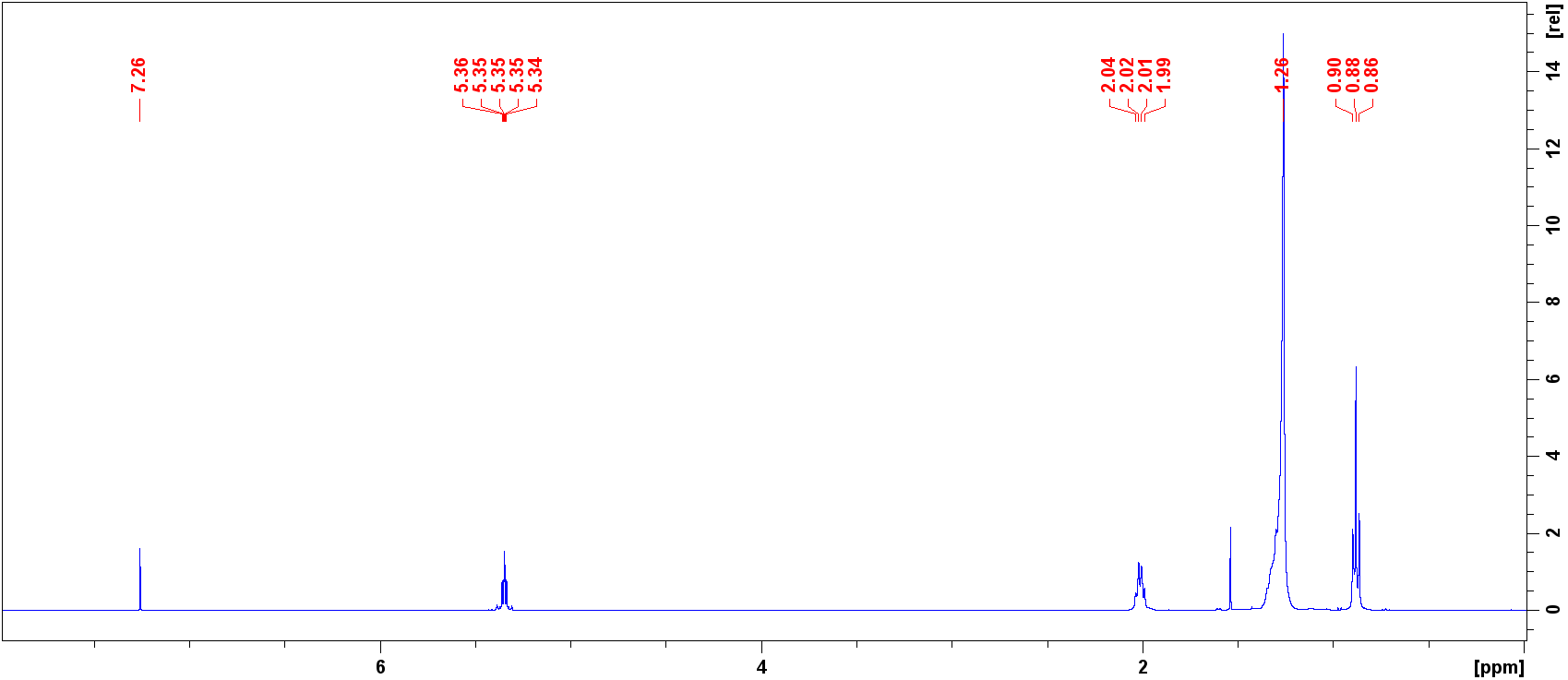


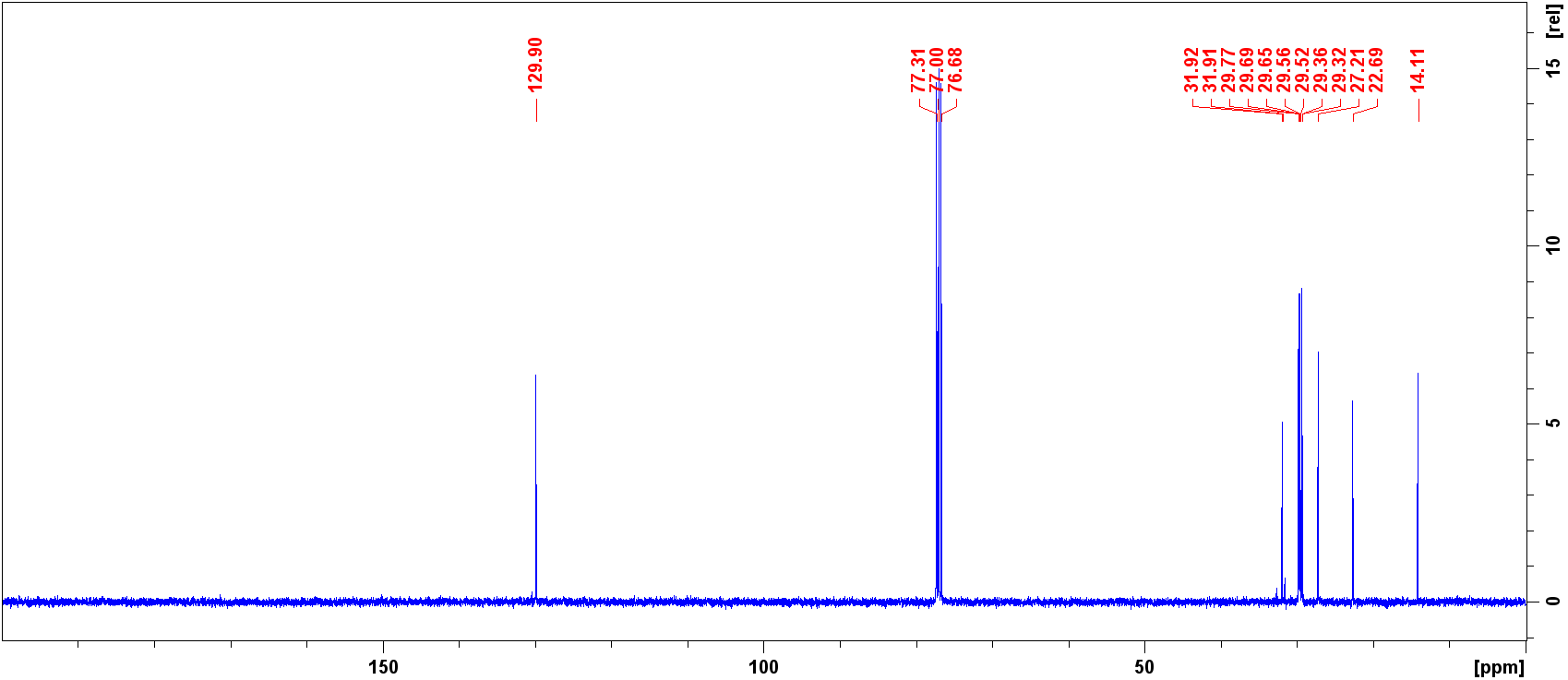


**Compound 23**

**
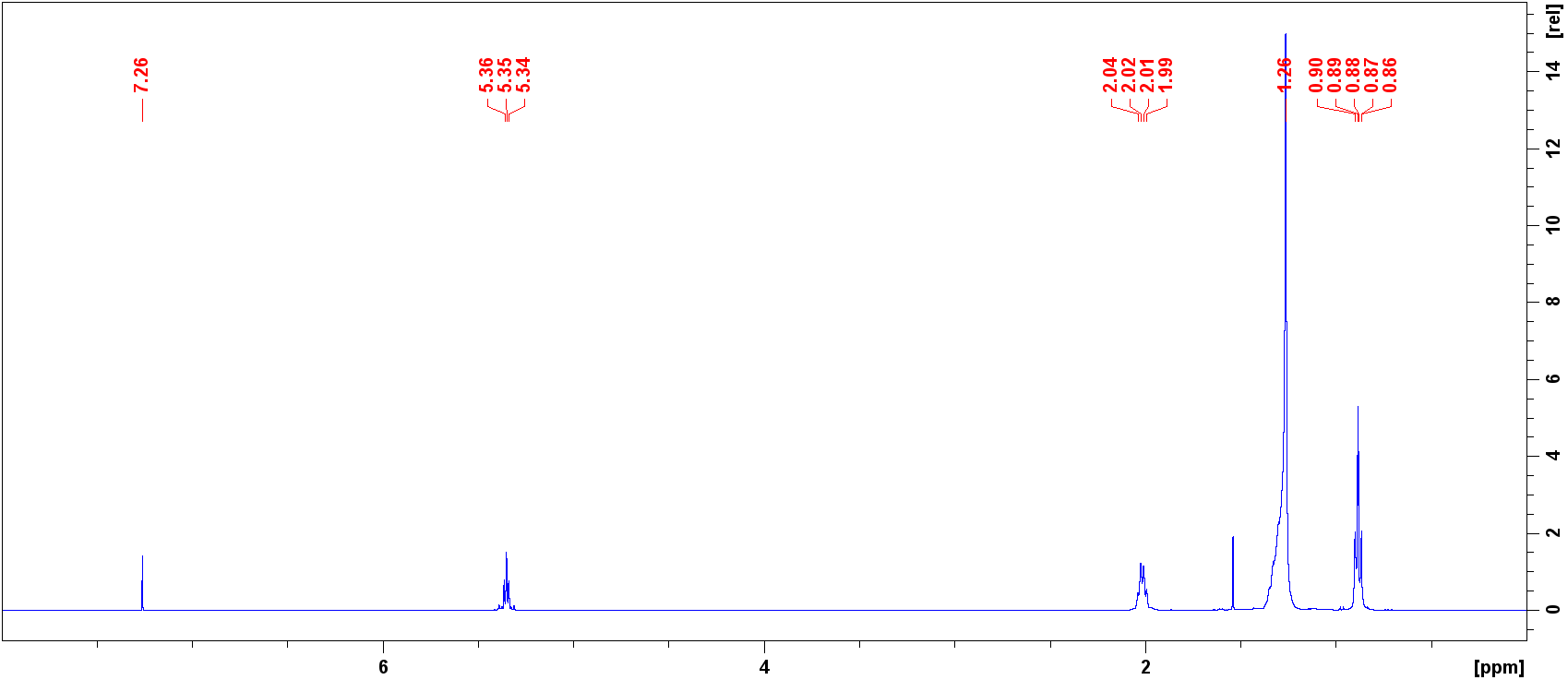
**

**
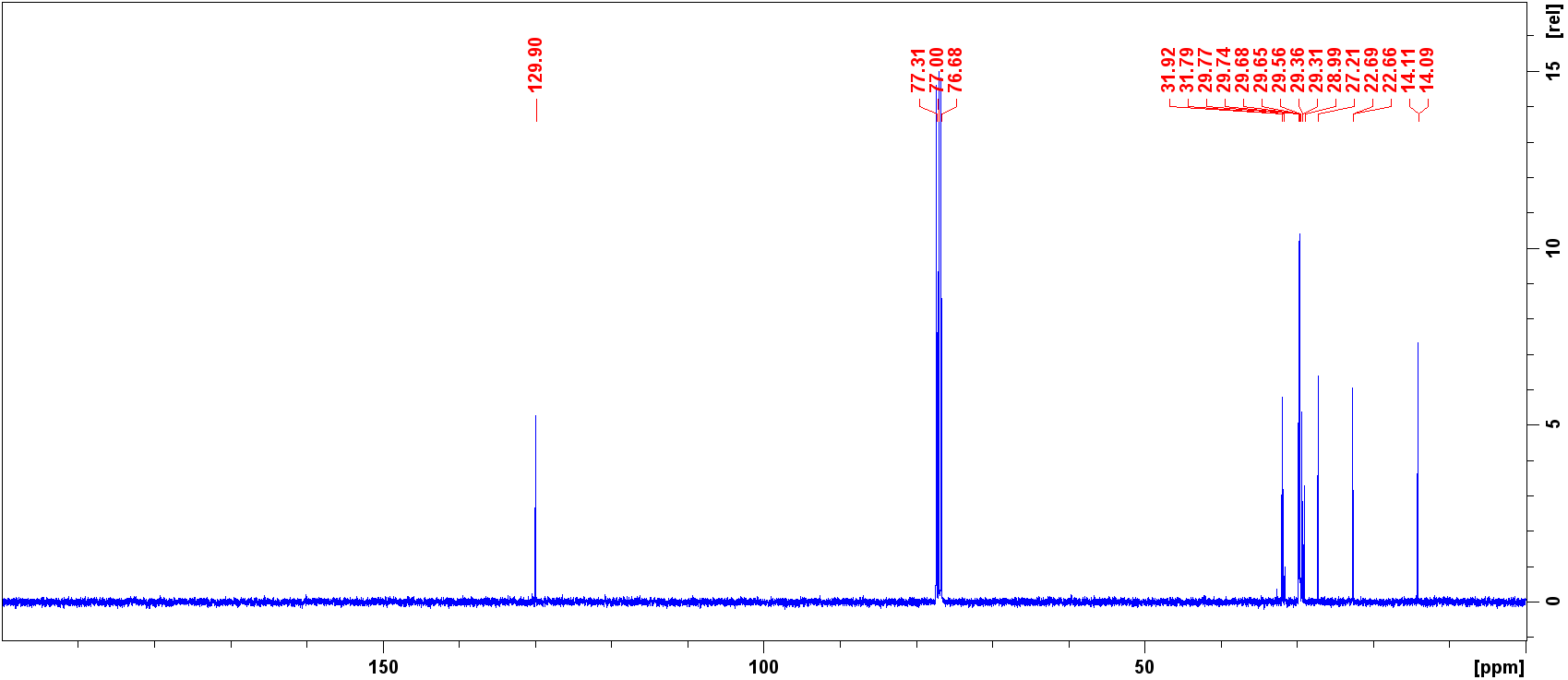
**

**Compound 24**

**
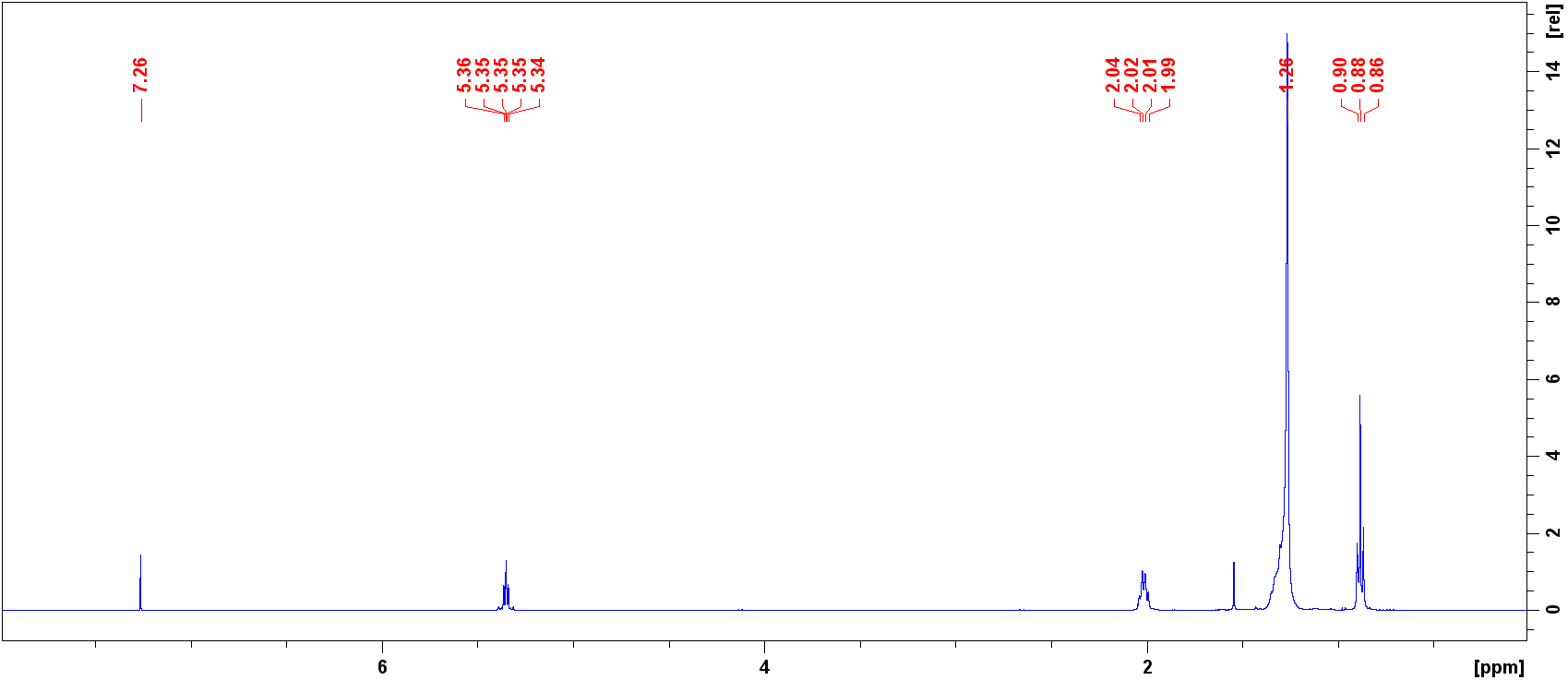
**

**
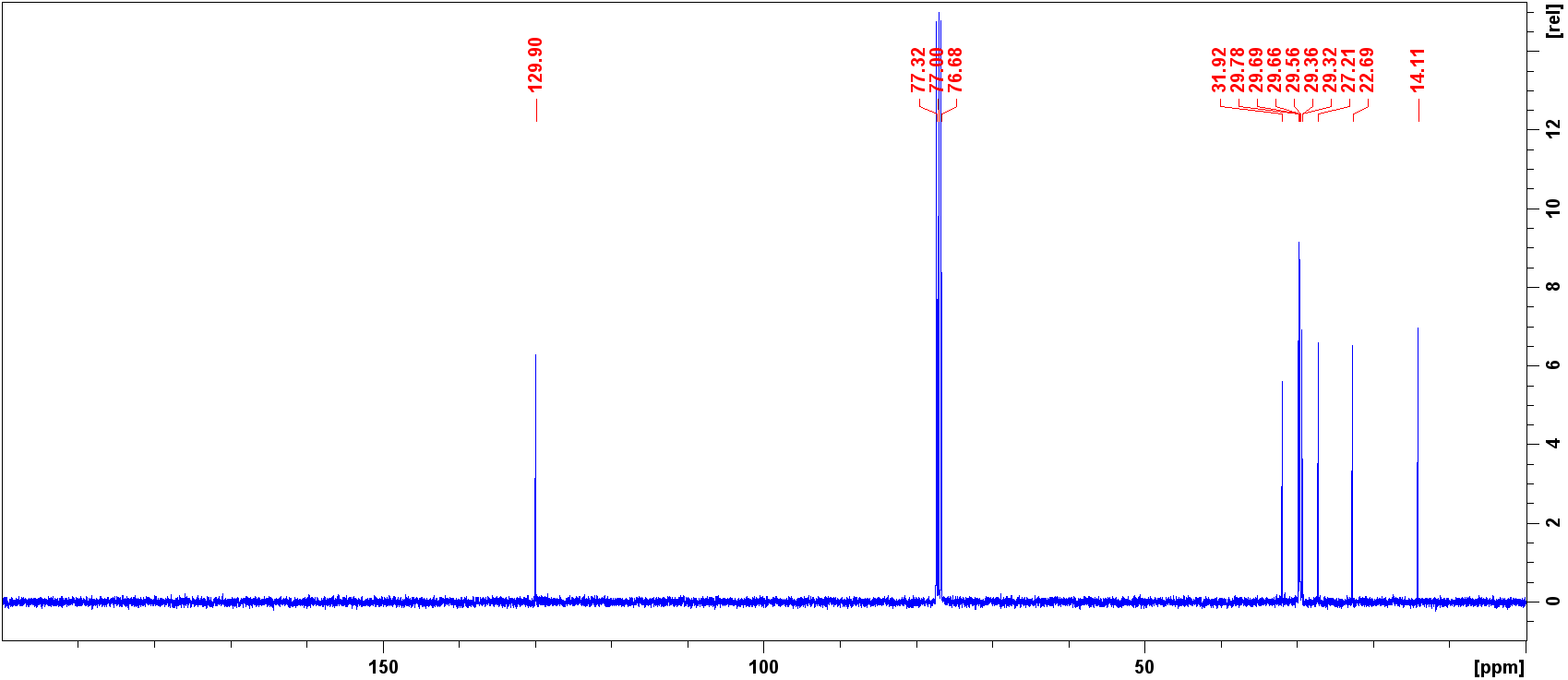
**

**Compound 25**

**
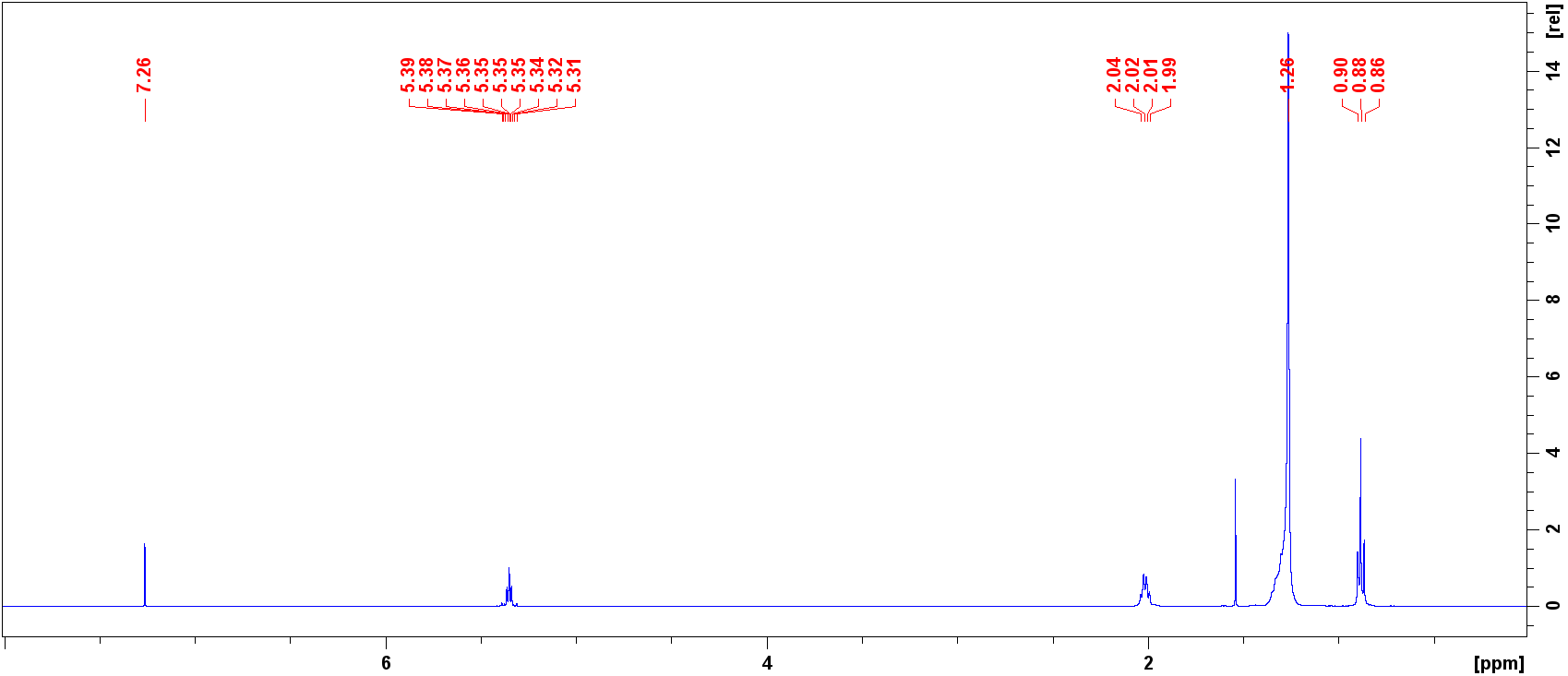
**

**
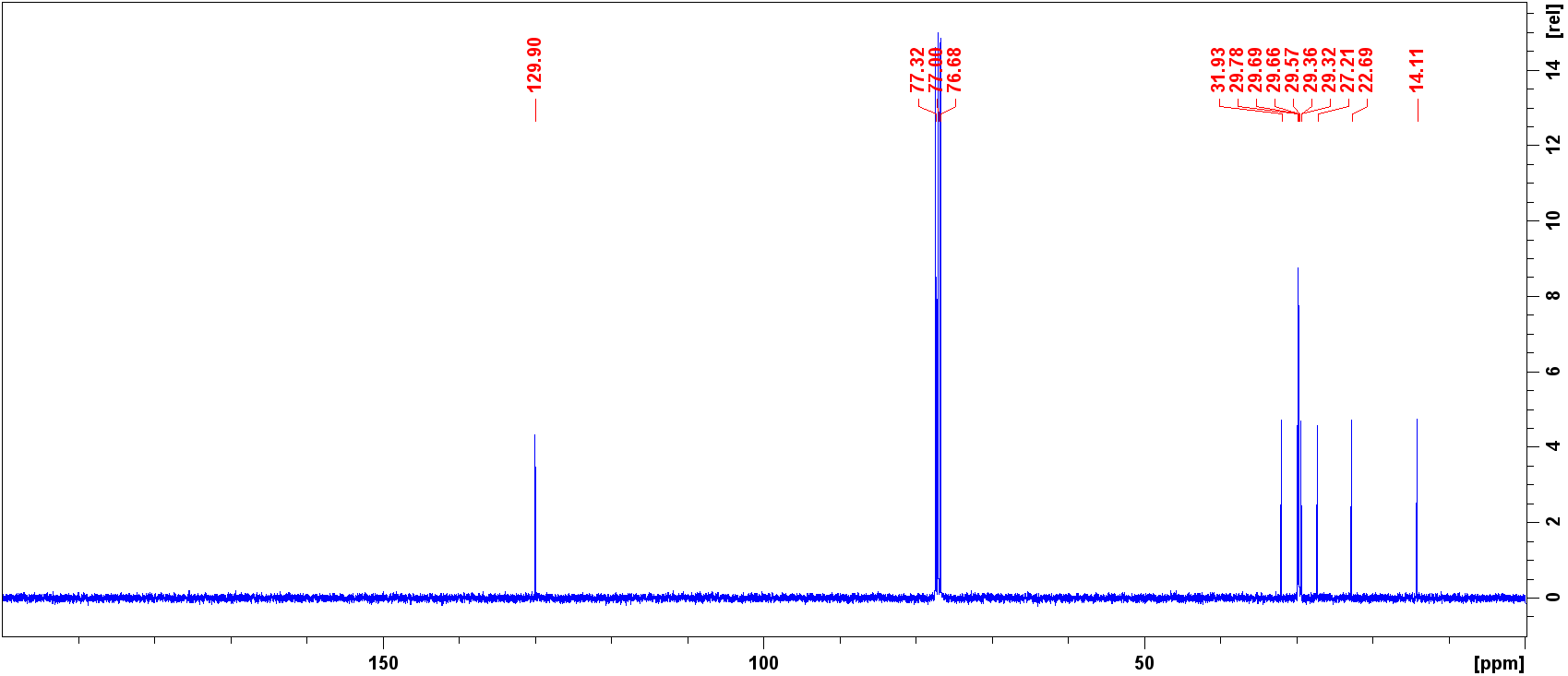
**

**Compound 26**

**
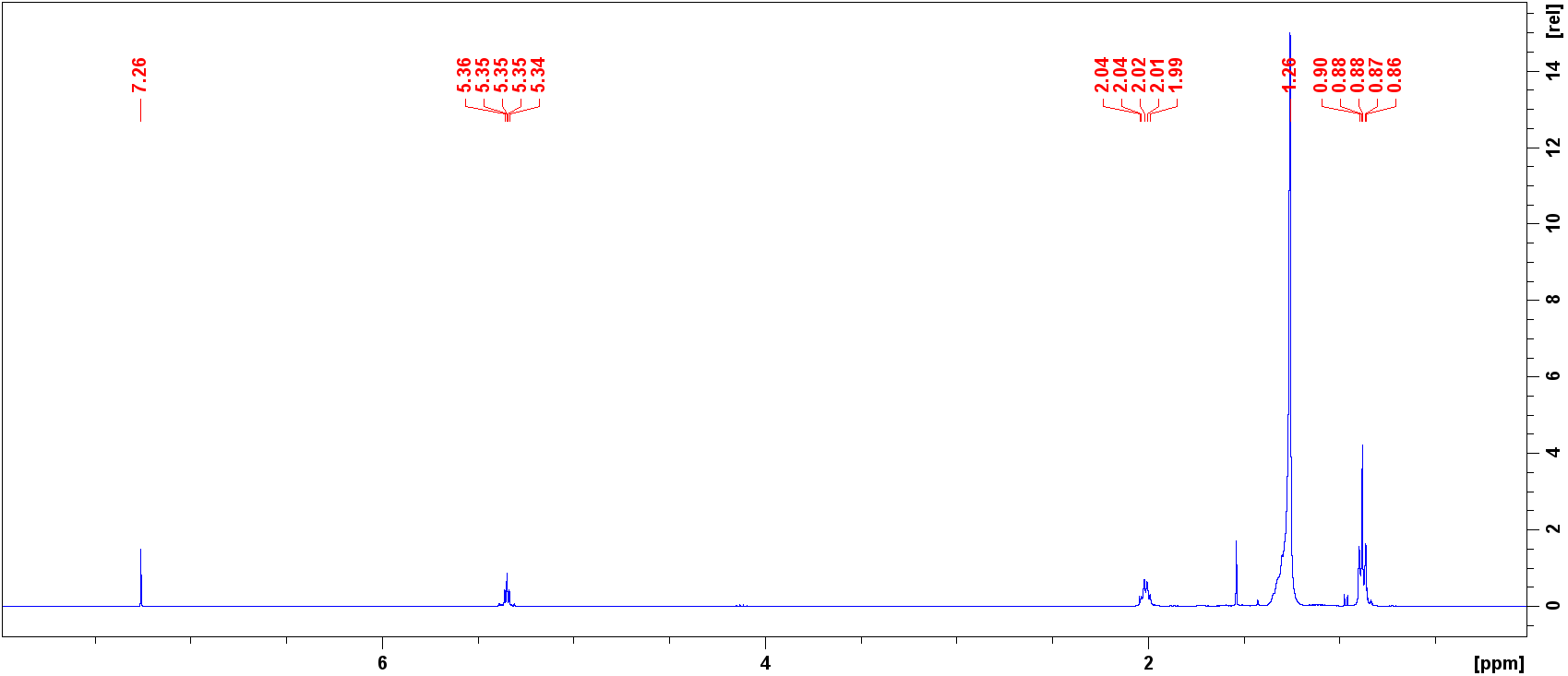
**

**
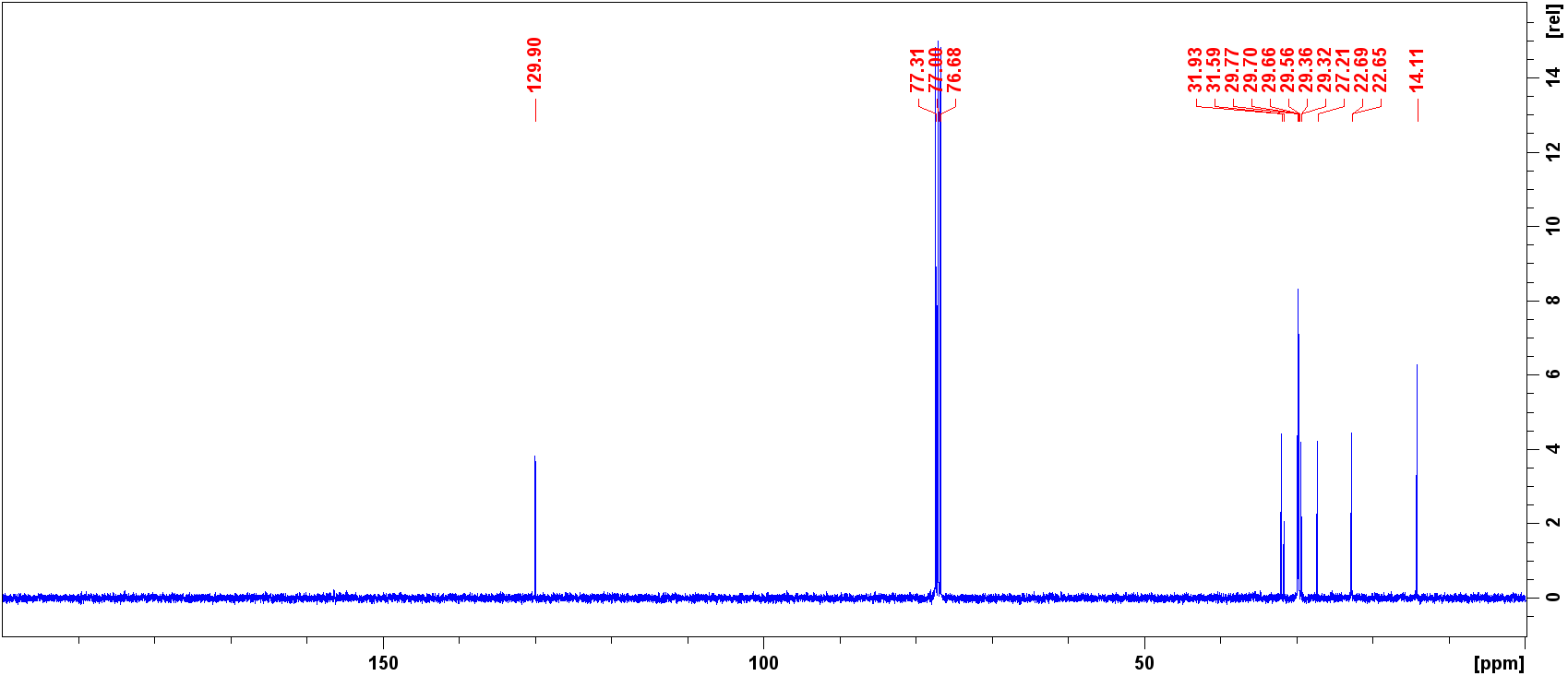
**

**Compound 27**

**
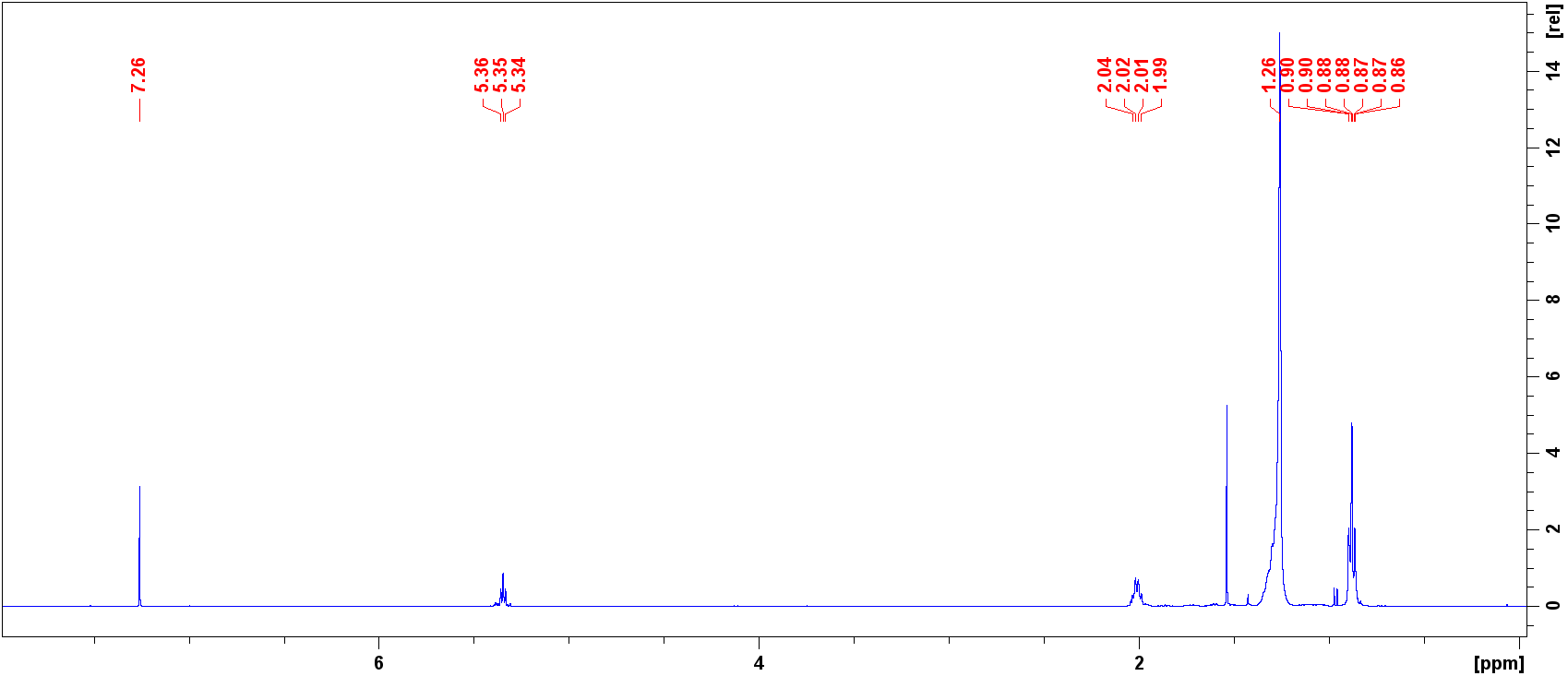
**

**
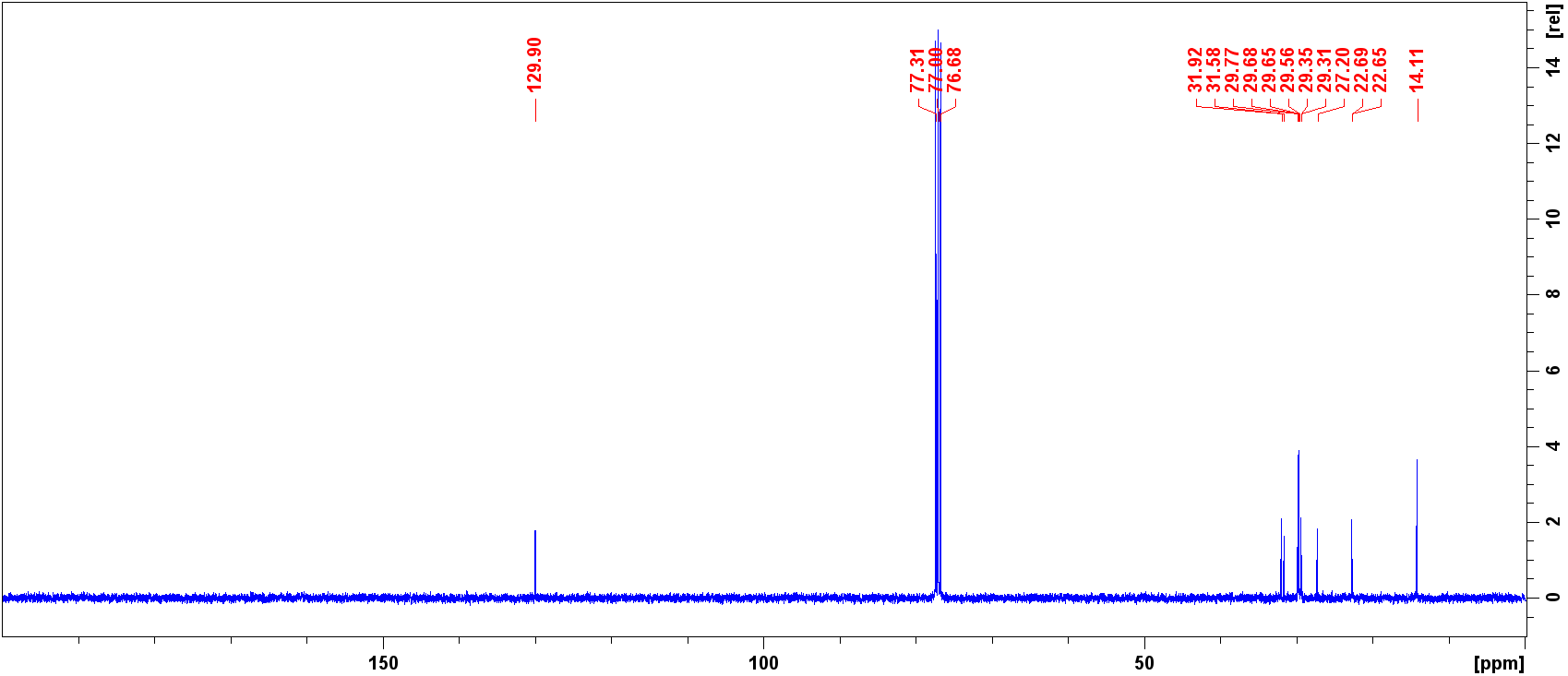
**

**Compound 28**

**
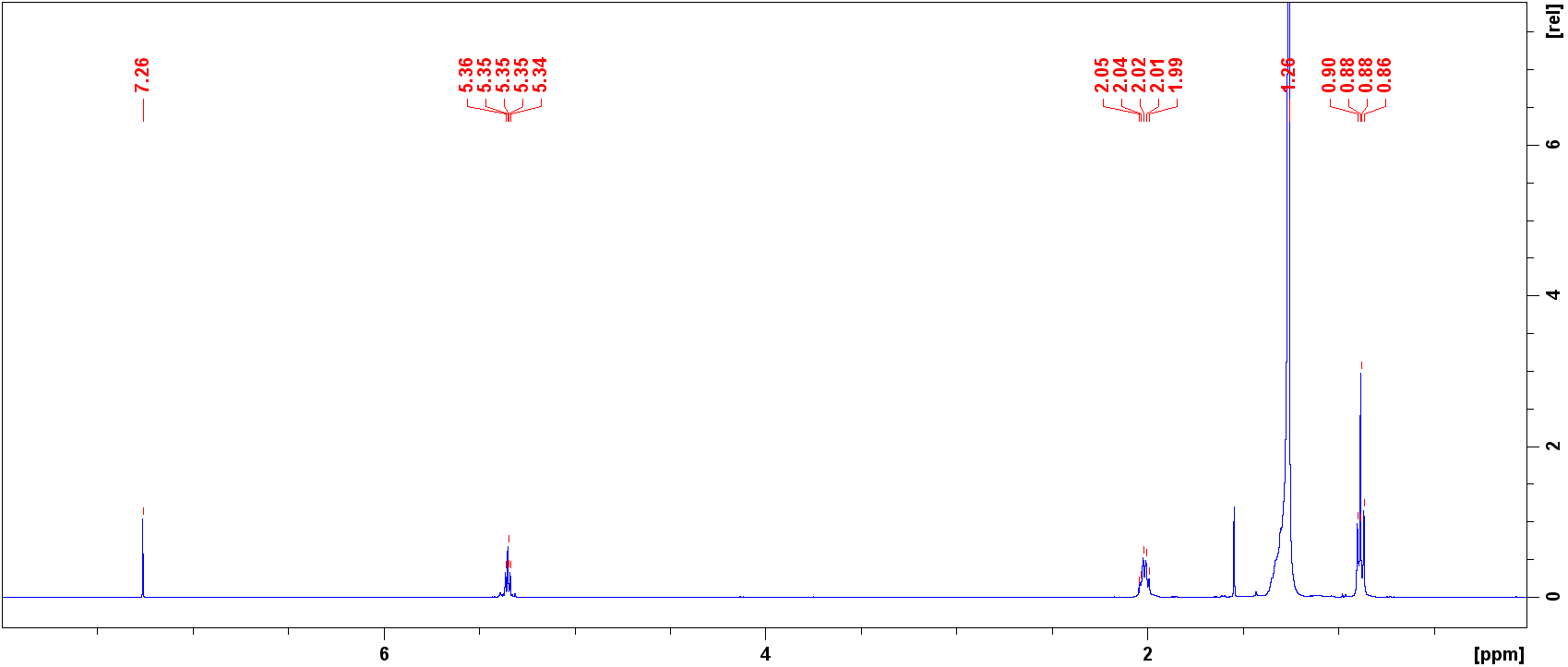
**

**
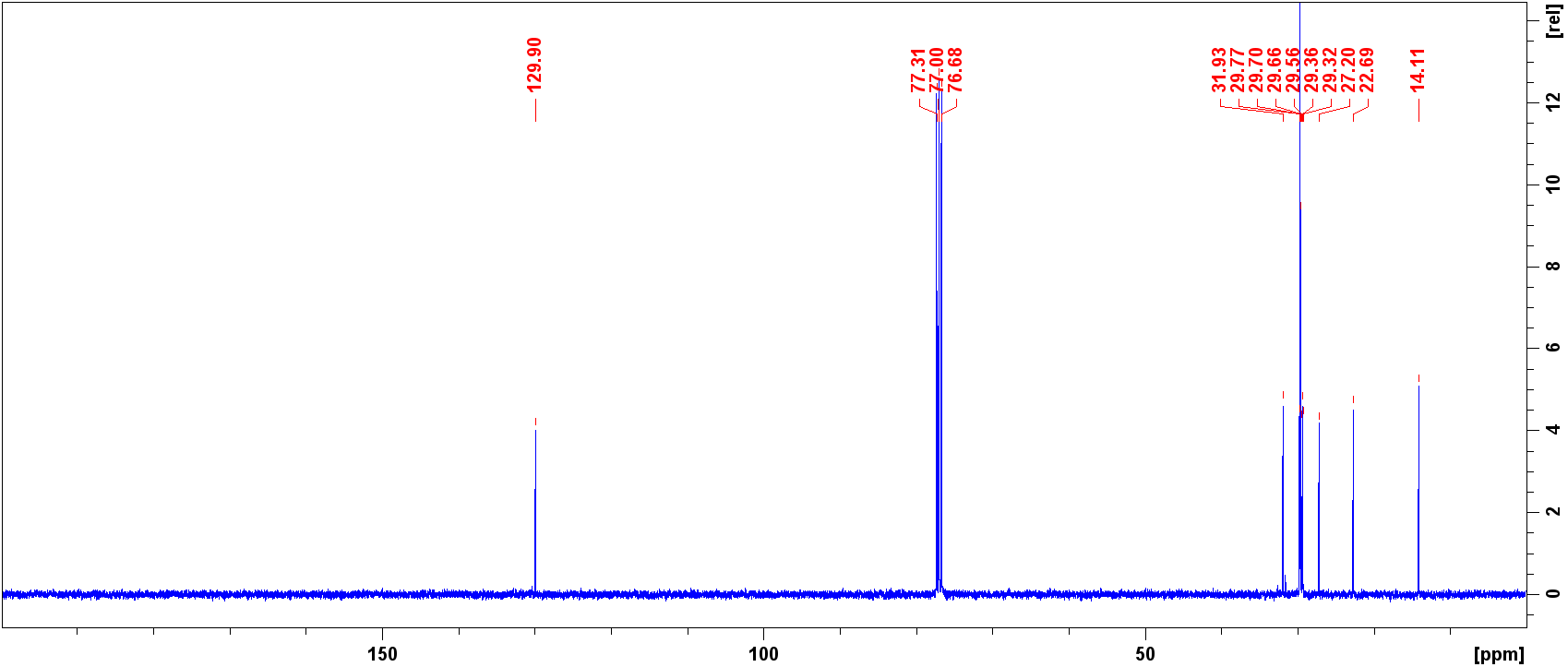
**
